# Transforming cancer immunotherapy: integration of distinct immune-based approaches as redefined dual immunotherapy with potential third-sensitizer

**DOI:** 10.1186/s40164-025-00705-9

**Published:** 2025-09-29

**Authors:** Yuqian Wang, Cheng Jiang, Huiling Zhou, Rui Han

**Affiliations:** 1 Department of Chinese Medicine Oncology, The First Affiliated Hospital of Naval Medical University, Shanghai, 200433 People’s Republic of China; 2 Department of Chinese Medicine, Naval Medical University, Shanghai, 200433 People’s Republic of China; 3 Department of Chinese Medicine Oncology, Naval Medical Center, Naval Medical University, Shanghai, 200433 People’s Republic of China; 4 School of Integrative Medicine, Shanghai University of Traditional Chinese Medicine, Shanghai, 201203 People’s Republic of China; 5 Traditional Chinese Medicine Department of Deyang People’s Hospital, Deyang, 618000 People’s Republic of China

**Keywords:** Dual distinct immunotherapy, Cancer immunotherapy, Synergistic mechanisms, Resistance overcoming, Combined therapeutic strategy

## Abstract

This review introduces a paradigm-shifting concept of Dual Distinct Immunotherapy (DDI), which strategically integrates two distinct immunotherapeutic modalities to overcome the limitations of current monotherapies and dual immune checkpoint inhibitor (ICI) combinations. The concept of DDI extends beyond traditional ICI combinations to encompass various innovative pairings: ICIs with oncolytic viruses (OVs), adoptive cell therapies (CAR-T/TIL), cancer vaccines, or cytokine therapies. These combinations demonstrate unique synergistic mechanisms and enhanced therapeutic potential through multi-faceted immune activation. Significantly, this work advances the field by analyzing potential third-agent sensitizers to complement DDI strategies. We systematically evaluate emerging candidates including PCNA inhibitors, HDAC inhibitors, and carbonic anhydrase inhibitors, focusing on their ability to modulate the tumor microenvironment and enhance immunotherapy responses. This "DDI + 1" approach targets alternative pathways to overcome resistance mechanisms and expand treatment efficacy to traditionally immunotherapy-resistant cancers. Through comprehensive analysis of preclinical evidence and ongoing clinical trials, we address critical challenges in immunotherapy, including primary and acquired resistance, cold tumor conversion, and pathway exhaustion. The review synthesizes current findings while proposing innovative solutions and future research directions. Our framework demonstrates how strategic integration of multiple immune-based approaches can significantly improve therapeutic outcomes across diverse cancer types, potentially revolutionizing cancer treatment paradigms. This concept of DDI, enhanced by rational third-agent selection, represents a promising direction for addressing urgent clinical needs in oncology. By establishing a theoretical foundation for this approach, we aim to guide future research and clinical applications in cancer immunotherapy.

## Introduction

As reported by the International Agency for Research on Cancer (IARC) and the American Cancer Society (ACS), nearly 20 million new cancer cases were diagnosed worldwide in 2022 [1, 2]. The most common cancers were lung cancer, with 2.5 million new cases, breast cancer with 2.3 million cases, and colorectal cancer with 1.9 million cases [3]. Moreover, it has been predicted that, by 2050, the number of new cancer cases will rise to 35 million [3]. For details, in 2022, approximately 10 million people succumbed to cancer, with lung cancer being the leading cause of death, resulting in 1.8 million deaths, followed by colorectal cancer with 900,000 deaths and liver cancer with 780,000 deaths [1, 2]. Asia experienced 49.2% of all new cancer cases and 56.1% of all cancer deaths, reflecting its large population size [4]. In contrast, Europe, which comprises only 9.6% of the global population, accounted for 22.4% of cancer cases and 20.4% of cancer deaths [4]. Undoubtedly, cancer is one of the greatest threats to human health.

As for the treatment of malignant tumor, immunotherapy (including ICIs, CAR-T, cancer vaccines, etc.) has doubtlessly emerged as a pivotal element in the management of advanced cancers, profoundly impacting prognosis and treatment modalities [5]. Such therapeutic approach offers several notable advantages over traditional chemotherapy and targeted therapy in treating advanced cancers. Its key benefit lies in leveraging the body's immune system to precisely target and eliminate cancer cells, potentially resulting in more durable responses [6]. Unlike chemotherapy, which attacks all rapidly dividing cells and can harm healthy tissues, immunotherapy enhances the immune system's natural cancer-fighting abilities, often resulting in fewer and less severe side effects [7]. Another significant advantage is the potential for long-lasting remissions [8]. Immunotherapy can train the immune system to remember cancer cells, allowing it to continue attacking any that reappear, even after treatment has concluded [9]. This contrasts with chemotherapy and targeted therapy, which typically require ongoing administration to maintain effectiveness. In addition, immunotherapy has also proven effective against various cancers that are resistant to traditional treatments. ICIs, for example, have significantly improved outcomes for patients with metastatic melanoma, non-small cell lung cancer (NSCLC), and renal cell carcinoma [10, 11]. Beyond traditional CAR-T cells, emerging platforms including CAR-NK cells [12], CAR-macrophages [13], and TCR-engineered T cells [14] are expanding the therapeutic arsenal against solid tumors. Furthermore, while targeted therapies aim to inhibit specific molecules involved in cancer growth, they can sometimes lead to resistance due to mutations in cancer cells [15]. Immunotherapy, however, engages a broader immune response that can adapt to changes in cancer cells, potentially overcoming such resistance [16].

Despite these advances, current immunotherapy faces significant limitations that represent critical knowledge gaps in the field. Primary resistance affects 60–70% of patients treated with immune checkpoint inhibitors, particularly those with "cold" tumors characterized by low immune cell infiltration and limited neoantigen burden [17]. Additionally, acquired resistance develops in approximately 40–50% of initial responders, often due to immune checkpoint pathway exhaustion, tumor microenvironment immunosuppression, and loss of tumor antigen presentation [18]. Immune-related adverse events (irAEs) pose another substantial challenge, occurring in 15–90% of patients depending on the specific agents used, with severe grade 3–4 toxicities affecting 10–55% of cases [19]. These adverse effects can affect multiple irAEs glands, sometimes requiring treatment discontinuation and immunosuppressive interventions. Furthermore, the limited efficacy of current dual immune checkpoint inhibitor combinations becomes apparent when resistance develops, leaving patients with few viable subsequent treatment options due to pathway exhaustion and cross-resistance mechanisms.

A critical knowledge gap exists in the systematic integration of distinct immunotherapeutic modalities beyond traditional checkpoint inhibitor combinations. While numerous studies have explored individual immunotherapy approaches, there is insufficient understanding of how to strategically combine fundamentally different immune-based therapies to create synergistic effects that can overcome the limitations of monotherapy and current dual checkpoint inhibitor approaches. The field lacks a comprehensive framework for what we term "Dual Distinct Immunotherapy" (DDI)—the strategic combination of two fundamentally different immunotherapeutic modalities such as combining immune checkpoint inhibitors with oncolytic viruses, adoptive cell therapies, cancer vaccines, or cytokine therapies. Additionally, the potential for incorporating non-immunotherapy sensitizers as third agents to further enhance DDI efficacy remains largely unexplored.

**Fig. 1:**
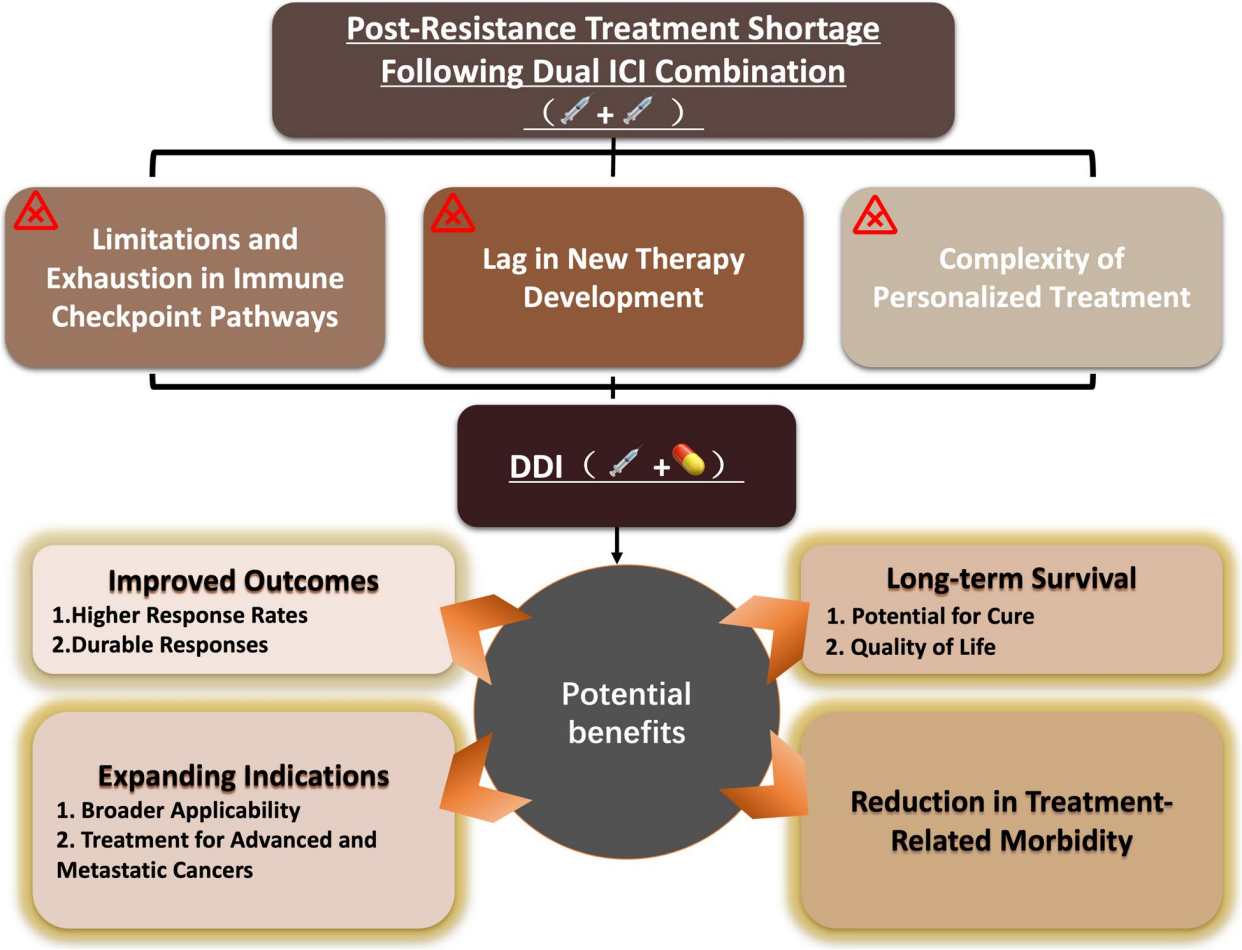
Dual distinct immunotherapy (DDI) contains multifaceted advantages. DDI combines two distinct immunotherapeutic strategies to enhance anti-cancer efficacy. The top panel highlights the challenges faced in the post-resistance treatment landscape following dual immune checkpoint inhibitor (ICI) therapy, including limitations in immune pathway exhaustion, delayed development of novel therapies, and the complexity of personalized treatments. Despite these obstacles, DDI holds promise by leveraging synergistic mechanisms to offer significant benefits: improved treatment outcomes through higher response rates and durable responses, expanding indications to address advanced and metastatic cancers, long-term survival potential with improved quality of life, and reduction in treatment-related morbidity

This review advances the field by introducing and systematically analyzing the novel concept of DDI, which extends beyond traditional dual checkpoint inhibitor combinations to encompass diverse immunotherapeutic pairings with distinct mechanisms of action. Our work addresses the identified knowledge gaps by: (1) establishing a theoretical framework for DDI that can overcome resistance mechanisms through multi-pathway immune activation; (2) providing comprehensive analysis of synergistic mechanisms underlying various DDI combinations including ICIs with oncolytic viruses, CAR-T cells, tumor-infiltrating lymphocytes, and RNA-based cancer vaccines; (3) systematically evaluating the potential of third-agent sensitizers such as PCNA inhibitors, HDAC inhibitors, and carbonic anhydrase inhibitors to create "DDI + 1" approaches; and (4) analyzing current clinical evidence and proposing future research directions for these innovative combination strategies. By establishing DDI as a new paradigm that can convert "cold" tumors to "hot" tumors, overcome resistance mechanisms, and expand the therapeutic window for immunotherapy-resistant cancers, this work provides a roadmap for addressing the most pressing limitations in current cancer immunotherapy and offers a foundation for developing next-generation combination approaches that may significantly improve patient outcomes. Therefore, the targeted, adaptable, and long-lasting nature of immunotherapy makes it a promising option for managing advanced cancers, offering hope for better outcomes and improved quality of life for patients [20]. Therefore, new combination therapy approaches based on tumor immunotherapy are continuously being explored and evaluated in an effort to further enhance efficacy and clinical benefits for patients (Fig. 1).

### Advancing cancer treatment through dual ICI combinations

Fortunately, combination therapy regimens based on immunotherapy (such as ICI + targeted therapy, ICI + chemotherapy, and ICI + ICI) have been shown to be superior to traditional systemic treatments (such as targeted therapy and chemotherapy) in many advanced malignancies [21, 22]. Among these, the dual immune checkpoint inhibitor combination has proven to be a novel and promising immunotherapy approach [23]. For instance, the recent CheckMate-9DW trial investigated the efficacy and safety of combination therapies involving ICIs for treating advanced or metastatic cancer [24]. For details, in this study, the combination of ICIs (Nivolumab [PD-1 inhibitor] plus ipilimumab [CTLA-4 inhibitor]) in this achieved an overall response (OR) rate of 45% compared to 28% with monotherapy (Lenvatinib or Sorafenib [target therapy]) (p < 0.01). Median progression-free survival (PFS) for the combination therapy group was 9.4 months, compared to 5.6 months for the monotherapy group (p < 0.01). The median OS for patients receiving combination therapy was 21.1 months, versus 15.8 months for those on monotherapy (p = 0.02). 30% of patients in the combination therapy group had a durable response lasting more than 12 months, compared to 18% in the monotherapy group (p = 0.03). As for Safety Results, the incidence of TRAEs (Treatment-Related Adverse Events) was 78% in the combination therapy group, compared to 62% in the monotherapy group (p < 0.01). Severe adverse events occurred in 32% of patients receiving combination therapy, compared to 15% in the monotherapy group (p < 0.01).

By combining these two ICIs, the goal of CheckMate-9DW trial is to enhance T-cell activation and proliferation, thereby creating a synergistic effect [25]. This approach targets different pathways that tumors use to evade the immune system, ultimately improving the overall antitumor response. Combining different types of ICIs can activate the immune system through multiple mechanisms, leading to a stronger anti-tumor response, and potentially bringing higher OR rates and prolonged PFS and OS.

The evolution of dual immune checkpoint inhibitor combinations marks a major breakthrough in cancer treatment [20]. These therapies utilize two distinct ICIrs to boost the immune system's ability to combat cancer cells [26]. By engaging multiple pathways, they aim to surpass the limitations of single-agent therapies and enhance patient outcomes [27]. Other clinical trials have also shown encouraging results, including higher response rates and extended survival in various cancers such as melanoma, lung cancer, and renal cell carcinoma [28]. Nonetheless, tackling resistance development continue to be significant challenges in this area of research [29].

### Post-resistance treatment shortage following dual ICI combination

This resistance leaves patients with limited or no viable treatment alternatives, which is a significant concern in oncology [30]. When cancer progresses beyond the efficacy of dual immune checkpoint inhibitors, it can lead to disease advancement and poorer patient outcomes [31].

*A. Limitations and Exhaustion in Immune Checkpoint Pathways* Currently, identified immune checkpoints include PD-1, TIM-3, LAG-3, BTLA, and others [32]. However, the FDA-approved inhibitors for cancer treatment target only a few checkpoints, such as CTLA-4, PD-L1 and PD-1 [33]. Inhibitors for other immune checkpoints are still in the research phase. Therefore, while the combination of ICIs increases immune regulatory pathways and accelerates T-cell activation, it also hastens the exhaustion of these pathways [34, 35]. Once these pathways fail, there are very few subsequent immune checkpoint blockade treatment options available.

*B. Lag in New Therapy Development* The development and clinical translation of novel ICIs represents a significant bottleneck in addressing post-resistance scenarios. Although numerous next-generation ICIs targeting alternative immune checkpoints are currently under investigation, the transition from preclinical research to clinical application typically requires 10–15 years [36]. This protracted timeline encompasses multiple phases of development including target validation, lead compound optimization, extensive preclinical safety and efficacy testing, and sequential Phase I, II, and III clinical trials. The regulatory approval process adds additional years to this timeline, during which patients who have exhausted current dual ICI combinations face limited therapeutic options [31].

*C. Complexity of Personalized Treatment* The heterogeneous nature of cancer and the intricate variability in individual immune responses create substantial challenges in developing personalized therapeutic strategies for patients who develop resistance to dual ICI combinations. Tumor heterogeneity manifests at multiple levels, including genetic mutations, epigenetic modifications, protein expression patterns, and microenvironmental characteristics, all of which can influence the mechanisms underlying ICI resistance [31]. The heterogeneous tumor immune microenvironment significantly impacts clinical outcomes [37], particularly in breast cancer where heterogeneity drives personalized therapy requirements [38]. Intratumoral heterogeneity adds another layer of complexity, as different regions within the same tumor may exhibit distinct resistance patterns, making it difficult to predict treatment outcomes based on single biopsy samples. Patient-specific factors, including HLA typing, germline genetic variations, prior treatment history, performance status, comorbidities, and individual immune system characteristics, significantly influence both treatment efficacy and resistance development patterns [39].

After resistance to combination ICIs, patients may have limited treatment options, as they may no longer respond to ICIs targeting similar pathways. This can severely compromise the clinical benefits for patients [40]. Addressing this dilemma requires efforts on multiple fronts: developing multi-target combination therapies by creating more drugs that target different immune checkpoints or immune pathways and exploring the best combinations and timing for their use [41]. Developing next-generation ICIs targeting different pathways beyond PD-1, PD-L1, and CTLA-4 is also inevitable [42]; utilizing biomarkers to predict the occurrence of resistance by detecting patients' biomarkers and adjusting treatment strategies in advance [43]; and applying extra therapies such as cell therapies, cancer vaccines, and OVs to provide new treatment options for resistant patients [44].

Thus, a novel treatment strategy named “Dual Distinct Immunotherapy”, presented in this article, holds significant potential to reduce resistance development and enhance the efficacy of immunotherapy through multiple pathways by targeting diverse immune mechanisms simultaneously. However, further research is essential to fully understand specific resistance mechanisms and optimize DDI strategies to address them effectively.

The critical importance of this issue underscores the need for innovative strategies and therapies to address resistance and ensure ongoing treatment possibilities for patients who have exhausted existing immunotherapy options. This necessitates research into alternative therapeutic combinations, the development of new drugs, and a deeper understanding of resistance mechanisms to enhance future cancer treatment protocols.

## Dual distinct immunotherapy

Dual Immunotherapy (DDI) refers to the strategic combination of two distinct types of antitumor immunotherapies to synergistically enhance the immune system's ability to recognize, attack, and destroy cancer cells more effectively[45]. This approach leverages different mechanisms of action from each therapy to improve treatment outcomes and overcome resistance that might occur with monotherapy. Dual Immunotherapy involves the integration of separate immunotherapeutic modalities. These combinations can be designed to target various aspects of the immune response to cancer, aiming to create a more robust and sustained antitumor effect. The types of combinations used in tumor dual immunotherapy can include ICI plus cancer vaccine, ICI plus adoptive cell therapy, ICI plus Cytokine therapy, etc. [46]. Recent preclinical studies have demonstrated synergistic efficacy of novel combinations, including simultaneous anti-TGF-β/VEGF bispecific antibody with PD-1 blockade [47] and anti-TGF-β/PD-L1 bispecific antibody promoting T cell infiltration in triple-negative breast cancer [48].

Dual immunotherapy shows promise for expanding treatment indications. Through the strategic combination of different immunotherapies, it may potentially target a broader range of cancers, including some traditionally resistant to single-agent immunotherapy [49]. This approach may particularly benefit patients with advanced and metastatic cancers, where traditional treatments often demonstrate limited effectiveness [16]. However, comprehensive clinical validation is required to establish the full scope of DDI applications and its efficacy across diverse cancer types.

Such combination represents a novel and promising approach in cancer treatment, aiming to harness and amplify the body's immune response through multiple immunotherapeutic strategies [50]. This dual approach is designed to overcome the limitations and resistance associated with single-agent immunotherapies by attacking the tumor through multiple mechanisms. By integrating different therapeutic modalities, Tumor Dual Immunotherapy can potentially achieve better clinical outcomes, including higher response rates, prolonged survival, and improved quality of life for patients with various types of cancer [49, 51].

**Fig. 2:**
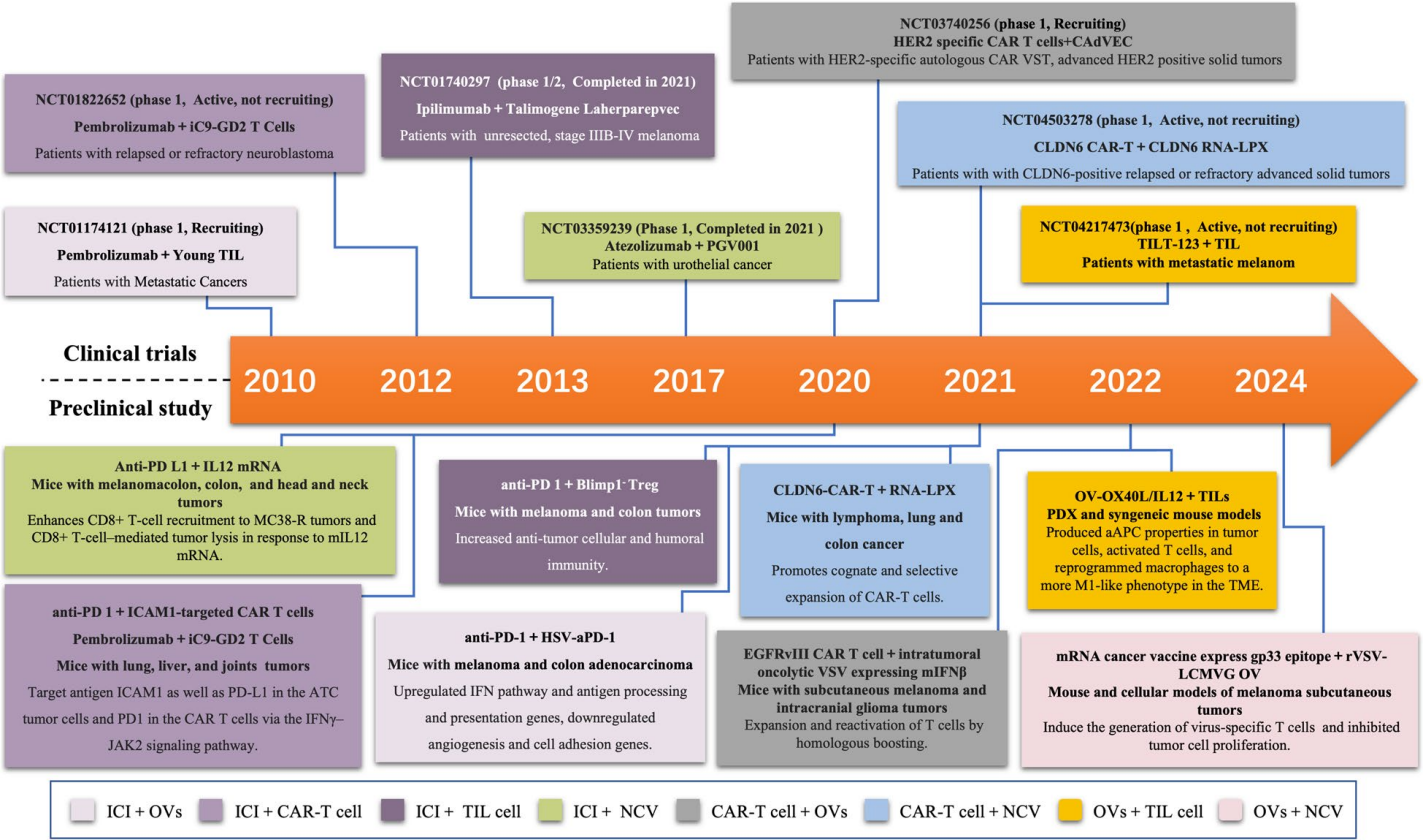
Milestones of DDI Development: The boxes above the timeline display the representative clinical trials of different types of DDI regimen, while the boxes below highlight the most recent preclinical studies. Each box is color-coded to indicate the different combination therapies being explored. Notably, there are currently no ongoing clinical trials involving OVs in combination with NCV

DDI has achieved remarkable progress over the past decade, transitioning from basic research in the early 2010s to clinical trials. By 2024, various DDI combination therapies have advanced into multiple clinical trial phases, showcasing significant potential. Current research explores a wide range of therapeutic combinations, including ICIs with OVs, ICIs with T-cell therapies (such as CAR-T or TIL cells), and ICIs with RNA-based cancer vaccines. Additionally, emerging strategies like combining OVs with cell-based therapies or CAR-T cells with RNA-based cancer vaccines are being investigated. These approaches demonstrate strong synergistic effects in enhancing tumor immune recognition, overcoming resistance to monotherapies, and broadening the scope of treatable cancer types. The rapid progression of clinical trials has been supported by recent advances in foundational research, particularly in the modulation of the tumor immune microenvironment and the identification of novel immune targets. The application of DDI has expanded from traditionally immune-sensitive cancers to include less responsive cancers such as head and neck cancer, urothelial carcinoma, and lung cancer. Looking ahead, as more combination strategies progress to late-stage clinical trials and innovative approaches continue to be explored, DDI is poised to further improve patient survival rates and quality of life, paving the way for new possibilities in cancer treatment (Fig. 2).

While DDI represents a promising paradigm for cancer immunotherapy, it is important to acknowledge that resistance mechanisms in cancer are complex and multifaceted. DDI may help delay the onset of resistance, reduce its severity, or overcome certain resistance pathways through multi-mechanism approaches, but complete prevention of all resistance mechanisms remains an ongoing challenge requiring continued research and development. The evolution of tumor cells, immune evasion strategies, and the dynamic nature of the tumor microenvironment suggest that DDI, while advantageous, should be viewed as part of a broader strategy that may include DDI + 1 approaches and continuous adaptation of treatment protocols based on emerging resistance patterns. Advancing Cancer Treatment Through Dual ICI Combinations.

Therefore, this work aims to introduce and establish the paradigm-shifting concept of DDI, which strategically integrates two distinct immunotherapeutic modalities to overcome the limitations of current monotherapies and dual immune checkpoint inhibitor combinations. Through systematic analysis of preclinical evidence and ongoing clinical trials, we seek to demonstrate how DDI can address critical challenges in immunotherapy, including primary and acquired resistance, cold tumor conversion, and pathway exhaustion. Equally important, this work tries to advance the field by evaluating potential third-agent sensitizers, including PCNA inhibitors, HDAC inhibitors, and carbonic anhydrase inhibitors, to complement DDI strategies in what we term the “DDI + 1” approach. By synthesizing current findings and proposing innovative solutions, a theoretical foundation for DDI has been built, and provides guidance for future research directions and clinical applications in cancer immunotherapy, ultimately aiming to revolutionize cancer treatment paradigms, sitimulating the exploration of novel anti-cancer mechanisms, and improve therapeutic outcomes across diverse cancer types.

### ICI combined with OVs

**Fig. 3:**
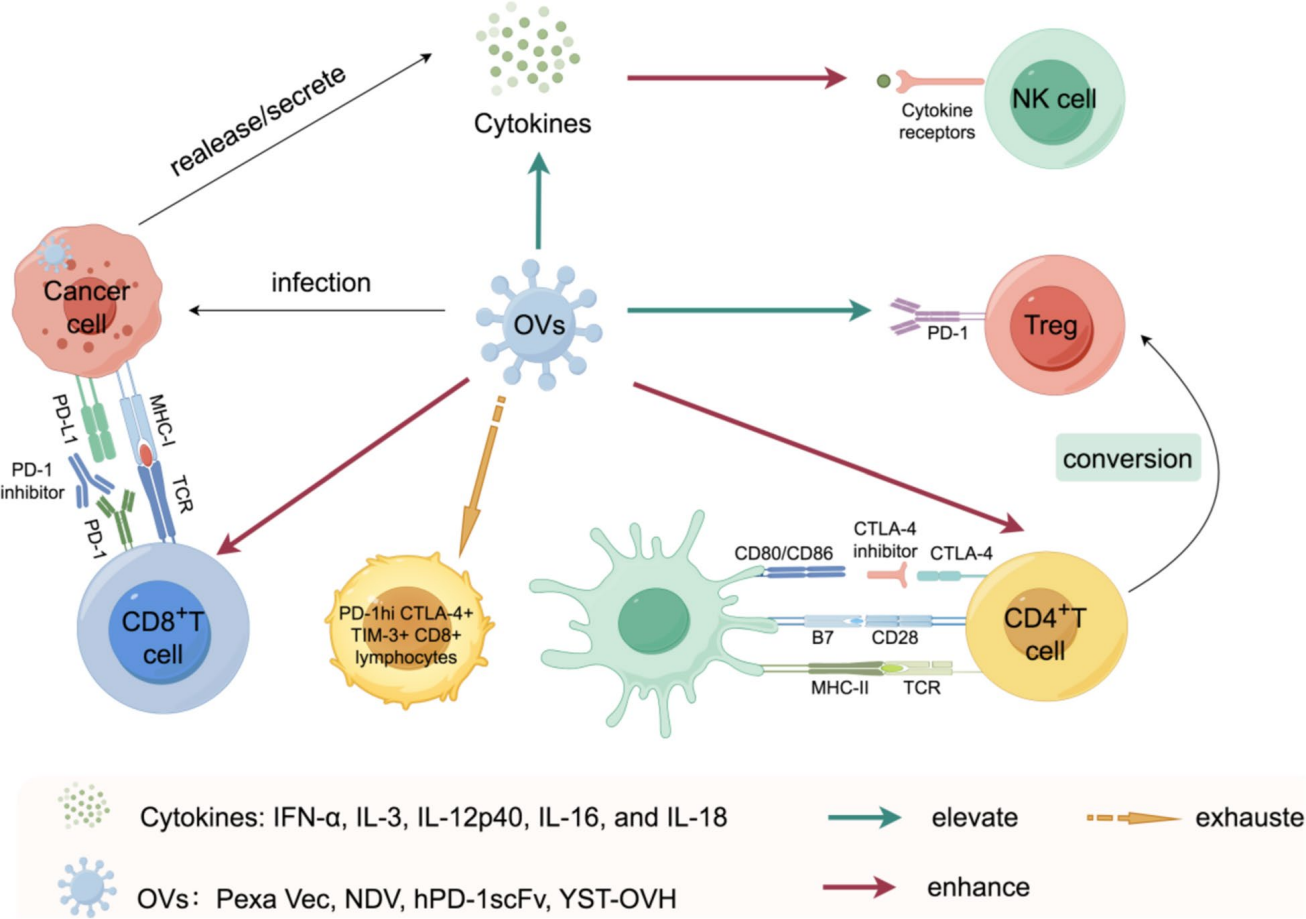
Synergistic Effects of Oncolytic Viruses (OV) in Enhancing Anti-Tumor Immunity and Overcoming Immune Suppression. A newly discovered OV, named “OactivatePexastimogene Devacirepvec” (Pexa Vec), can elevate the expression levels of antitumor immune cytokines, including IFN-α, IL-3, IL-12p40, IL-16, and IL-18, and activate NK cells as well as CD4 + and CD8 + T cells in tumor tissues. Another, known as the “Newcastle disease virus” (NDV), has been found to exert a synergistic effect when administered in combination with a CTLA-4 inhibitor. Meanwhile, evidence has also indicated that OVs can further improve the early infiltration of CD8 + T cells into tumors and reduce the infiltration by highly suppressive PD-1 + Treg cells. The single-chain variable fragment against human PD-1 (hPD-1scFv), referred to as YST-OVH, enhances the effector and memory functions of tumor-infiltrating CD8 + lymphocytes and mitigates the exhaustion of PD-1hi CTLA-4 + TIM-3 + CD8 + lymphocytes

OVs are a unique class of virus that can specifically attack and eliminate cancer cells without harming normal tissues. This specificity makes oncolytic virotherapy a highly promising strategy in the fight against cancer [52–55]. OVs encompass a wide range of types, including reovirus, herpes simplex virus, adenovirus, vaccinia virus, coxsackievirus, poliovirus, Seneca Valley Virus (SVV), and others [56]. In addition, while direct oncolysis was originally regarded as the primary mechanism of action for OVs, it is now recognized that the activation of anti-tumor immune responses plays a crucial role in the therapeutic efficacy of OVs. This immune modulation has become an integral component of oncolytic virotherapy [57, 58]. Thus, OVs present a compelling strategy for localized immunomodulation, particularly when combined with ICIs. This combination has the potential to further amplify the immune response, offering a promising approach to cancer treatment. For instance, Pexastimogene Devacirepvec (Pexa Vec), as an newly discovered OV [59, 60], has shown the ability to activate natural killer (NK) cells as well as CD4^+^ and CD8^+^ T cells within tumor tissues. Additionally, it has been reported to elevate the expression levels of key anti-tumor immune cytokines, such as IL-18, IL-16, IL-12p40, IL-3 and IFN-α in patients with advanced solid tumors [61]. OVs can be essential in encouraging immune cell infiltration in tumors with sparse immune cell presence, often called the 'immune desert' phenotype [62]. Research increasingly demonstrates that combining these two approaches creates synergistic effects that may surpass the therapeutic benefits of either treatment alone (Fig. 3).

#### Synergistic mechanisms of immune modulation

Evidence has demonstrated that ICIs tend to have reduced efficacy when applied to treat cancers characterized by limited immune cell infiltration, often referred to as the "immune desert" phenotype. However, OVs have been shown to stimulate the recruitment of immune cells into the tumor microenvironment (TME). This increased immune cell presence creates an optimal environment, thereby enhancing the effectiveness of immune checkpoint blockade (ICB) therapy [63–65]. For instance, newcastle disease virus (NDV), as a novel OV, has been found to exert synergistic effect in a combinational administration with CTLA-4 inhibitor [66]. In other words, NDV can enhance the capability of CTLA-4 inhibitor on conquering tumor metastasis and improving long-term survival of mice with colon cancer or melanoma, by increasing tumor infiltration with activated lymphocytes, and systemic tumor inflammatory effects [66]. Evidence has also indicated that OVs can further improve the early tumor infiltration of CD8^+^ T cells and educe tumor infiltration of higly suppresssive PD-1^+^ regulatory T cells (Tregs). This effect has been observed in mice with melanoma treated in conjunction with ICIs [67].

Based on the concept of combinding OVs and ICIs, oncolytic virotherapy which uses single-chain variable fragment (scFv)-armed OVs targeting inhibitory immune checkpoints has also been found to act as an effective strategy for cancer immunotherapy. The convergence of oncolytic virotherapy and immune checkpoint modulation has led to an innovative therapeutic strategy involving engineered viruses armed with scFv targeting immune checkpoints. This strategy leverages the natural tumor-selective properties of OVs while delivering targeted immunomodulatory molecules directly to the tumor microenvironment. By incorporating carefully designed scFv molecules, this approach enables precise targeting of immune checkpoint pathways, thereby enhancing anti-tumor immune responses. Therefore, a study developed a functional humanized scFv targeting human PD-1, known as YST-OVH, which demonstrated potent and durable antitumor effects. This therapeutic effect was linked to the expansion and enhancement of effector and memory functions in tumor-infiltrating CD8^+^ T lymphocytes, along with a reduction in exhausted PD-1^hi^ CTLA-4^+^ TIM-3^+^ CD8^+^ lymphocytes [68]. In addition, YST-OVH proved effective not only in highly immunogenic tumors but also in less immunogenic, "cold" tumors. These findings suggest that YST-OVH therapy could be a promising candidate for treating poorly immunogenic tumors, offering a novel approach to overcome immune resistance in such malignancies [68].

#### Application of OVs in combination with ICIs in clinical trials

The therapeutic potential of combining OVs with ICIs has been validated through several key clinical investigations, with Talimogene Laherparepvec (T-VEC) leading the way as the first approved viral immunotherapy. The clinical trial landscape has yielded particularly noteworthy results in multiple cancer types. A Phase 1b trial (ClinicalTrials.gov Identifier: NCT02263508, MASTERKEY-265) assessed the combination of intratumoral T-VEC administration with pembrolizumab, an anti-PD-1 antibody. The results were particularly promising, showing a complete response (CR) rate of 33% and an objective response rate of 62% in patients with advanced melanoma [69]. The responders showed an increased population of circulating CD4^+^ and CD8^+^ T cells, along with enhanced CD8^+^ T cell infiltration into tumor tissues [69]. Notably, the phase 1b component of this trial demonstrated significant improvement with the combination compared to historical pembrolizumab monotherapy data. The ORR of 62% with T-VEC plus pembrolizumab substantially exceeded the 33–34% ORR typically observed with pembrolizumab alone in advanced melanoma. Similarly, the complete response rate of 33% with combination therapy represented approximately a three-fold improvement over the 6–12% CR rates seen with pembrolizumab monotherapy. These comparisons highlight the potential synergistic effect when combining oncolytic virotherapy with checkpoint inhibition.

Another Phase 1b/3 multicenter randomized trial (ClinicalTrials.gov Identifier: NCT02626000) investigated the combination of T-VEC and pembrolizumab in patients with recurrent or metastatic squamous cell carcinoma of the head and neck (R/M HNSCC). The results revealed an OR rate of 13.9% (assessed using the immune-related RECIST criteria), with a median PFS of 3.0 months (95% CI 2.0–5.8) and a median overall survival (OS) of 5.8 months in the five patients who received the combination treatment [70]. In addition, Enadenotucirev, as an Ad11p/Ad3 chimeric OVs. its safety and tolerability have also been trialed when combined with nivolumab for treating metastatic or advanced epithelial tumours (ClinicalTrials.gov Identifier: NCT02636036). Briefly, the OR rate was 2%, with a single patient achieving a partial response (PR), lasting for 9.8 months. The clinical benefit rate, defined as CR, PR at any time, or stable disease (SD) for at least 12 weeks, was 6% (3/47). The median OS was 16.0 months, with 79% survival at 6 months and 69% at 12 months [71].

A phase 1 study (ClinicalTrials.gov Identifier: NCT02043665) evaluated CAVATEK (CVA21), an unmodified coxsackievirus A21, in combination with pembrolizumab across two distinct cancer types [72]. In the NSCLC cohort, the combination therapy achieved an OR rate of 9%, comprising three ORs and one PR. A notable aspect of these responses was their durability, with all four responding patients maintaining their clinical benefit for six months or longer. The urothelial cancer cohort demonstrated even more promising results, with an OR rate of 20%. This included three CRs and four PRs. The durability profile remained strong in this group, with six patients (17% of the cohort) achieving sustained clinical benefit. The median time to response in this cohort was 3.0 months [73]. These results highlight the potential of combining CVA21 with pembrolizumab as an effective treatment strategy for metastatic NSCLC and urothelial cancer. The durable responses observed indicate that this combination therapy may offer long-term benefits for patients (Table 1).

**Table 1:** Clinical trial of ICIs combination therapy with OVs for solid tumor Treatment

Trial number	Launch	Phase	Study status	ICI agent	OVs agent	Tumor type	Rfs
NCT01740297	2012	1b/2	Completed (March 2021)	Ipilimumab	Talimogene Laherparepvec (T-VEC)	Melanoma	[347]
NCT02414269	2014	1b	Terminated	Pembrolizumab	Talimogene Laherparepvec (T-VEC)	Melanoma	[348]
NCT02965716	2017	II	Active, not recruiting	Pembrolizumab	Talimogene Laherparepvec (T-VEC)	Melanoma	[349]
NCT03069378	2017	II	Active, not recruiting	Pembrolizumab	Talimogene Laherparepvec (T-VEC)	Sarcoma Epithelioid Sarcoma Cutaneous Angiosarcoma	[350]
NCT02626000	2018	Ib	Completed(August 2020)	Pembrolizumab	Talimogene Laherparepvec (T-VEC)	Carcinoma of the Head and Neck	[351]
NCT04185311	2019	I	Active, not recruiting	Ipilimumab/Nivolumab	Talimogene Laherparepvec (T-VEC)	Breast Cancer	[352]
NCT04068181	2019	II	Active, not recruiting	Pembrolizumab	Talimogene Laherparepvec (T-VEC)	Melanoma	[353]
NCT03003676	2016	I	Completed(October 2020)	Pembrolizumab	ONCOS-102	Melanoma	[354]
NCT03004183	2016	II	Active, not recruiting	Pembrolizumab	ADV/HSV-tk	Breast/lung cancer	[355]
NCT02272855	2014	II	Completed(August 2018)	Ipilimumab	TBI-1401(HF10)	Malignant Melanoma	[356]
NCT03153085	2017	II	Completed(December 2018)	Ipilimumab	TBI-1401(HF10)	Melanoma	[357]
NCT02565992	2015	Ib	Completed(November 2019)	Pembrolizumab	CAVATAK (CVA21)	Melanoma	[358]
NCT02307149	2015	Ib	Completed(November 2019)	Ipilimumab	CAVATAK (CVA21)	Melanoma	[359]
NCT03408587	2018	1b	Completed(May 2019)	Ipilimumab	CAVATAK (CVA21)	Uveal Melanoma, Liver Metastases	[360]
NCT02824965	2016	I/II	Active, not recruiting	Pembrolizumab	CAVATAK (CVA21)	NSCLC	[361]
NCT02043665	2013	Ib	Completed(January 2020)	Pembrolizumab	CAVATAK (CVA21)	NSCLC, Bladder Cancer	[362]
NCT02630368	2015	II	Recruiting	Avelumab	Pexastimogene Devacirepvec (Pexa Vec)	Breast Cancer	[363]
NCT02977156	2017	I	Completed(June 2022)	Ipilimumab	Pexastimogene Devacirepvec (Pexa Vec)	Multiple solid tumours	[364]
NCT03071094	2017	I/IIa	Terminated	Nivolumab	Pexastimogene Devacirepvec (Pexa Vec)	HCC	[365]
NCT03294083	2017	Ib/Iia	Active, not recruiting	Cemiplimab	Pexastimogene Devacirepvec (Pexa Vec)	Renal cell carcinoma	[366]
NCT03206073	2017	I/II	Active, not recruiting	Durvalumab/Tremelimumab	Pexastimogene Devacirepvec (Pexa Vec)	Refractory colorectal cancer	[72]
NCT04849260	2021	Ib/II	Recruiting	ZKAB001	Pexastimogene Devacirepvec (Pexa Vec)	Melanoma	[367]
NCT04725331	2021	I/Iia	Recruiting	Pembrolizumab	BT-001	Multiple solid tumours	[368]
NCT05564897	2022	II	Recruiting	Camrelizumab	H101	Bladder Cancer	[369]
NCT05234905	2022		Not yet recruiting	Camrelizumab	H101	Uterine Cervical Neoplasms	[370]
NCT05303844	2022	Ib	Recruiting	Tislelizumab	H101	Refractory Malignant Ascites	[371]
NCT05303090	2022	Ib	Recruiting	Tislelizumab	H101	Pancreatic Ductal Adenocarcinoma	[372]
NCT05675462	2023	Ib	Recruiting	Tislelizumab	H101	HCC	[373]
NCT05823987	2023	II	Not yet recruiting	Tislelizumab	H101	Advanced Biliary Tract Cancer	[374]
NCT05222932	2022	I	Recruiting	Avelumab	TILT-123	Melanoma Head and Neck Squamous Cell Carcinoma	[375]
NCT05644509	2022		Not yet recruiting	Toripalimab	Revottack	Advanced Solid Tumor	[376]
NCT05271318	2022	I	Recruiting	Pembrolizumab	TILT-123	Ovarian Carcinoma	[375]
NCT02705196	2016	I/IIa	Recruiting	Atezolizumab	LOAd703	Pancreatic Cancer	[377]
NCT05070221	2021	I	Not yet recruiting	HX-008/	HSV-2 (rHSV2hGM-CSF)	Melanoma Stage IV	[378]
NCT04215146	2022	I	Recruiting	Pembrolizumab	CF33-hNIS ( HOV2)	Solid Tumor	[379]
NCT03954067	2019	I	Active, not recruiting	Pembrolizumab	ASP9801	Metastatic Cancer, Solid Tumors, Advanced Cancer	[367]
NCT03259425	2017		Terminated	Nivolumab	HF10	Melanoma	[380]
NCT04348916	2020	I	Active, not recruiting	Pembrolizumab	ONCR-177	Multiple solid tumours	[381]
NCT05733611	2023	II	Not yet recruiting	Atezolizumab	RP2/RP3	Refractory Metastatic Colorectal Cancer, Pmmr, MSS	[382]
NCT04616443	2020	Ib	Recruiting	HX008	OH2	Melanoma	[383]
NCT04386967	2018	I	Recruiting	Pembrolizumab (Keytruda)	OH2	Solid Tumor, Melanoma	[384]
NCT05162118	2021	I/II	Recruiting	Nivolumab	VG161	Advanced Pancreatic Cancer	[385]
NCT04301011	2020	I/IIa	Active, not recruiting	Pembrolizumab	TBio-6517	Multiple solid tumours	[386]
NCT02879760	2016	I/II	Completed(May 2020)	Pembrolizumab	Ad-MAGEA3/ MG1-MAGEA3	NSCLC	[387]
NCT05733598	2023	II	Not yet recruiting	Atezolizumab	RP3	HCC	[388]
NCT05743270	2023	II	Not yet recruiting	Nivolumab	RP3	Carcinoma of the Head and Neck	[389]
NCT03618953	2018	I/Ib	Terminated	Atezolizumab	Ad-E6E7/MG1-E6E7	HPV-Associated Cancers	[390]
NCT04445844	2020	II	Recruiting	Retifanlimab	Pelareorep	Breast Cancer	[391]
NCT05514990	2022	I/II	Recruiting	Pembrolizumab	Pelareorep	Plasma Cell Myelom	[392]
NCT04215146	2020	II	Active, not recruiting	Avelumab	Pelareorep	Breast Cancer Metastatic	[379]
NCT03723915	2018	II	Terminated	Pembrolizumab	Pelareorep	Pancreatic Cancer	[393]
NCT04102618	2019	I	Terminated	Atezolizumab	Pelareorep	Breast Cancer	[394]
NCT04673942	2020	I/II	Recruiting	Checkpoint Inhibitor, Immune	AdAPT-001	Solid Tumor, Neoplasms	[395]
NCT03605719	2018	I	Active, not recruiting	Nivolumab	Pelareorep	Recurrent Plasma Cell Myeloma	[396]
NCT04123470	2019	I/II	Active, not recruiting	Atezolizumab	delolimogene mupadenorepvec (LOAd703)	Malignant Melanoma	[397]
NCT02798406	2016	II	Completed(June 2021)	Pembrolizumab	DNX-2401	Brain Cancer, Glioma	[398]
NCT05084430	2021	I/II	Recruiting	Pembrolizumab	M032	Glioblastoma Multiforme, Anaplastic Astrocytoma	[399]
NCT03889275	2019	I	Completed(December 2022)	Durvalumab	MEDI5395	Advanced Solid Tumors	[400]
NCT03799744	2019	I	Active, not recruiting	Durvalumab	VCN-01	Carcinoma of Head and Neck Neoplasms	[401]
NCT03647163	2018	I/II	Recruiting	Pembrolizumab	VSV-IFNβ-NIS	Solid Tumor, Non Small Cell Lung Cancer, Neuroendocrine Carcinoma	[402]
NCT04387461	2020	II	Active, not recruiting	Pembrolizumab	CG0070	Non Muscle Invasive Bladder Cancer	[403]
NCT04735978	2021	I	Recruiting	Nivolumab	RP3	Advanced Solid Tumor	[404]
NCT04796194	2021	II	Recruiting	Pembrolizumab	LTX-315	Advanced Melanoma	[405]
NCT03767348	2018	I/II	Recruiting	Nivolumab	RP1	Multiple solid tumours	[406]
NCT04050436	2019	II	Active, not recruiting	Cemiplimab	RP1	Cutaneous Squamous Cell Carcinoma	[407]
NCT05383170	2022	Ib/Iia	Recruiting	Pembrolizumab	CyPep-1	Advanced Head and Neck Squamous Cell Carcinoma, Advanced Breast Cancer, Advanced Melanoma	[408]
NCT04260529	2020	I/Iia	Recruiting	Pembrolizumab	CyPep-1	Advanced Solid Tumor Malignancy	[408]

#### Potential challenges and future directions

Despite the groundbreaking advancements in cancer therapy brought about by ICIs, the results of the phase 3 component (ClinicalTrials.gov Identifier: NCT02263508) of the MASTERKEY-265 trial have shown that T-VEC adds pembrolizumab did not significantly improve PFS or OS to pembrolizumab monotherapy in patients with advanced melanoma [74]. One possible explanation for these results is the high proportion of patients (more than 60%) were PD-L1 positive, indicating that they had "hot" tumors at baseline. Hot tumors are characterized by an inflamed tumor microenvironment with existing immune cell infiltration. It is hypothesized that OVs may be more effective in patients with cold tumors at baseline. These observations highlight the importance of patient selection and stratification in the context of OV-ICI combination therapy. Identifying patients with cold tumors at baseline who may benefit from the addition of OVs to ICIs could improve response rates and clinical outcomes.

Furthermore, numerous preclinical studies have consistently highlighted that the timing of OV administration, when combined with ICIs, can significantly influence treatment outcomes [75]. Various dosing schedules have been explored in both laboratory models and clinical trials, revealing the complexity involved in achieving optimal therapeutic synergy [76–78]. Therefore, in order to maximize the synergistic potential of OV-ICI combination therapy, it is essential to carefully consider the optimal sequence and timing of administration. Despite extensive research, numerous fundamental challenges persist in the optimal coordination of OV and ICI administration, highlighting the need for further basic research in this area.

### ICI combined with chimeric antigen receptor (CAR) T cell

**Fig. 4:**
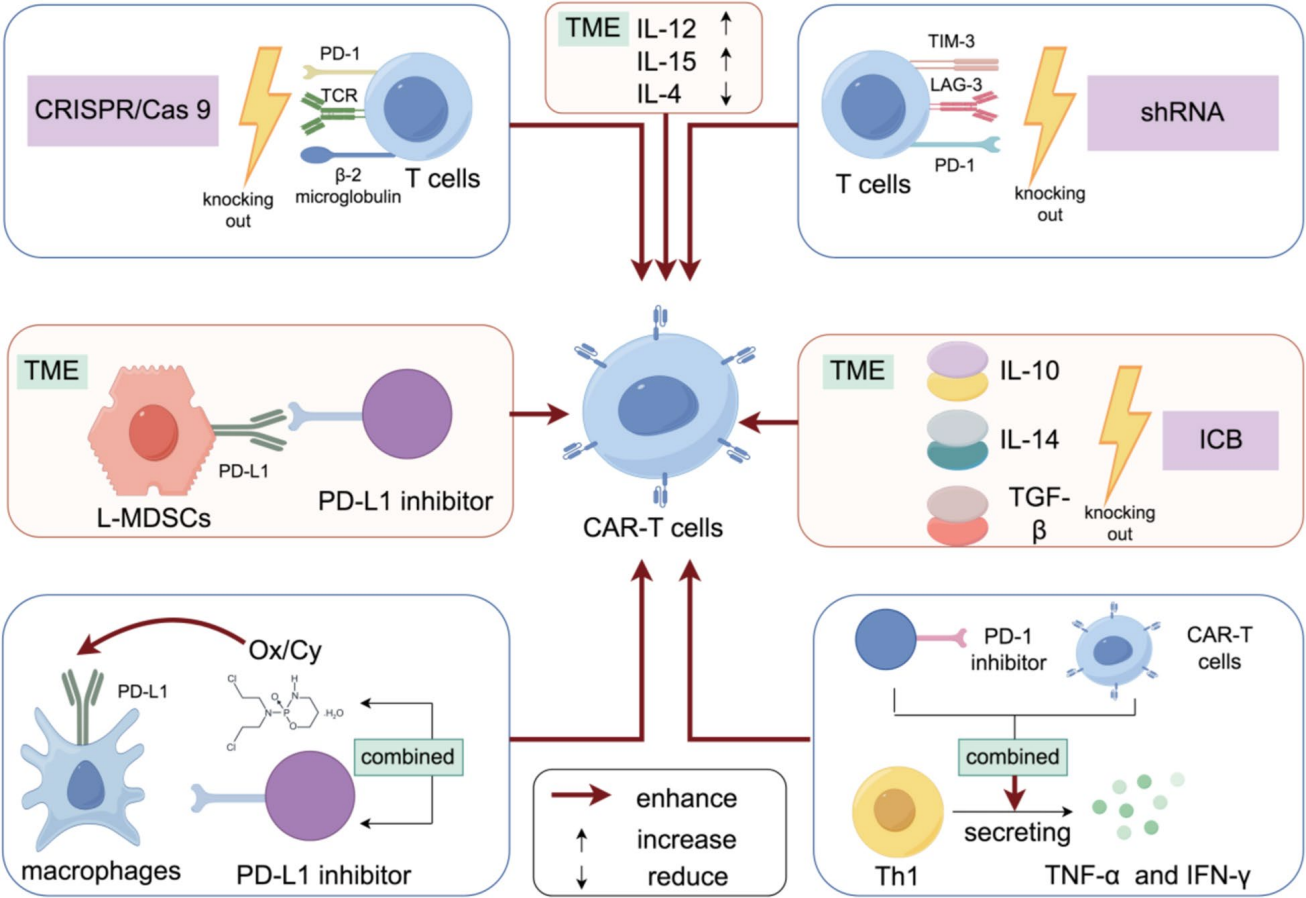
Enhancing CAR-T Cell Efficacy Through Immune Checkpoint Blockades (ICBs) and Tumor Microenvironment Modulation. Utilizing CRISPR/Cas9 technology, CAR-T cells engineered to knock out PD-1, TCR, and β2-microglobulin have been equipped with additional ICBs, which enhance cytotoxic activity and improve tumor clearance by CAR-T cells. The triple knockdown of PD-1, TIM-3, and LAG-3 by shRNAs (short hairpin RNAs) in CD39 + CAR-T cells enhances antitumor activity. The application of a PD-L1 inhibitor to counteract the effects of L-MDSCs effectively restores the antitumor efficacy of CAR-T cells, regulates antigen expression on their cell surface, and mitigates the release of multiple immunosuppressive factors. The combination of PD-L1 inhibition with Oxaliplatin/Cyclophosphamide (Ox/Cy) improves the anticancer effects of CAR-T cells by increasing the number of PD-1 + CAR-T cells and the expression level of PD-L1, thereby enhancing the tumor microenvironment (TME) through the modulation of immunostimulatory signal secretion, such as IL-12 and IL-15, and by reducing the effects of immunosuppressive cytokines like IL-4. ICBs inhibit IL-10, IL-4, and TGF-β in the TME, while the PD-1 inhibitor restores the production of TNF-α and IFN-γ, thereby facilitating anti-cancer immunity

Recent comprehensive reviews have highlighted diverse CAR-T cell combination therapies in hematologic malignancies, providing valuable insights for solid tumor applications [79]. ICI involves the use of antibodies that can block inhibitory checkpoints (like CTLA-4, PD-L1, PD-1) on immune cells, allowing these cells to remain active and attack tumor cells [80]. This therapy has demonstrated efficacy in a range of solid tumors, including lung cancer, melanoma and kidney cancer. Some of the ICIs currently approved by the FDA include Pembrolizumab, Nivolumab, and Ipilimumab [81, 82]. CAR-T cell therapy is a treatment process in which T cells are extracted from a patient's blood and genetically modified to express CARs on their surface. These CARs are engineered receptors that enable T cells to recognize and bind to specific protein markers on the surface of cancer cells, significantly enhancing their ability to target and destroy malignant cells [83]. Once modified, the engineered T cells are expanded in number and reinfused into the patient, where they actively seek out and eliminate cancerous cells. The most notable achievements of CAR-T cell therapy have been observed in hematological malignancies. The treatment has demonstrated particular efficacy in B-cell lymphomas and leukemias, where conventional treatments often prove insufficient [84, 85] (Fig. 4).

#### Synergistic mechanisms of immune modulation

##### Targeting the multiple antigens

Malignant solid tumors often exhibit antigenic heterogeneity by expressing different antigens on cell surface [86]. Meanwhile, CAR-T cells are typically engineered to focus on one antigen, which somehow limits their effectiveness against cancer cells [87]. However, combining CAR-T cell therapy with ICIs can help overcome such issue to a certain extent [88, 89].

For instance, CAR-T cells generated by knocking out PD-1, β-2 microglobulin and TCR through CRISPR/Cas9 technology that has been endowed extra Immune checkpoint blockade (ICB), displayed the enhanced cytotoxic activity and tumor clearance of CAR-T cells [90]. Additionally, a novel type of CAR-T cell, derived from hepatitis B virus (HBV) surface protein-specific chimeric antigen receptors (HBVs-CAR-T cells), has been developed. Among these, a subgroup of CD39^+^ CAR-T cells has been observed to exhibit superior anti-tumor activity [91]. Furthermore, the triple knockdown of LAG-3, TIM-3 and PD-1 by shRNAs (RNA-Short hairpin RNAs) in CD39^+^ CAR-T cells has been discovered to increase antitumor effectiveness [91]. Evidence has demonstrated that nivolumab significantly reduces PD-1 expression on the surface of GD2 CAR-T cells. The decreased PD-1 levels correlate with enhanced persistence of cytotoxic activity, specifically against PD-L1-expressing glioma cell lines U251 and U87 [92].

##### Modifying the tumor immunosuppression microenvironment

The cancer related microenvironment is a complex and evolving entity. Cancer cells can regulate antigen expression on their cell surface and release multiple immunosuppressive factors, creating an immunosuppressive microenvironment [93, 94]. Therefore, the immunosuppressive tumor microenvironment leads to inefficient infiltration of CAR-T cells into solid tumors, resulting in under-activation and depletion of CAR-T cells [37, 95]. The tumor battlefield exists within inflamed, excluded, or desert immune phenotypes, each requiring distinct therapeutic strategies [96]. This is particularly evident in hepatocellular carcinoma, where the immunosuppressive microenvironment significantly impacts progression, metastasis, and therapy outcomes [97].

Liver myeloid-derived suppressor cells has been found to exist in cancer related microenvironment, which can improve the immune escape of cancer cells, and even compromise the antitumor effects of CAR-T cells by expressing PD-L1 [98]. However, applying PD-L1 inhibitor to conquer the effect of L-MDSCs can effectively restore the anti-tumor effect of CAR-T cells in colorectal cancer liver metastases. Meanwhile, adding PD-L1 inhibitor into CAR-T cells has been found to hold the similar efficacy of combining MDSC-depleting antibodies with CAR-T cells [98]. In addition, in lung cancer cells, PD-L1 inhibition combined with Ox/Cy (Oxaliplatin/cyclophosphamide) has been reported to improve the anti-cancer effects of CAR-T cells by increasing the amount of PD-1^+^ CAR-T cells, and the expression level of PD-L1 on tumor macrophages [99]. Such approach possesses the potential to be broadly applied in making “cold” tumors more sensitive to CAR-T cells. Moreover, CAR-T cells have also been found to increase the effectiveness of PD-1 inhibitors in treating pancreatic, glioblastoma and ovarian cancer cells by improving TME. This is achieved by increasing the secretion of immunostimulatory cytokines, such as IL-12 and IL-15, while simultaneously reducing the impact of immunosuppressive cytokines, like IL-4 [100–102].

##### Potentiating CAR-T cell responses

ICIs have been found to enhance the function and persistence of CAR-T cells in cancer treatment [103]. The presence of inhibitory cytokines, such as TGF-β, IL-4 and IL-10, in the TME significantly hampers the activity of CAR-T cells [104]. Those inhibitory cytokines have been found to be suppressed by immune checkpoints blockade. By blocking these inhibitory signals, ICIs can promote the expansion and infiltration of CAR-T cells into the TME. This enhanced infiltration facilitates the recognition and destruction of cancer cells, further boosting the efficacy of CAR-T cell therapy [105]. Moreover, evidence has also reported that inhibition of PD-1 restores the effector functions of CD28^+^ CAR-T cells. Overexpression of PD-L1 on cancer cells significantly impairs CAR-T cell activity, particularly in the context of chronic antigen exposure. This PD-1/PD-L1 interaction hampers the efficacy of CAR-T cells, and the inhibition of this pathway is crucial for restoring their full anti-tumor potential, especially under conditions of sustained antigenic stimulation [106]. In addition, co-administration of pablizumab (an PD-1 inhibitor) with CAR-T cells has also been found to restore the production of interferon-gamma (IFN-γ) and tumor necrosis factor-alpha (TNF-α) which can further facilitate anti-cancer immunity [107]. For instance, PD-1 inhibition significantly enhances the functional capabilities and persistence of CAR-T cells, improving their effectiveness in treating metastatic melanoma [107]. CAR T cells with autocrine PD-L1 scFv antibody has also demonstrated enhanced anti-tumor activity in solid tumors by blocking the PD-1/PD-L1 signaling [108]. CAR-T cells engineered with an autocrine PD-L1 scFv antibody have also demonstrated enhanced anti-tumor activity in solid tumors by blocking the PD-1/PD-L1 signaling pathway. Meanwhile, the use of the autocrine PD-L1 scFv antibody has been found to significantly reduce CAR-T cell exhaustion, enhancing their long-term efficacy [108]. Optimizing CAR-T efficacy requires strategic infusion and delivery approaches [109], metabolic enhancements [110], armored CAR designs [111], and high-throughput screening methods [112], highlighting the need for a multi-faceted approach in overcoming barriers to CAR-T cell effectiveness, particularly in solid tumors. To sum up, combining ICIs and CAR-T cell therapy represents an effective and promising strategy for cancer treatment.

#### Clinical trials exploring combination therapy with ICI and CAR T cells

Current clinical evidence demonstrates significant promise in combining CAR-T cell therapy with ICIs, particularly for treating solid tumors. Multiple clinical trials have investigated this approach across various cancer types, yielding important insights into safety and efficacy [113, 114].

A phase I study (ClinicalTrials.gov Identifier: NCT01822652) examined relapsed or refractory neuroblastoma patients using three treatment approaches: CAR-T cells alone, CAR-T cells with cyclophosphamide and fludarabine, and CAR-T cells with cyclophosphamide, fludarabine, and pembrolizumab [115]. The treatments demonstrated favorable safety profiles with no dose-limiting toxicities. Notably, one patient in the pembrolizumab cohort achieved a CR, though further data collection remains necessary to fully evaluate efficacy. While this phase I study primarily established safety, it provided preliminary efficacy signals that warrant comparison with historical control data. The complete response achieved in the pembrolizumab cohort contrasts with the limited responses typically observed with either CAR-T cells or checkpoint inhibitors alone in relapsed/refractory neuroblastoma, where objective response rates with single-agent therapy rarely exceed 10–15%. This suggests potential synergistic effects of the combination approach, though larger studies are needed for definitive comparisons.

In a separate phase I trial (ClinicalTrials.gov Identifier: NCT02414269) investigating mesothelin-targeted CAR-T cell therapy for pleural malignancies, the combination with pembrolizumab showed particularly encouraging results in malignant pleural mesothelioma patients [116]. The median OS reached 23.9 months post-CAR-T cell treatment, with an impressive one-year survival rate of 83%. These promising outcomes have led to the initiation of a phase II trial to further evaluate this combination approach. The median overall survival of 23.9 months achieved with the CAR-T plus pembrolizumab combination compares favorably to historical outcomes with either modality alone. Previous studies with mesothelin-targeted CAR-T monotherapy reported median survival of approximately 10–12 months, while pembrolizumab monotherapy in similar populations yielded median survival of 8–10 months. The one-year survival rate of 83% with the combination represents an approximate 20–30% improvement over either single-agent approach, suggesting clinical benefit from the combined therapeutic strategy.

A single-arm study (ClinicalTrials.gov Identifier: NCT03726515) evaluated EGFRvIII-targeted CAR-T cells combined with pembrolizumab in newly diagnosed glioblastoma patients. The treatment demonstrated acceptable safety and tolerability, achieving a median PFSl of 5.2 months and median OS of 11.8 months [117]. Current clinical trials (ClinicalTrials.gov Identifier: NCT04003649) are exploring IL13Rα2-CAR-T cell therapy with checkpoint inhibitors like pembrolizumab or nivolumab in recurrent or refractory glioblastoma [118] (Table 2).

**Table 2:** Clinical trial of ICIs combination therapy with CAR-T cell for solid tumor Treatment

Trial Number	Launch	Phase	Study status	ICI Agent	CAR-T cell target agent	Tumor type	Rfs
NCT01822652	2013	I	Active, not recruiting	Pembrolizumab	GD2/iC9-GD2-CD28-OX40 (iC9-GD2) T cells	neuroblastoma	[409]
NCT02414269	2015	I/II	Active, not recruiting	Pembrolizumab	iCasp9M28z T cell infusions	Malignant Pleural Disease, Mesothelioma, Metastases, Lung Cancer, Breast Cancer	[348]
NCT03726515	2018	I	Completed (February 2021)	Pembrolizumab	CART-EGFRvIII T cells	glioblastoma	[410]
NCT03525782	2018	I/II	Unknown status [Previously: Recruiting]	PD-1 mAb	Anti-MUC1 CAR T Cells	Lung Neoplasm Malignant	[411]
NCT04003649	2019	I	Recruiting	Ipilimumab/Nivolumab	IL13Ra2-CAR T Cells	NSCLC	[118]
NCT03874897	2019	I	Recruiting	Toripalimab	CAR-CLDN18.2 T-Cells,	Refractory Glioblastoma	[412]
NCT04581473	2020	Ib/II	Recruiting	Anti-PD-1 antibody	CT041 autologous CAR T-cell injection	Advanced Solid Tumor	[413]
NCT04847466	2021	II	Recruiting	Pembrolizumab	PD-L1 t-haNK	Gastric Adenocarcinoma, Pancreatic Cancer	[414]
NCT04995003	2021	I	Recruiting	Pembrolizumab/nivolumab	HER2 CAR T cells	Sarcoma	[415]

#### Potential challenges and future directions

The overwhelming majority of results from the trials reviewed here have indicated that combining ICI and CAR-T cell is both feasible and safe. However, this combined approach was still under investigation in preclinical studies and early-stage clinical trials. There were also ongoing investigations into the optimal timing and sequencing of these therapies, as well as potential safety concerns related to increased immune-related side effects. The trials differ based on the agents applied, dosing strategies, and schedules for administration. Clinical trials and ongoing research aim to optimize the combination of ICIs and CAR-T cell therapy for solid cancer treatment, with the ultimate goal of improving patient outcomes. This will offer the clearest understanding of the future potential of this approach for treating solid tumors and will provide guidance on strategies to develop new methods for studying combination therapies that can deliver these breakthrough treatments to patients.

### ICI Combined with Tumor-infiltrating lymphocytes (TIL)

Lymphocytes, made up of B cells and T cells which play a crucial effective apparatuses in anti-cancer immunity, can recognize cancer cells as abnormal and penetrate into them [119–121]. Such process was defeined as TIL, TILs can be isolated, screened, and expanded in vitro, and then reinfused into patients, where they exert specific tumor-killing effects [122]. By harnessing the power of the patient's own immune system, such therapeutic approach offers a personalized and targeted strategy for combating cancers [123]. Evidence indicates that enhanced immune cell infiltration in tumors is linked to improved survival rates in patients and could be a predictor of response to immune therapies [124]. However, the application of isolated TILs is oftenly unable to generate strong effector responses by monotherapy, and this can impair its therapeutic effect [125]. To overcome such issue, ICIs have been added as a sensitizer [126].

#### Synergistic mechanisms of immune modulation

##### Enhanced tumor recognition

One of the main issues of applying TIL has been reported is that cancer cells can still express immune checkpoint molecules that dampen the T cell response, even the TIL therapy had been equipped with T cells that can recognize cancer antigens and kill cancer cells. Therefore, ICIs are introduced to block checkpoint molecules expressed by cancer cells and guarantee the efficacy of TILs [127]. As shown by evidence, the anti-cancer effects possessed by T cells can be significantly improved by adding inhibitors against LAG3, TIM3 and PD-L1, or which can positively restore responses of HCC-derived T cells to tumor antigens [128].

##### Increased T cell function and persistence

ICIs have been reported to be able to restore T cell function and prevent T cells from being exhausted or suppressed, by releasing immune checkpoints [129]. Such ability enables TILs to maintain their activity for longer periods and exert a more potent anti-tumor effect [130]. Thus, the combination of TIL therapy and ICIs can enhance the persistence and function of T cells. Moreover, CTLA-4 blockade has also been found to improve the anti-cancer effect of TILs by increasing TIL proliferation, proportion of CD8 + T cells and TIL immunoreaction, in ovarian cancer cells, compared to co-culture of IL-2 and TILs [131]. In addition, blockade of PD-1 and CTLA-4 has been found to increase the proliferation of TILs in murine models of colon cancer and ovarian cancer, which express these corresponding immune checkpoints [132]. Besides that, triple blockade of PD-1, TIM-3 and BTLA-4 has been observed to enhance the expansion and proliferation of specific CD8^+^ peripheral blood mononuclear cells (PBMCs) in melanoma [133].

##### Overcoming immunosuppressive tumor microenvironment

Tumor cells have been found to be able to generate an immunosuppressive microenvironment that hampers the function of TILs [134]. ICIs, however, can help overcome this barrier by blocking inhibitory signals and creating a more favorable immune environment for TILs to operate effectively, including the infiltration and activity of TILs [127]. For instance, cholangiocarcinoma (CCA) cells with positive PD-1 and CTLA-4, have been reported to form immunosuppressive TME by decreasing the amount of cytotoxic immune cells, increasing the amount of Tregs and making tumor-infiltrating T cells to express receptors of PD-1 and CTLA-4. However, combination therapy of PD-1 or CTLA-4 inhibitor with TIL has displayed the abilities of positively improving such immunosuppressive TME therefore improves the anti-cancer effect on CCA cells. [135].

#### Clinical trials exploring combination therapy with ICI and TIL

Recent clinical studies investigating the combination of TIL therapy and ICIs have shown encouraging therapeutic potential in several cancer types. This combined strategy seeks to boost anti-tumor immune responses by utilizing the complementary mechanisms of both treatment approaches.

A phase 1 single-arm trial (ClinicalTrials.gov Identifier: NCT03215810) investigating TIL therapy with nivolumab in patients who had previously progressed on nivolumab monotherapy yielded encouraging results. Among 13 evaluable patients, three achieved confirmed responses, while 11 demonstrated reduced tumor burden with a median best change of 35%. Notably, two patients achieved durable complete responses lasting beyond 1.5 years, indicating the potential for long-term therapeutic benefit [136]. This study demonstrated an objective response rate of 23% in patients who had previously progressed on nivolumab monotherapy, indicating the potential for TIL therapy to overcome resistance to ICI treatment alone. Notably, the two patients achieving durable complete responses had previously shown no objective response to nivolumab monotherapy, suggesting that the combination approach may provide significant clinical benefit beyond what either modality could achieve independently.

A phase II study (ClinicalTrials.gov Identifier: NCT01174121) examined short-term cultured autologous TILs in combination with pembrolizumab following lymphodepletion. One particularly notable case involved a patient receiving approximately 1.85e10 mutation-specific T cells targeting four neoantigens. This patient experienced complete regression of metastatic tumors across multiple sites, including liver, lymph nodes, and mediastinum, with the response continuing at 66 months following treatment initiation [137]. The IOV-LUN-201 phase 2 multicenter trial (ClinicalTrials.gov Identifier: NCT03419559) is actively evaluating autologous LN-145 therapy, both alone and in combination with durvalumab, in anti-PD-1/PD-L1-naïve NSCLC patients who have received prior treatment. This study will offer further insights into the therapeutic potential and safety of this combination [138]. As an ongoing study, data collection and analysis are still in progress, and the results and conclusions of the trial are yet to be published (Table 3).

**Table 3:** Clinical trial of ICIs combination therapy with TIL cell for solid tumor Treatment

Trial number	Launch	Phase	Study status	ICI agent	TIL cell target agent	Tumor type	Rfs
NCT02621021	2015	II	Recruiting	Pembrolizumab	Young TIL	Melanoma	[416]
NCT03287674	2017	I/II	Completed(June 2020)	Ipilimumab/Nivolumab	TIL infusion	Metastatic Ovarian Cancer	[417]
NCT05727904	2023	II	Recruiting	Pembrolizumab	Lifileucel(LN-144)	Metastatic Melanoma, Unresectable Melanoma, Melanoma	[418]
NCT05176470	2022	I	Recruiting	Pembrolizumab	Lifileucel(LN-144)	Locally Advanced Melanoma, Cutaneous Melanoma, Stage IV Melanoma	[419]
NCT04677361	2020	I	Withdrawn	Pembrolizumab	Marrow infiltrating lymphocytes (MILs™)	Small cell lung cancer, NSCLC	[420]
NCT03645928	2018	II	Recruiting	Ipilimumab/Nivolumab	Lifileucel(LN-144)	Metastatic Melanoma, Squamous Cell Carcinoma of the Head and Neck, NSCLC	[421]
NCT03108495	2017	II	Recruiting	Pembrolizumab	LN-145	Cervical Carcinoma	[422]
NCT02500576	2015	II	Completed(October 2022)	Pembrolizumab	TIL	Metastatic Melanoma	[423]
NCT01993719	2013	II	Completed(October 2021)	Pembrolizumab	Young TIL	Metastatic Melanoma	[424]
NCT01174121	2010	II	Recruiting	Pembrolizumab	Young TIL	Multiple solid tumours	[425]
NCT05676749	2023	I	Not yet recruiting	Pembrolizumab	C-TIL051	Metastatic Non Small Cell Lung Cancer	[426]
NCT05681780	2023	I/II	Recruiting	Nivolumab	CD40L TIL	Non Small Cell Lung Cancer	[427]
NCT03449108	2018	II	Recruiting	Ipilimumab /Nivolumab	LN-145/145-S1	Multiple solid tumours	[428]
NCT04611126	2020	I/II	Recruiting	Ipilimumab /Nivolumab	TIL	Metastatic Ovarian Cancer, Metastatic Fallopian Tube Cancer, Peritoneal Cancer	[429]
NCT03475134	2018	I	Active, not recruiting	Nivolumab	TIL	Metastatic Melanoma	[430]
NCT03526185	2018	I	Terminated	Nivolumab/Ipilimumab	TIL	Metastatic Melanoma	[431]
NCT03419559	2018	II	Withdrawn	Durvalumab	LN-145	Non Small Cell Lung Cancer	[432]
NCT03215810	2017	I	Active, not recruiting	Nivolumab	TIL	NSCLC	[433]
NCT01701674	2012	N/A	Active, not recruiting	Ipilimumab	TIL	Metastatic Melanoma	[434]

#### Potential challenges and future directions

TILs have shown significant advantages in the treatment of solid tumors. However, their production processes are indeed time-consuming, expensive, and laborious, which limits their widespread availability. The current production methods involve surgical resection to obtain tumor tissue, followed by the collection and expansion of TILs in the laboratory. As a result, they can only be developed at a limited number of leading research institutions and companies in a few countries. To overcome these barriers and improve the accessibility of TIL therapy, a key focus is finding methods to preserve TILs after extraction without extending the processing time. This involves optimizing the culture conditions and developing specialized media to keep the TILs viable and functional. Another important aspect is improving the stability of TILs during the expansion and production phases.

Identifying prognostic biomarkers is crucial for personalized treatment with TIL therapy. By understanding the biomarkers associated with specific patients, healthcare professionals can better predict the likelihood of a positive clinical response and tailor the treatment accordingly. This area of research aims to improve patient selection and optimize treatment outcomes. In addition, T cells were found to react to various tumor antigens. However, additional research is required to identify the threshold of polyclonality or the range of antigens targeted by the infused TIL repertoire that is essential for achieving a lasting clinical response.

In summary, while TIL therapy offers promising prospects for solid tumor treatment, there are a number of obstacles that need to be addressed to improve its accessibility and effectiveness. Ongoing research is focused on improving TIL production processes, sustaining TILs, identifying prognostic biomarkers, genetically modifying TILs, and understanding the optimal antigen diversity required for durable clinical responses. These efforts aim to expand the reach and enhance the efficacy of TIL therapy in the future.

### ICI combined with RNA-based cancer vaccines

**Fig. 5:**
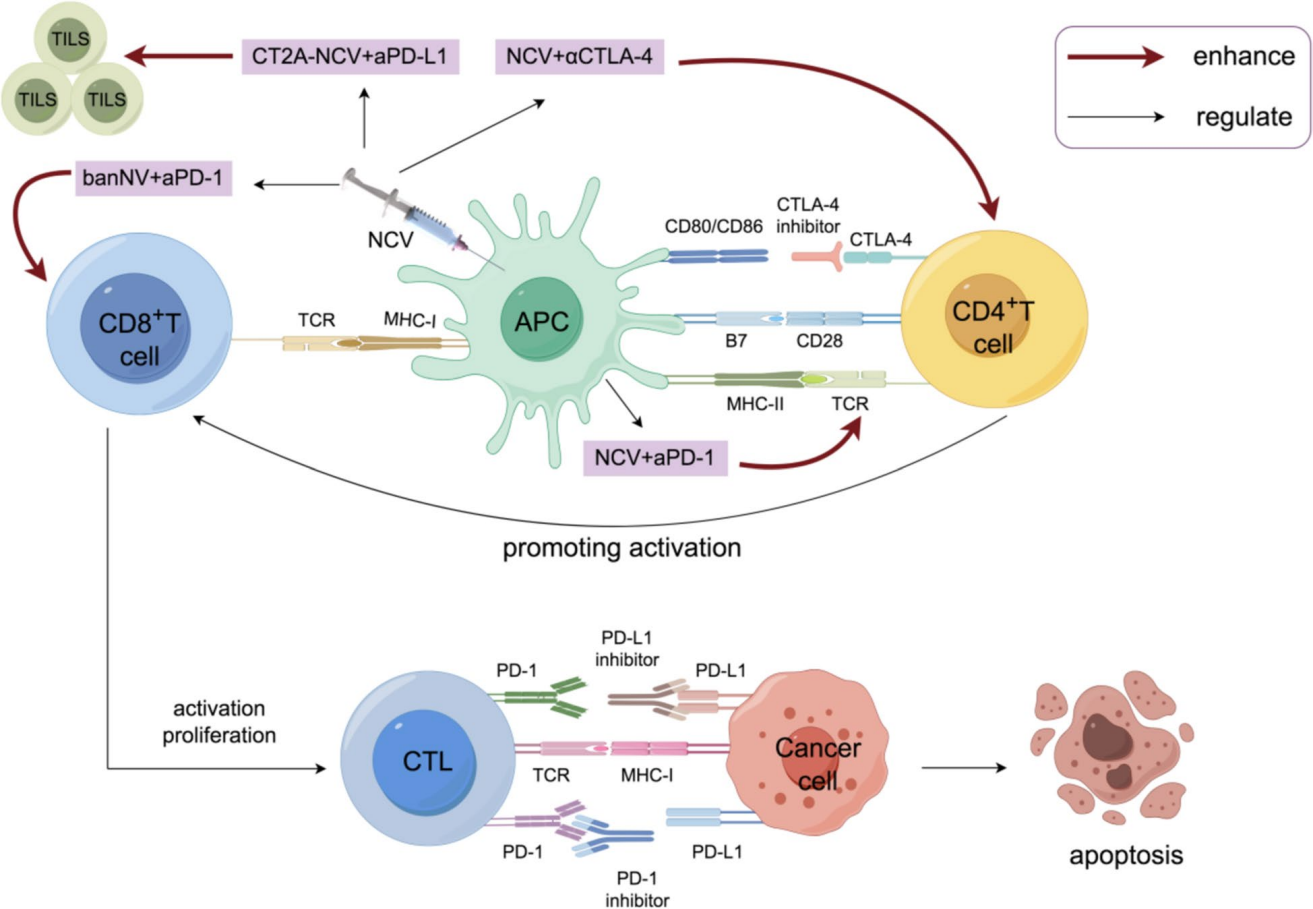
Synergistic Effects of Neoantigen Cancer Vaccines and Immune Checkpoint Blockades in Enhancing Anti-Tumor Immunity. The bi-adjuvant neoantigen nanovaccine (banNV), when combined with anti-PD-1, substantially potentiates the immunotherapeutic efficacy and can mediate cancer regression in a CD8 T cell-dependent manner. The combination therapy with αCTLA-4 and NCV enhances neoantigen-specific T cell immune responses. A CT2A-specific NVC, when combined with anti-PD-L1 blockade, was found to increase the presence of neoantigen-specific T cells within tumor-infiltrating lymphocytes (TILs). In addition, the combination of NCV and anti-PD-1 potentiates neoantigen-specific immunity, elicit robust and long-lived T cell responses with a more diversified T cell receptor (TCR) repertoire, and inhibit cancer growth

The landscape of cancer immunotherapy has been revolutionized by RNA-based vaccines, which represent an innovative therapeutic approach targeting specific genetic expressions in cancer treatment. These advanced interventions encompass multiple sophisticated mechanisms, including RNA interference through small interfering RNAs, precision genetic modifications via CRISPR-Cas9 RNA-guided systems, and therapeutic applications of messenger RNA molecules [139]. A particularly promising aspect of RNA-based cancer vaccines lies in their capacity to incorporate neoantigens. These vaccines deliver specialized messenger RNA molecules that correspond to antigens arising from tumor-specific mutations [140]. When these mRNAs enter cells, they facilitate the expression of neoantigens, thereby triggering targeted immune responses against cancer cells [141]. While RNA-based cancer vaccines have shown potential for inducing sustained immune responses across a variety of cancer types, optimizing combination therapeutic strategies is essential to improve treatment efficacy and patient outcomes [140]. ICIs have demonstrated long-lasting clinical benefits in cancer therapy, and researchers are actively investigating their combination with other therapeutic modalities to enhance their effectiveness. Among these combinations, the integration of RNA-based cancer vaccines has garnered particular interest due to their ability to further amplify immune responses and potentiate the effects of ICIs (Fig. 5) [142, 143].

#### Synergistic mechanisms of immune modulation

##### Overcome suppressive microenvironment

The tumor microenvironment presents significant obstacles to effective immune responses, including checkpoint-mediated suppression, immunosuppressive cellular networks, and inhibitory factors released during tumor cell death [144]. Recent studies have demonstrated that combining RNA-based cancer vaccines with checkpoint inhibition effectively counteracts these immunosuppressive mechanisms. A particularly compelling example comes from research using a bi-adjuvant neoantigen nanovaccine in conjunction with PD-1 blockade, which significantly enhanced therapeutic efficacy and extended survival in colon cancer models [145]. The importance of this combination approach was further validated in MC38 colon carcinoma studies, where neoantigen vaccination combined with anti-PD-1 therapy achieved tumor regression through CD8 T cell-mediated mechanisms [146]. Notably, anti-PD-1 monotherapy proved insufficient for tumor clearance, highlighting the synergistic benefit of the combination strategy.

##### Priming of T cell responses

The addition of RNA-based cancer vaccines to checkpoint inhibition therapy demonstrates remarkable effects on T cell responses, both in "hot" and "cold" tumors. In hot tumors, this combination expands the cytotoxic T cell repertoire, while in cold tumors, it helps establish an immunologically active environment. These effects substantially broaden the population of patients who might benefit from immunotherapy [142]. Studies across multiple cancer models have provided strong evidence for this enhanced T cell response. In colon cancer models, combining neoantigen vaccination with CTLA-4 blockade led to improved neoantigen-specific T cell responses and complete tumor regression [147]. Similar benefits were observed in glioblastoma models, where the combination of CT2A-specific RNA-based cancer vaccines with anti–PD-L1 blockade resulted in an increase in tumor-infiltrating neoantigen-specific T cells and improved survival outcomes [148]. Particularly noteworthy are the findings from colon cancer lung metastasis models, where concurrent RNA-based cancer vaccines and anti-PD1 treatment generated robust, sustained T cell responses characterized by a more diverse T cell receptor repertoire [149]. These findings collectively suggest that combining ICIs with RNA-based vaccines therapy creates a more potent anti-cancer response by enhancing tumor-reactive T cell populations. This approach represents a significant advancement in cancer immunotherapy strategy, offering new possibilities for improving patient outcomes.

#### Clinical trials exploring combination therapy with ICI and RNA-based cancer vaccines

The first open-label trial (ClinicalTrials.gov Identifier: NCT02897765) of NEO-PV-01 (a pioneering personalized neoantigen vaccine) combined with PD-1 blockade established both feasibility and safety in metastatic cancer patients [150]. The trial revealed notable survival outcomes across different cancer types. Melanoma patients showed particularly impressive results, with a median PFS of 23.5 months. NSCLC and bladder cancer cohorts demonstrated median PFS of 8.5 and 5.8 months, respectively. One-year OS rates were remarkably high, reaching 96% in melanoma patients, 83% in NSCLC patients, and 67% in bladder cancer patients. The median PFS of 23.5 months observed in melanoma patients receiving the combination of NEO-PV-01 and PD-1 blockade compares favorably to historical data with anti-PD-1 monotherapy, which typically ranges from 5.1–8.2 months in similar patient populations. Similarly, the one-year OS rates of 96% in melanoma, 83% in NSCLC, and 67% in bladder cancer represent approximately 15–25% improvements over expected survival with checkpoint inhibitor monotherapy. These comparative outcomes, while from a single-arm study rather than a randomized trial, suggest potential additive or synergistic effects from combining personalized neoantigen vaccines with checkpoint inhibition.

The RO7198457 trial (ClinicalTrials.gov Identifier: NCT03289962) introduced a novel RNA-Lipoplex iNeST approach, combining this systemically administered vaccine with atezolizumab [151]. Among 108 evaluated patients, the treatment achieved an 8% objective response rate, including one CR, while 49% of patients maintained SD. Among the 108 evaluable patients, the 8% objective response rate with combination therapy was modest but included responses in tumor types typically resistant to checkpoint inhibitor monotherapy. Notably, in a subset analysis of patients with minimal prior therapy, the response rate increased to 14%, exceeding the expected 5–10% response rate with atezolizumab alone in comparable mixed solid tumor populations. The high disease control rate (57%) further suggests potential benefit from the combination approach compared to historical expectations with either modality alone.

Further advances in RNA vaccine technology emerged through the mRNA-4157 study (ClinicalTrials.gov Identifier: NCT03313778). This lipid-encapsulated, algorithm-driven vaccine demonstrated significant efficacy when combined with pembrolizumab. The treatment yielded particularly impressive results in head and neck squamous cell carcinoma, achieving a 50% response rate and median PFS of 9.8 months. These outcomes substantially exceeded historical results from pembrolizumab monotherapy[152]. These clinical trials collectively demonstrate the potential of combining neoantigen vaccines with checkpoint inhibition, particularly in achieving durable responses across multiple cancer types. The field continues to evolve, with ongoing studies (ClinicalTrials.gov Identifier: NCT03422094) exploring optimal combinations for specific cancer types, including glioblastoma [153] (Table 4).

**Table 4:** Clinical trial of ICIs combination therapy with NCVs for solid tumor Treatment

Trial number	Launch	Phase	Study status	ICI agent	Cancer vaccine agent	Tumor type	Rfs
NCT05153304	2021	I/II	Not yet recruiting	Nivolumab	Personalized vaccine	Cancer of Gastrointestinal Tract	[435]
NCT05528952	2022	II	Recruiting	Atezolizumab	CD4 Th1-inducer Cancer Vaccine (UCPVax)	HCC	[436]
NCT05456165	2022	II	Terminated	Atezolizumab/Ipilimumab	Individualized neoantigen vaccine	Colonic Neoplasms Colorectal Neoplasms	[437]
NCT03361852	2017	II	Recruiting	Pembrolizumab	Personalized neoantigen cancer vaccine	Follicular Lymphoma	[438]
NCT05141721	2021	II/III	Recruiting	Atezolizumab/Ipilimumab	Neoantigen vaccine(GRT-C901/GRT-R902)	Colorectal Neoplasms	[439]
NCT05075122	2021	II	Recruiting	Pembrolizumab	UV1 vaccination	Head and Neck Squamous Cell Carcinoma	[440]
NCT04418219	2020	I/II	Withdrawn	Pembrolizumab	Breast Cancer Vaccine (SV-BR-1-GM)	Breast Cancer	[441]
NCT04024878	2019	I/II	Recruiting	Nivolumab	Personalized NeoAntigen Cancer Vaccine (NeoVax)	Ovarian Cancer	[442]
NCT04263051	2020	II	Recruiting	Nivolumab	CD4 Th1-inducer Cancer Vaccine (UCPVax)	Advanced NSCLC	[436]
NCT04369937	2020	II	Recruiting	Pembrolizumab	HPV-16 E6/E7 specific therapeutic vaccination (ISA101b)	HPV-Related Squamous Cell Carcinoma, Head and Neck Squamous Cell Carcinoma	[443]
NCT04382664	2020	II	Recruiting	Ipilimumab/Nivolumab	cancer vaccine (UV1)	Malignant Melanoma	[444]
NCT03946358	2019	II	Active, not recruiting	Atezolizumab	CD4 Th1-inducer Cancer Vaccine (UCPVax)	Squamous Cell Carcinoma of the Head and Neck, Anal Canal Cancer Cervical Cancer	[445]
NCT04161755	2019	I	Active, not recruiting	Atezolizumab	personalized cancer vaccine (PCV)	Pancreatic Cancer	[446]
NCT03970746	2019	I/II	Recruiting	Pembrolizumab	PDC*lung01	Non Small Cell Lung Cancer	[447]
NCT04040231	2019	I	Active, not recruiting	Nivolumab	Galinpepimut-S	Mesothelioma, Pleural Mesothelioma Wilms Tumor	[448]
NCT03953235	2019	I/II	Active, not recruiting	Nivolumab/Ipilimumab	Neoantigen Cancer Vaccine	Solid Tumor	[448]
NCT03897881	2019	II	Recruiting	Pembrolizumab	Pembrolizumab	Melanoma	[449]
NCT03359239	2017	I	Completed(December 2017)	Atezolizumab	PGV001	Urothelial/Bladder Cancer, Nos	[450]
NCT04161755	2019	N/A	Active, not recruiting	Atezolizumab	Personalized Tumor Vaccines (PCVs)	Pancreatic Cancer	[451]

#### Potential challenges and future directions

Neoantigens are generated from tumor-specific mutations and vary widely across different tumors and patients. This high degree of heterogeneity can lead to a suboptimal response to both RNA-based cancer vaccines and ICIs. To address this challenge, designing a neoantigen vaccine with multiple targets, which takes into account the tumor’s heterogeneity, may help overcome intra-tumor variability and its evolving alterations. In addition, the identification of neoantigens remains a significant hurdle. It requires extensive tumor tissue samples, and the yield of viable epitopes or neoantigens is often minimal, making it difficult to overcome with current technical capabilities. These challenges have substantially hindered the clinical progress of neoantigen vaccines. Therefore, there is an urgent need to develop more accurate neoantigen prediction algorithms and more efficient manufacturing technologies that can reduce costs and shorten development timelines, thereby accelerating the clinical application of neoantigen-based therapies.

### CAR-T cell combined with OVs

Recent research has illuminated the potential synergistic benefits of combining OVs with CAR-T cell therapy for treating solid tumors. This therapeutic approach leverages multiple complementary mechanisms to enhance treatment efficacy The combination functions through several key pathways. First, OVs directly lyse cancer cells, releasing tumor-associated antigens. This continuous release of antigens helps prevent antigen loss, a common challenge in CAR-T cell therapy. Second, OVs work to reverse the immunosuppressive nature of the tumor microenvironment, thereby supporting improved persistence of CAR-T cells within the tumor site. Third, OVs can be engineered to express therapeutic molecules such as chemokines, further enhancing their therapeutic potential. This combination strategy specifically addresses several traditional barriers that have limited CAR-T cell efficacy in solid tumors. The ability to modify OVs provides multiple opportunities to overcome these obstacles. This combination exemplifies enhanced cellular therapy approaches that are revolutionizing adoptive immunotherapy [154].

#### Synergistic mechanisms of immune modulation

##### Increase the efficiency of CAR-T cell trafficking and persistence within cancer

The immunosuppressive TME is primarily manifested through the deficiency of pro-T cell cytokines, which enables tumors to circumvent cytotoxic T cell responses [155]. In neuroblastoma xenograft models, engineered OVs c expressing RANTES and IL-15 exhibited potent synergy with GD2-CAR-T cells. These viral vectors enhanced both CAR-T cell infiltration into tumor sites and augmented tumor cell susceptibility to T cell-mediated cytotoxicity [156]. Comparable results were observed when CAR-T cells were used in combination with oncolytic vaccinia virus expressing CXCL11, a potent chemokine that draws T cells to tumor locations. Furthermore, direct delivery of CXCL11 to the tumor site via the oncolytic vaccinia virus was found to be more efficient in inducing cancer cell killing in vivo than the secretion of CXCL11 by CAR-T cells themselves [157].

This suggests that the direct delivery of CXCL11 by the oncolytic virus enhances immune cell recruitment and improves the overall therapeutic effect. This combination approach has shown particular promise in challenging cancer types, such as pancreatic ductal adenocarcinoma. OVs modified to express TNF-α and IL-2 worked synergistically with CAR-T cells to overcome the characteristically immunosuppressive microenvironment. This combination successfully promoted M1 macrophage polarization and dendritic cell (DC) maturation, effectively preventing metastasis despite the aggressive nature of the disease [158]. Further innovations include the development of transforming growth factor β signaling-targeted oncolytic adenovirus (rAd.sT), which enhanced the effectiveness of mesothelin-targeted CAR-T cells in triple-negative breast cancer models [159]. Additionally, IL-7-expressing OVs demonstrated significant improvements in B7H3-CAR-T cell proliferation and persistence in glioblastoma models, while reducing T cell apoptosis [160].

##### Overcome the attenuation of antigen escape induced by T-cell immunotherapy

Cytotoxic T-cells show significant potential in targeting cancer cells that express specific antigens. However, a major challenge arises when initial T-cell interventions eliminate antigen-expressing cancer cells, leaving behind resistant tumor populations that are no longer targeted. This selective pressure can lead to tumor immune evasion and subsequent disease progression [161]. An innovative strategy involves the use of an engineered OVs armed with an epidermal growth factor receptor-targeting bispecific T-cell engager (OAd-BiTE). This approach significantly enhances CAR T-cell activation and proliferation, while also boosting cytokine production, improving overall cytotoxicity, and maintaining a favorable safety profile compared to traditional EGFR-targeted CAR T-cell therapies [162]. In another study, researchers developed an oncolytic vaccinia virus carrying a CD19-targeted protein (OV19t) and investigated its potential in combination with CD19-CAR T-cells using human solid tumor xenograft models. The results revealed a multifaceted mechanism of action, which facilitated the enhanced infiltration of endogenous T-cells into the tumor sites, improved CAR T-cell recruitment within the tumor microenvironment, and enabled CD19-CAR T-cells to trigger the release of intact virus particles from dying cancer cells [163]. This combination therapy demonstrated remarkable efficacy in cancer regression, offering a sophisticated approach to overcoming the traditional limitations of T-cell immunotherapy and expanding the therapeutic potential of OVs and engineered immune cells.

#### Clinical trials exploring combination therapy with CAR-T cell and OVs

Despite the promising preclinical results, only a few clinical trials investigating the combination of CAR-T cells and OVs are currently underway, underscoring the challenges of translating this approach to clinical practice. One of the first-in-human phase I clinical trials (ClinicalTrials.gov Identifier: NCT03740256) is exploring the potential of this combination therapy for treating advanced HER2-positive solid tumors. In this trial, a binary OVs is being used in combination with autologous CAR-T cells specifically targeting HER2, representing a novel approach to treating this aggressive cancer type [146].

Another phase I clinical trial (ClinicalTrials.gov Identifier: NCT05057715) is evaluating the safety and feasibility of combining huCART-meso (human CAR-modified T cells) with VCN-01, an OVs that replicates in cancer cells with RB1 pathway dysfunction and expresses hyaluronidase. The trial employs a 3 + 3 dose escalation design to optimize the combination therapy [147]. Although these trials represent early-stage investigation of this promising treatment approach, additional studies are needed to assess the efficacy and safety of combining CAR-T cells with OVs in human patients (Table 5). While these early-phase trials focus primarily on safety and feasibility, they include important secondary endpoints comparing efficacy outcomes to historical data with either CAR-T or OV monotherapy. In particular, the NCT05057715 trial incorporates a study design that allows for comparison of response rates and survival outcomes with published benchmarks for huCART-meso or VCN-01 when used as single agents. Preliminary data from these trials will be crucial in determining whether the promising synergistic effects observed in preclinical models translate to meaningful clinical benefits compared to monotherapy approaches.

**Table 5:** Clinical trial of CAR-T cell combination therapy with OVs for solid tumor Treatment

Trial Number	Launch	Phase	Study status	CAR-T cell target agent	OVs agent	Tumor type	Rfs
NCT03740256	2018	N/A	Recruiting	HER2 specific CAR T cells	CAdVEC	Solid Tumor	[409]
NCT05057715	2021	I	Recruiting	huCART-meso Cells	VCN-01	Pancreatic Cancer Serous Ovarian Cancer	[413]

#### Potential challenges and future directions

The combination of CAR-T cells ad OVs and holds significant promise for cancer treatment, offering a multifaceted approach to overcome the limitations of traditional therapies. However, several challenges need to be addressed for successful clinical implementation of this combined therapy. One of the most critical concerns is the host's immune response to the viral component of the therapy. Neutralizing antibodies and cytokine secretion, particularly interferons (IFNs), can trigger a robust anti-viral immune response, leading to viral clearance and reduced viral replication. This immune response may diminish the effectiveness of the oncolytic virus and reduce its potential to enhance CAR-T cell activity in the tumor microenvironment [164]. Therefore, strategies to mitigate or manage these immune responses are essential for maximizing therapeutic efficacy.

In addition to viral immune responses, the safety profile of CAR-T cells requires rigorous evaluation, whether used alone or combined with OVs. While showing promising efficacy across various cancers, CAR-T cell therapy can cause serious adverse effects, including cytokine release syndrome and neurotoxicity, which demand careful management, particularly in combination treatments [165]. Maintaining a delicate balance between immune activation and immune-related adverse effects is crucial for optimizing the safety and efficacy of this approach. Another challenge stems from the reliance of many preclinical studies on immunodeficient mouse models, which do not fully replicate the complexities of the human immune system. To better understand how host immune responses, including anti-viral immunity, interact with CAR-T cells and OVs, further research using immunocompetent models, such as humanized mice, is necessary. These models can offer more relevant insights into how the combination therapy functions in a fully intact immune environment, helping to bridge the gap between preclinical findings and clinical applications. Addressing these challenges through improved understanding of the immune dynamics involved, as well as developing strategies to optimize viral delivery and enhance CAR-T cell persistence, will be critical in advancing the safe and effective use of OV-CAR-T cell combination therapies in clinical settings.

### CAR-T cell combined with RNA-based cancer vaccines

**Fig. 6:**
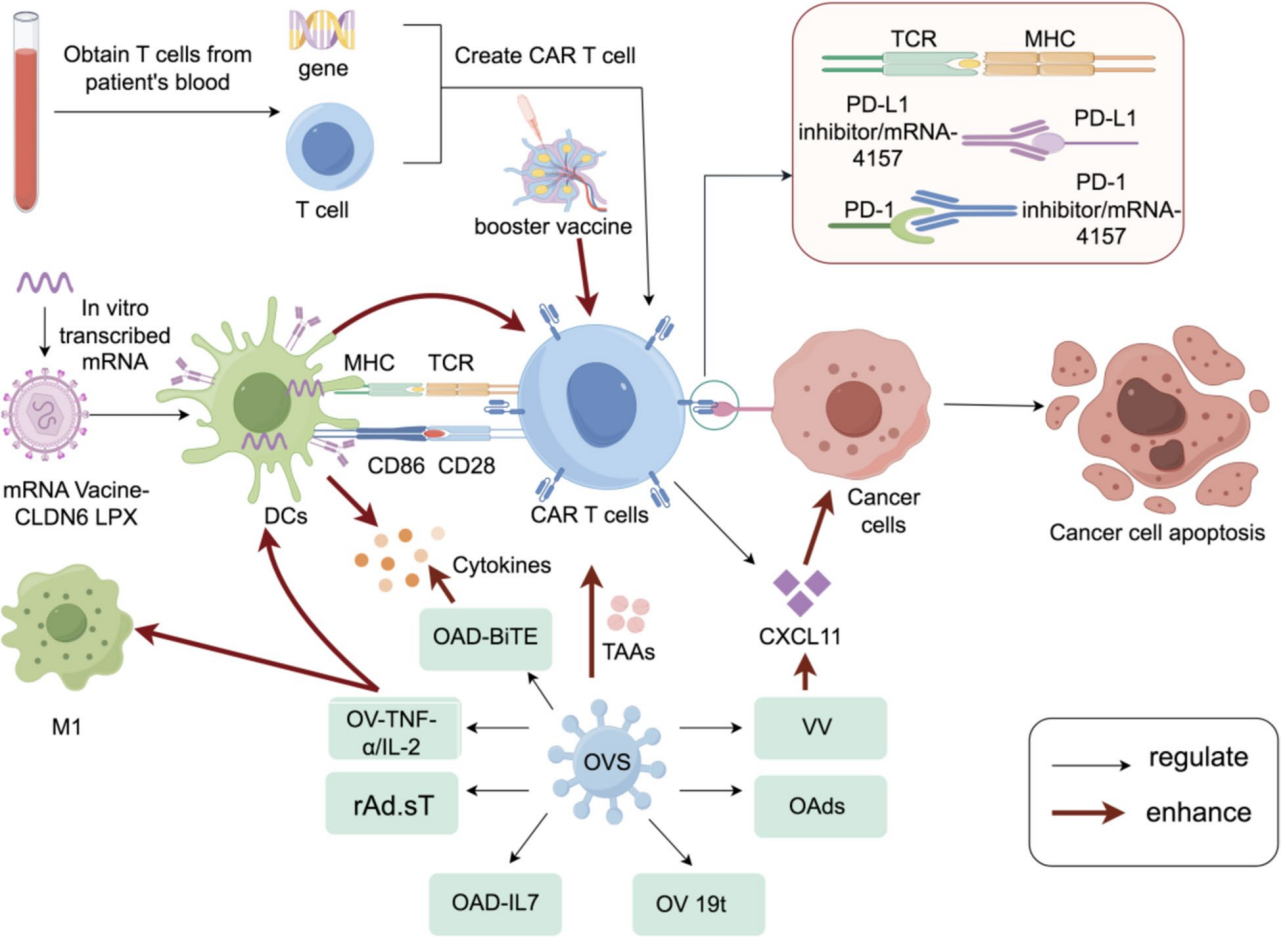
Enhancing CAR-T Cell Therapy Through mRNA Vaccines, Oncolytic Viruses, and Immune Modulation Strategies. CAR-T cell therapy is a novel form of immunotherapy that uses genetic engineering to modify T cells for targeted antigen recognition and precise activation. Combining CAR-T cell therapy with mRNA cancer vaccines can enhance the therapeutic efficacy of CAR-T cells and address the challenges associated with CAR-T cell therapy in solid cancers, such as mRNA-4157 and the vaccine encoding mRNA for CLDN6. Moreover, various strategies can be employed using oncolytic viruses (OVs) to overcome the barriers in CAR-T cell therapy for solid cancers. These strategies can help spread tumor-associated antigens (TAAs) through the direct lytic effect on cancer cells, thereby preventing antigen loss and reversing tumor-mediated immunosuppression. Examples include oncolytic adenovirus (OAds), oncolytic vaccinia virus (VV) expressing CXCL11, rAd.sT, IL7-expressing oncolytic virus, and oncolytic vaccinia virus carrying CD19t (OV19t). Additionally, a booster vaccine can activate CAR-T cells, allowing the immune system to recognize other tumor antigens and generate a “memory” T cell population capable of targeting these antigens. OVs expressing TNF-α and IL-2 have a robust anti-tumor effect, exerted by promoting the M1 polarization of macrophages and the maturation of dendritic cells (DCs). The oncolytic adenovirus armed with an EGFR-targeting bispecific T-cell engager (OAd-BiTE) can increase cytokine production and cytotoxicity, thereby improving the efficacy of CAR-T cell therapy

CAR-T cell therapy involves engineering T cells to express chimeric antigen receptors that specifically target cancer antigens [166]. CAR-T cell therapy involves engineering T cells to express chimeric antigen receptors that specifically target cancer antigens [167]. These modified T cells, when introduced into patients, can proliferate and maintain a sustained anti-tumor response [168, 169]. While this approach has shown remarkable success in the treatment of hematological malignancies, its efficacy in solid tumors remains constrained [170]. To enhance the therapeutic efficacy of CAR-T cells and address the challenges associated with CAR-T cell therapy in solid cancers, a promising strategy is to integrate RNA-based cancer vaccines therapy with CAR-T cell therapy. In recent years, there has been notable progress in the development of RNA-based vaccine platforms, which have been extensively investigated for their immunogenicity and effectiveness [171]. A diverse range of RNA-based cancer vaccines is currently in development, each featuring distinct mechanisms of action along with unique advantages and disadvantages [172]. Several preclinical studies and clinical trials have investigated the synergy between armed RNA-based vaccine and CAR-T cell therapy, shedding light on their biological mechanisms and effects. Especially, Recent advancements in cancer research have highlighted the success of neoantigen mRNA vaccines in enhancing the performance of ICIs for treating solid tumors, a development showcased in the KEYNOTE-942 clinical trial. This phase II study, also referred to as the mRNA-4157-P201 trial, investigated the effectiveness and safety of a bespoke cancer vaccine, mRNA-4157, in conjunction with pembrolizumab, a type of PD-1 inhibitor. The focus was on treating patients with head and neck squamous cell carcinoma (HNSCC), particularly those in the advanced or metastatic stages who had not received prior systemic treatments. The treatment involved administering the mRNA-4157 vaccine, which is tailored to each patient's tumor cells, thereby stimulating the immune system's attack on these cells. Pembrolizumab was used to further empower the immune system by lifting barriers that hinder its fight against cancer cells. The trial yielded optimistic outcomes, with a disease control rate of 67% and an OS rate of 33%. Additionally, the combined treatment was generally well accepted by patients, showing no significant new safety concerns [173]. Thus, integrated approach of CAR-T cells and RNA-based cancer vaccine**s** may further harness the therapeutic effect of their monotherapy (Fig. 6).

#### Synergistic mechanisms of immune modulation

##### RNA-based cancer vaccines promote the proliferation and killing efficacy of CAR-T cells and prolong the maintenance of CAR-T cells' effector functions

RNA-based cancer vaccines, particularly therapeutic ones, primarily function by triggering T-cell immune responses [174]. When combined with CAR-T cell therapy, they enhance CAR-T cell proliferation and cytotoxic efficiency. In a study, it was discovered that the combination of CLDN-CAR-T cells with a vaccine encoding mRNA for CLDN6 had a synergistic effect. This combination promoted the expansion of CAR-T cells and extended their persistence in the body, leading to enhanced cancer-killing capabilities [175]. In their in vitro experiments, they established that liposomal antigen-encoding RNA encoding CLDN6 (CLDN6-LPX) could be efficiently taken up by DCs and subsequently expressed on the surface of these DCs. This process induced cytokine secretion and proliferation of CLDN6 CAR-T cells. Furthermore, in vivo experiments involving the tail vein injection of CLDN6 LPX also facilitated the proliferation of CLDN6-CAR-T cells.

The researchers subsequently conducted a series of in vivo experiments that demonstrated the effectiveness of a low dose of CLDN6-LPX in inducing substantial expansions of CLDN6-CAR-T cells. These expanded cells exhibited full functionality, including robust cytokine secretion and potent cell-killing capabilities. Finally, by creating mouse or human tumor models in mice, the researchers confirmed that CLDN6 LPX could significantly enhance the therapeutic impact of CAR T cells on solid tumors. Notably, CLDN6 LPX allowed for a reduction in the required dose of CAR T cell therapy, preserved the longevity of CAR T cells while minimizing toxicity, and extended the treatment's effectiveness.

##### RNA-based cancer vaccines promote memory formation of CAR-T cells and overcome limitations of the suppressive immune microenvironment

One of the challenges in using CAR-T therapies against solid tumors is their limited efficacy, primarily due to the immunosuppressive environment typically created by tumors, which can render T cells ineffective before they reach their intended targets. To address this issue, a research team turned their focus to leveraging the abundance of immune cells in lymph nodes. They developed a strategy involving the delivery of a vaccine directly to the lymph nodes to activate CAR-T cells at that location [176]. In a mouse model experiment, CAR-T cells administered without the vaccine were barely detectable in the bloodstream. However, when a booster vaccine was administered the day following T-cell infusion, with another booster a week later, a remarkable transformation occurred. CAR-T cell populations increased significantly, eventually constituting 65% of the total T-cell population after two weeks of treatment. This remarkable expansion of CAR-T cell populations led to the complete eradication of glioblastomas, mammary tumors, and melanomas in many of the treated mice. In contrast, without the vaccination, CAR-T cells had no impact on tumor growth. With the vaccination protocol in place, CAR-T cells successfully eliminated tumors in 60% of the mice. Notably, around 75 days post-initial treatment, the researchers re-injected the same tumor cells that had formed the original tumors. Remarkably, the immune systems of the mice effectively cleared these tumors. Approximately 50 days later, slightly different tumor cells, lacking the antigens targeted by the initial CAR-T cells, were introduced, and yet again, the mice were able to eliminate these tumor cells [176]. These findings suggest that once CAR-T cells initiated the destruction of the tumors, the immune system recognized other tumor antigens and generated a "memory" T cell population capable of targeting these antigens as well.

#### Clinical trials exploring combination therapy with CAR-T cell and mRNA cancer vaccines

Currently, there is only one clinical trial exploring the potential of combining CAR-T cell therapy with RNA-based vaccines for cancer treatment. This Phase I/IIa study (ClinicalTrials.gov Identifier: NCT04503278) is the first of its kind in humans, open-label, multicenter, and dose-escalating. The primary objective of the trial is to assess the safety and preliminary efficacy of CLDN6 CAR-T cells, both as a standalone treatment and in combination with CLDN6 RNA-LPX, in patients with advanced solid tumors expressing CLDN6 [177]. The trial initially used CLDN6 RNA-LPX, a liposomal RNA formulation designed to enhance the immune response. In subsequent phases, the study will investigate an alternative RNA formulation, CLDN6 modRNA-LPX, once the Recommended Phase 2 Dose (RP2D) for CLDN6 CAR-T cells, either alone or with CLDN6 RNA-LPX, has been determined. A total of 16 patients with recurrent or refractory CLDN6-positive advanced solid cancers, including testicular and ovarian cancers, were enrolled. At the first efficacy assessment, six weeks after the infusion, 14 patients were evaluable for response. Among these patients, 6 achieved partial remission, translating to an objective remission rate of approximately 43%. Five patients showed SD with shrinkage of the target lesions, one remained unchanged, and two experienced disease progression. Notably, of the patients who attained PR, four received CAR-T cells alone, while two received the CAR-T-CARVac combination, resulting in an impressive disease control rate of 86%. In this first-in-human study, the objective remission rate of approximately 43% among evaluable patients represents a promising efficacy signal. When stratified by treatment approach, the partial remission rate was 36% (4/11) in patients receiving CAR-T cells alone versus 67% (2/3) in those receiving the CAR-T-CARVac combination. While the small sample size precludes definitive conclusions, this preliminary data suggests the potential for enhanced therapeutic efficacy with the combination strategy. The observation that disease control rates reached 86% with favorable safety profiles warrants further investigation in larger, controlled studies specifically designed to detect differences in outcomes between monotherapy and combination approaches.

These preliminary results suggest that CLDN6 CAR-T cell therapy combination with CAR-Vac, is not only safe but also holds considerable therapeutic promise for patients with CLDN6-positive cancers (Table 6).

**Table 6:** Clinical trial of CAR-T cell combination therapy with mRNA cancer vaccines for solid tumor Treatment

Trial number	Launch	Phase	Study status	CAR-T cell target agent	Cancer vaccine agent	Tumor type	Rfs
NCT04503278	2020	I	Recruiting	CLDN6 CAR-T	CLDN6 RNA-LPX	CLDN6-positive relapsed or refractory advanced solid tumors	[452]

#### Potential challenges and future directions

Compared to traditional vaccines, mRNA vaccine production offers notable advantages in terms of manufacturing flexibility and rapid scalability, particularly in response to emerging infectious diseases [178]. However, potential unintended immune responses and off-target effects remain concerns. Despite extensive preclinical and clinical testing for safety assurance, continuous monitoring is crucial for detecting long-term effects and rare adverse events [179]. Furthermore, tumor subpopulation heterogeneity may increase recurrence risk under combination therapy pressure. Regarding tumor microenvironment characteristics, post-combination therapy infiltration of immunosuppressive cells (such as Tregs and tumor-associated macrophages) and immunosuppressive cytokine secretion may limit therapeutic efficacy. Currently, large-scale two-arm clinical trials comparing combination therapy outcomes are lacking. Based on completed clinical trial data, cutting-edge research focuses on designing and combining novel antigens, implementing regional CAR-T cell delivery, activating bystander immune cells and tumor microenvironment, developing multifunctional CAR-T cells through mRNA transfection, and integrating promising approaches to overcome solid tumor treatment challenges.

### OVs combined with TIL

**Fig. 7:**
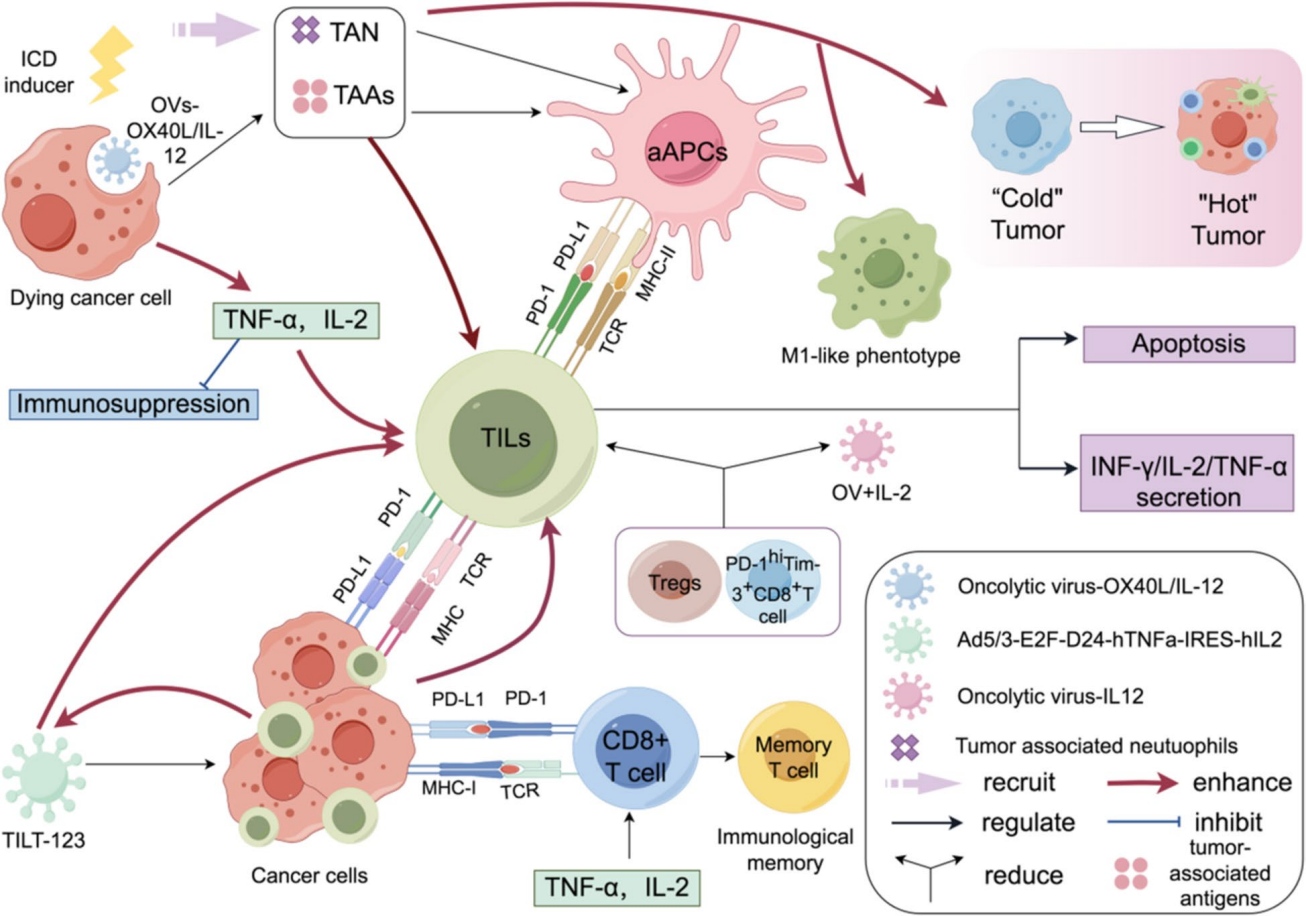
Synergistic Enhancement of Tumor-Infiltrating Lymphocyte Therapy Through Oncolytic Virus-Induced Immunogenic Modulation and Activation. Tumor infiltrating lymphocytes can identify and kill tumor cells, producing cytokines such as interleukin-2 (IL-2) and interferon-gamma (IFN-γ), which play an important role in their activation and proliferation. Tumor necrosis factor-alpha (TNF-α) can re-engage in inducing apoptosis of tumor cells and recruit other immune cells to the tumor site. The induction of Immunogenic Cell Death (ICD) by oncolytic viruses (OVs), which released Tumor-Associated Antigens (TAAs) and recruited Tumor-Associated Neutrophils (TANs), transformed 'cold' tumors into 'hot' tumors with increased TIL accumulation, reprogrammed macrophages toward a more M1-like phenotype. The herpes simplex virus 1 (HSV-1)-based oncolytic virus encoding OX40L and IL-12 (OV-OX40L/IL12) converted tumor cells into artificial antigen-presenting cells (aAPCs), providing local signals and enhancing T cell activation and killing in coculture with TILs. The oncolytic adenovirus coding tumor necrosis factor alpha (TNF-α) and interleukin-2 (IL-2) (Ad5/3-E2F-D24-hTNFa-IRES-hIL2; TILT-123) recruited more TILs, enhanced cytotoxicity, and held promise for promoting substantial immunological activation of locoregional TILs and counteracted immunosuppression. The combination of OV and TIL therapy using an interleukin-2 (IL-2)-armed oncolytic poxvirus, which leads to a lower percentage of exhausted PD-1hi Tim-3 + CD8 + T cells and Regulatory T cells (Tregs)

Autologous TIL immunotherapy, an adoptive cell transfer approach, has emerged as an effective strategy for advanced solid tumor treatment[80]. This therapy involves surgical tumor removal, TIL isolation and ex vivo expansion, followed by cell reinfusion to the patient [123]. However, most solid tumors exhibit low immunogenicity and lack TILs, creating major obstacles in TIL acquisition [180]. OVs, whether armed or unarmed, can induce immunogenic cell death, releasing tumor-associated antigens and recruiting tumor-associated neutrophils. This process converts "cold" tumors into TIL-rich "hot" tumors, making OVs ideal partners for TIL therapy [181, 182] (Fig. 7).

#### Synergistic mechanisms of immune modulation

##### OVs enhance the activation and persistence of TILs

In recent research, scientists utilized OVs to transform tumor cells into artificial antigen-presenting cells (aAPCs). Using a herpes simplex virus 1 (HSV-1)-based OVs encoding OX40L and IL12 (OV-OX40L/IL12), they provided local signals for T cell activation. Infected tumor cells showed enhanced expression of antigen-presenting cell (APC) markers, improving T cell activation and killing in TIL coculture [183]. This combination therapy achieved complete regression in patient-derived xenograft and syngeneic mouse tumor models, inducing antitumor immune memory. The strategy also conferred aAPC properties to tumor cells, activated T cells, and reprogrammed macrophages toward M1-like phenotype in the TME.

Another study found synergistic antitumor effects when combining OVs with TIL therapy, attributed to viral infection and T cell immune stimulation [184]. Hamster studies demonstrated 100% CR rates using TILT-123 (OVs encoding TNF-α and IL-2) combined with TIL therapy. Moreover, pre-incubating TILT-123 with TILs before systemic injection enhanced viral tumor delivery. This dual administration approach delivers both components as a single self-amplifying product: TILs facilitate TILT-123 delivery, while viral replication recruits and enhances TIL cytotoxicity. In vitro studies confirmed efficient TILT-123 binding to lymphocytes, validating this dual administration strategy. This innovative approach has the potential to provide a more effective and systematic delivery method for OVs therapy when combined with TILs.

#### Overcoming immunosuppression

Research has shown that TILT-123 (Ad5/3-E2F-D24-hTNFa-IRES-hIL2), an OVs designed to deliver TNFα and IL-2, shows promise in promoting significant immune activation of local TILs. In ovarian cancer, this virus encoding TNF-α and IL-2 can counter immunosuppression and enhance TIL antitumor responses [185]. Its immunomodulatory effects may improve the clinical efficacy of TIL-based adoptive cell therapy in advanced ovarian cancer patients.

Research demonstrated that combining interleukin-2 (IL-2)-armed OVs with TIL therapy promoted tumor-specific TIL accumulation in hypoimmunogenic tumors, while reducing the proportion of exhausted PD-1^hi^ Tim-3^+^ CD8^+^ cells and Tregs. TILs isolated from tumors treated with IL-2-expressing oncolytic virus maintained high tumor specificity after ex vivo expansion. When transferred into mice bearing MC38 tumors, these expanded TILs significantly inhibited tumor growth and improved survival [186]. These findings collectively demonstrate the synergistic potential of combining OVs with TIL therapy to enhance solid tumor immunotherapy efficacy.

#### Clinical trials exploring combination therapy with Ovs and mRNA cancer vaccines

TILT-123 (igrelimogene litadenorepvec) is an engineered OVs that selectively replicates in cancer cells and expresses dual transgenes IL-2 and TNF-α, designed to enhance tumor immune infiltration and cytotoxic T-cell responses [187]. In clinical trial NCT04217473, a patient with ICI-resistant metastatic sinonasal mucosal melanoma achieved durable complete remission following TILT-123/TIL combination therapy [187]. Immune analysis demonstrated enhanced tumor infiltration of CD4^+^ and CD8^+^ T cells, initiated by TILT-123 and amplified by TIL infusion, while modulation of peripheral blood NK cell phenotypes further strengthened the immune response. At day 78, RECIST1.1 imaging showed a disease control rate of 38% (6/16) in the TILT-123/TIL combination cohort. This compares favorably to historical outcomes with TIL therapy alone in similar refractory melanoma populations, where disease control rates typically range from 15–25%. Particularly notable was the durable complete response achieved in one patient with mucosal melanoma, a subtype that has historically shown limited responsiveness to either OV or TIL monotherapy (response rates < 10%). The observation that PET imaging showed partial or minor responses in four patients by day 36, following TILT-123 administration but before TIL infusion, further suggests that the oncolytic virus component may enhance the tumor microenvironment in preparation for cell therapy, potentially creating synergistic effects beyond what either approach could achieve alone.

In a phase I, open-label, 3 + 3 dose-escalation multicenter trial (ClinicalTrials.gov Identifier: NCT04217473) [188], patients with stage IV melanoma received multiple intravenous and intratumoral injections of TILT-123, combined with one or two administrations of TILs, which were expanded from resected tumor tissue without preconditioning or postconditioning regimens. The most common adverse events (AEs) associated with TILT-123 were fever (63%) and injection site pain (44%), while TIL therapy-related AEs included fever (50%) and chills (24%). No dose-limiting toxicities were observed, and AE severity was not exacerbated by the combination therapy. Serious treatment-related AEs occurred in 31% of patients. At day 78, RECIST1.1 imaging showed a disease control rate of 38% (6/16), with two patients achieving responses: one (cutaneous) had an ongoing PR, and another (mucosal) achieved a durable complete response. SD lasting over 10 months was observed in two patients (uveal and cutaneous). PET imaging at day 78 confirmed disease control in 6 of 13 evaluable patients, with partial or minor responses in four. By day 36, following four TILT-123 injections and before TIL administration, PET imaging also indicated partial or minor responses in four patients. These findings demonstrate that the combination of TILT-123 and TIL therapy is safe, feasible, and clinically active, even in challenging metastatic melanoma subtypes (Table 7).

**Table 7:** Clinical trial of OVs combination therapy with TIL cell for solid tumor Treatment

Trial Number	Launch	Phase	Study Status	CAR-T cell target Agent	TIL Agent	Tumor type	Rfs
NCT04217473	2020	I	Recruiting	TILT-123	TIL	Metastatic melanoma patients	

#### Potential challenges and future directions

Although the combination of OVs therapy with TILs shows great promise in the field of cancer treatment, it also poses a number of challenges. It is essential to comprehend these challenges and explore potential future directions to advance the effectiveness of this combination approach. Cancer cells can develop various mechanisms to evade immune responses, such as downregulating MHC expression or secreting immunosuppressive cytokines. Combining OVs with TILs may face resistance from these evasion strategies. Developing improved delivery systems for OVs to ensure efficient and targeted delivery to the tumor site, thereby maximizing their therapeutic impact in combination with TILs.

### OVs combined with RNA-based cancer vaccines

**Fig. 8:**
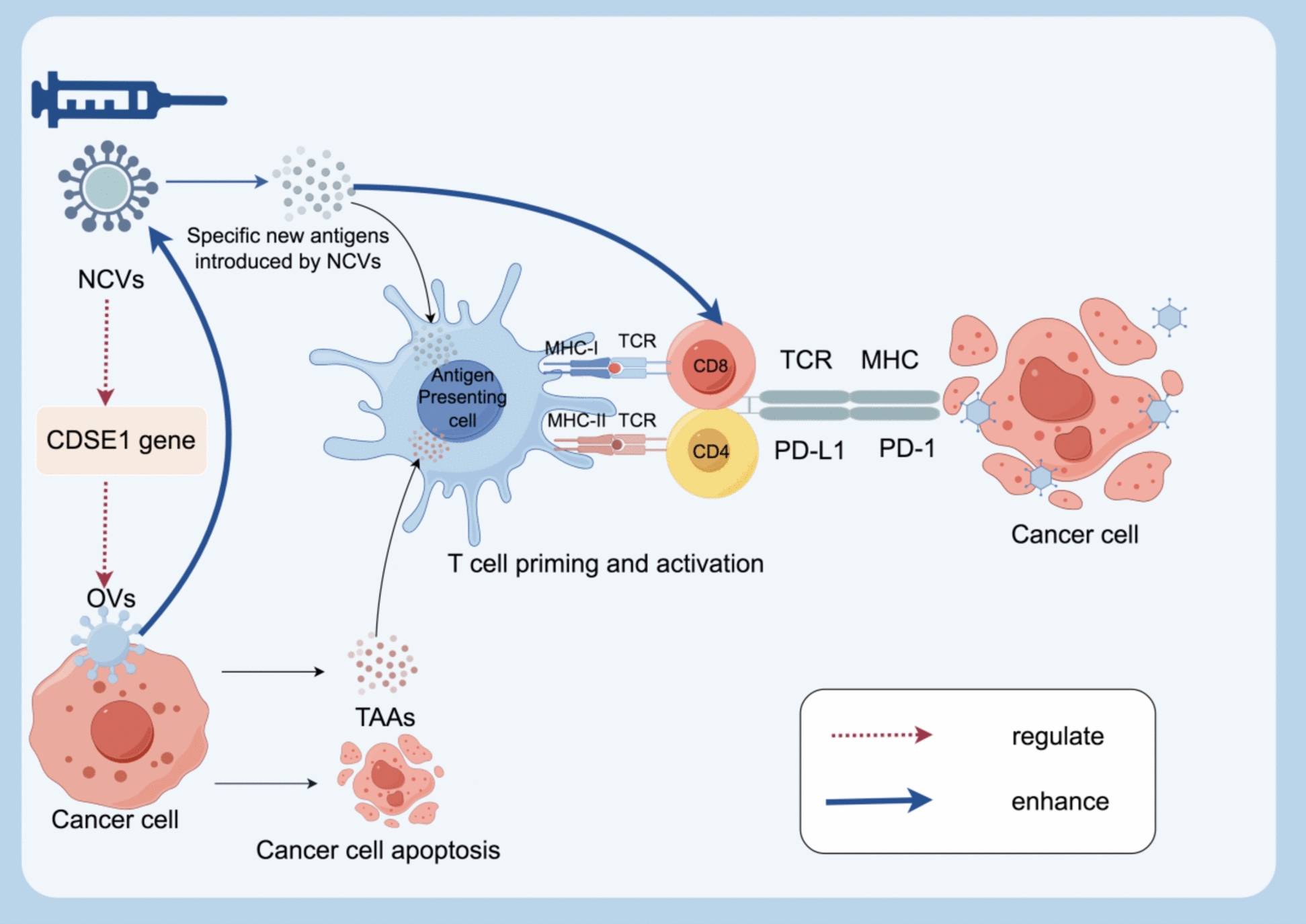
Mechanisms of Oncolytic Virus (OVs) and Neoantigen Cancer Vaccine (NCV) Combination Therapy in Sustaining Enhanced Anti-Tumor Immunity. OVs can directly dissolve tumor cells, simultaneously releasing antigens to enhance the immune system's recognition of tumor cells. In addition, they can enhance the anti-tumor effect of DC vaccines by increasing the infiltration of CD8 + T cells in the tumor microenvironment (TME). Furthermore, thanks to the priming effect of the NCVs, the designed specific neoantigens can target CDSE1 gene mutations to maintain or enhance immune responses. In conclusion, the combination therapy of these two methods can activate and sustain a continuous immune response, leading to more prolonged and effective anti-tumor effects (abbreviation: TTAs: full name: tumor-associated antigens)

Combining OVs with RNA-based cancer vaccines is a promising approach in cancer therapy. Both OVs and RNA-based cancer vaccines target cancer cells, but they do so through different mechanisms, and their combination can enhance the overall effectiveness of the treatment. OVs and RNA-based cancer vaccines function through complementary mechanisms. OVs can induce an inflammatory tumor microenvironment and release antigens, while RNA-based cancer vaccines contribute to the enhanced recognition of specific neoantigens by the immune system. Furthermore, OVs play a role in increasing the presentation of tumor antigens, including neoantigens, potentially amplifying the immune response initiated by RNA-based cancer vaccines. The collaborative action of these two therapeutic modalities may lead to a synergistic activation of the immune system, resulting in a more robust and sustained anti-tumor response. This combined strategy capitalizes on the unique strengths of both OVs and RNA-based cancer vaccines, providing a comprehensive and potentially more effective approach in cancer treatment (Fig. 8).

#### Synergistic mechanisms of immune modulation

##### Overcoming the immune escape resistance of tumor cells

Tumor cells treated with OVs can evolve mechanisms to evade the virus, diminishing treatment efficacy. In a clinical trial using interferon beta-expressing lysosomal vesicular stomatitis virus (VSV-IFNβ), some patients experienced aggressive relapses after initial remission. Notably, researchers identified the predictable mutations in the CDSE1 gene, which normally inhibits virus replication. Mutations in CDSE1, serving as a "fallback" for tumor cells, also produce a unique antigen recognized by immune system T cells. To combat predictable resistance, researchers developed a vaccine targeting the mutated CDSE1 gene. Combining lysosomal virus therapy with this vaccine effectively reduced the size of tumors and prolonged the survival of mice in experimental studies [189].

##### Overcoming immunosuppression

OVs therapy is a promising strategy to counteract immunosuppression and reveal TAAs; thus, it could work synergistically with DC vaccines. A study has demonstrated that OVs M1 (OVM) significantly enhance the antitumor effects of DC vaccines in several syngeneic mouse tumor models. This enhancement is attributed to the increased infiltration of CD8^+^ effector T cells in the TME [190]. Moreover, OV-induced oncolysis triggers the release of tumor-associated neoantigens, fostering a robust tumor-specific adaptive immune respons. Oncolysates obtained from tumor cells infected with OVM induced oncolysis triggers the release of tumor-associated neoantigens, strongly activate DCs and fostering a robust tumor-specific adaptive immune response. These findings underscore the feasibility of using OVM-infected tumor oncolysates to activate DC vaccines—a strategy that not only provides TAAs but also promotes DC maturation (Table 8).

**Table 8:** Preclinical study of OVs combination therapy with NCV for solid tumor Treatment

OVs agent	NCV agents	Potential mechanisms in combination therapy	Level of evidence	Tumor type	Rfs
Oncolytic vesicular stomatitis virus expressing interferon beta (VSV-IFNβ)	CSDE1^P5S^ generates an Escape-Associated Tumor Antigen (EATA)	CSDE1^P5S^ acts as an inhibitor of VSV replication facilitating escape from viral oncolysis, although tumor cells readily evolve to escape viral therapy, the oncolytic virus can, if given sufficient time, also itself evolve to complement those mutations that occur in its replication substrate	Murine melanoma (B16) and human HCC (Hep3B); Human Mel888 melanoma cells	Melanoma and HCC	[189]
Oncolytic virus M1 (OVM)	DC vaccines	Downregulate SIRPα in DCs and CD47 in tumor cells and upregulates PD-L1 in DCs,	Mouse models of melanoma (B16), colorectal (CT-26), renal (RM-1), and pancreatic cancer (Pan02)	Melanoma, colorectal, renal, hepatic and pancreatic cancer	[190]
rVSV-LCMVG	mRNA cancer vaccine express gp33 epitope	Induce the generation of virus-specific T cells and inhibited tumor cell proliferation	Mouse models of melanoma (B16)	Melanoma and HCC	[453]

#### Potential challenges and future directions

While the combination of OVs and RNA-based cancer vaccines shows promise, several challenges need to be addressed. The identification of patient-specific neoantigens requires advanced genomic and bioinformatic analyses, which may not be universally available or efficient. Consequently, there is a pressing need to enhance the identification and validation of neoantigens [191]. Furthermore, there is a need to incorporate tumor-specific targeting ligands, optimize viral vectors, or improve delivery using nanotechnology to precisely target and OVs to tumors while minimizing off-target effects [192]. Absolutely, promoting international collaboration is crucial for advancing research in OVs and RNA-based cancer vaccines combinations. Large-scale clinical trials that involve collaborative efforts among researchers, institutions, and countries can significantly contribute to the validation of the safety and efficacy of these innovative therapeutic combinations.

### Combination of ICI with emerging CAR-based therapies

In addition to CAR-T cells, emerging engineered cellular therapies such as CAR-NK cells and CAR macrophages represent innovative and promising platforms for solid tumor immunotherapy, especially when combined with ICIs in DDI and DDI + 1 strategies. CAR-NK cells harness the natural cytotoxicity of NK cells alongside CAR-mediated specificity, offering several distinct advantages over CAR-T cells, including lower risk of cytokine release syndrome and graft-versus-host disease, and the potential for off-the-shelf allogeneic products [193]. Importantly, CAR-NK cells retain their innate killing mechanisms, enabling them to target both antigen-positive and antigen-negative tumor cells, thereby reducing antigen escape [194]. When combined with ICIs, CAR-NK cells and T cells act synergistically to generate more robust antitumor immunity, with PD-1/PD-L1 blockade shown to enhance NK cell persistence and cytokine responsiveness. Moreover, CAR-NK cells have demonstrated superior tumor infiltration in immunosuppressive microenvironments, and their ability to eliminate Tregs and myeloid-derived suppressor cells further supports their integration into immunotherapeutic combinations. Ongoing clinical trials, including NCT03940820 and NCT04623944, are investigating CAR-NK cell therapies with ICIs in hematologic and HER2^+^ solid tumors, showing favorable safety profiles and early efficacy signals. NK cell transfer has shown promise in overcoming resistance to PD-(L)1 therapy in aged populations [195]. TCR-T cell therapy offers novel insights for solid neoplasm treatment [196]. The field is further advanced by comprehensive understanding of NK cell biology [197] and their clinical applications [198].

In parallel, CAR macrophages (CAR-M) represent a novel class of cellular immunotherapy that leverages the tumor-homing and plasticity of macrophages. CAR-M therapies can remodel the tumor microenvironment by maintaining pro-inflammatory (M1-like) phenotypes and counteracting immunosuppressive M2 macrophages. Their superior phagocytic activity and antigen-presenting function make them ideal candidates for combination with ICIs, which support sustained T cell activation and immune memory [199]. Preclinical studies have shown that CAR-M cells enhance T cell priming, increase tumor infiltration, and directly clear tumor cells via phagocytosis, complementing ICI-mediated T cell cytotoxicity [200]. Although clinical development of CAR-M is still in early stages, these strategies offer unique opportunities to overcome key limitations in solid tumor immunotherapy. Together, the integration of CAR-NK and CAR macrophages into DDI frameworks broadens the scope of cellular immunotherapy, providing mechanistically complementary, potentially less toxic, and more adaptable tools for precision oncology.

## Safety considerations in CAR-T based immune combinations

CAR-T cell therapies have revolutionized treatment for hematologic malignancies, but their application in solid tumors often requires combination strategies to overcome immune evasion. Understanding CAR T-cell hematological toxicities, including their manifestations, mechanisms, and management strategies, is crucial for safe implementation of combination therapies [201]. These combinations—such as CAR-T with ICIs, oncolytic viruses, or RNA vaccines—can significantly augment therapeutic efficacy while concurrently intensifying the risks of severe immune-related toxicities, particularly cytokine release syndrome (CRS) and immune effector cell-associated neurotoxicity syndrome (ICANS). The underlying mechanisms of this augmented toxicity are multifactorial: ICIs may enhance CAR-T expansion and cytokine production by removing T-cell inhibitory pathways; oncolytic viruses can trigger pathogen-associated molecular pattern recognition, amplifying inflammatory cascades; RNA vaccines stimulate innate immunity via toll-like receptors, compounding cytokine signaling. Clinical data support these concerns. For instance, in a phase I trial combining EGFRvIII-targeted CAR-T with pembrolizumab in glioblastoma, CRS presented with delayed onset (median 7 days vs. 1–3 days in monotherapy) and prolonged duration. Similarly, the CLDN6-CAR-T and RNA-LPX vaccine combination resulted in CRS in 75% of patients, compared to 50% in CAR-T alone. Neurotoxicity risk is especially relevant in CNS tumors or when targeting CNS-expressed antigens, requiring intensified neurologic monitoring. Risk stratification should account for tumor burden, inflammatory biomarkers (e.g., ferritin, CRP, IL-6), autoimmune history, and organ function. Preventive strategies include use of suicide switches (e.g., inducible caspase-9), split-signaling CARs, and sequential administration (e.g., priming with low-dose CAR-T followed by ICI after peak CRS window). Prophylactic tocilizumab or corticosteroids may be considered in high-risk patients, although these must be balanced against the risk of dampening CAR-T efficacy. Antiepileptics may be warranted for CNS-directed combinations. Monitoring should include daily neuro exams, inflammatory marker tracking, and inpatient observation for 7–10 days post-infusion. Treatment of CRS includes tocilizumab with early intervention (even at modified grade 1) and adjuncts like anakinra for IL-1-driven toxicity. ICANS management remains challenging due to limited steroid penetration in the CNS; novel approaches, such as selective IL-1 blockade or JAK/STAT inhibition with agents like ruxolitinib, show promise. Future advances in biomarker-guided interventions, refined CAR engineering, and targeted anti-inflammatory therapies will be key to improving safety while maintaining efficacy in these potent but complex combinations.

## Safety considerations: managing iraes in DDI

While DDI enhances anti-tumor responses, it also increases the risk of irAEs, which may compromise patient safety and treatment continuity. These toxicities, resulting from excessive immune activation, can affect multiple organ systems and vary based on treatment combinations. Common irAEs include dermatologic, gastrointestinal, endocrine, hepatic, pulmonary, and neurologic complications. For instance, the CheckMate-9DW trial showed that nivolumab plus ipilimumab improved response rates (45% vs. 28%) but also led to higher toxicity (grade ≥ 3 irAEs: 32% vs. 15%). Among DDIs, dual immune checkpoint inhibitors (e.g., anti-PD-1/CTLA-4) have the highest irAE burden, while combinations with CAR-T, TILs, or oncolytic viruses (OVs) introduce additional risks like cytokine release syndrome (CRS). In contrast, ICI + RNA vaccine regimens are generally well tolerated but still require irAE vigilance due to the ICI component.

Effective irAE prevention begins with careful patient selection and risk stratification. Patients with autoimmune disease, organ dysfunction, or prior severe irAEs should be assessed with biomarkers (e.g., CRP, IL-6, NLR) and functional baselines. Strategies such as sequential drug administration (e.g., delayed ipilimumab post-nivolumab) and low-dose corticosteroid prophylaxis have reduced toxicity without compromising efficacy. Targeted prevention tools—like TNF-α blockade in high-risk patients or microbiome modulation (e.g., probiotics, dietary interventions)—are also gaining traction. Routine monitoring should include baseline organ function panels, structured symptom tracking, and early referral for grade ≥ 2 irAEs. Special considerations are required for CAR-T or OV-based regimens, which carry unique toxicity profiles including CRS and viral replication risks.

Management of irAEs must be tailored to both regimen type and toxicity severity. ICI-only regimens follow established algorithms: grade 1 irAEs may continue therapy, while grade 2–4 events require corticosteroids or treatment cessation. For cellular therapies or combinations with OVs, high-dose corticosteroids, IL-6 blockade, and neurologic monitoring are often necessary. Although RNA-based vaccine combinations typically produce mild symptoms, close observation remains essential. Elderly and comorbid patients may benefit from dose adjustments and enhanced supportive care. Rechallenge after irAE resolution is generally acceptable for grade 1–2 events but not for grade 3–4 toxicities. Looking ahead, predictive biomarkers, safer immunotherapeutic designs (e.g., suicide switches, tissue-specific targeting), and AI-assisted monitoring are poised to improve irAE prediction and management. Standardized reporting of irAEs in DDI trials is critical to ensure safety and inform future therapeutic optimization.

## Future research directions for further optimization of DDI strategy

DDI represents a frontier in cancer treatment, focusing on combining two distinct immunotherapeutic approaches to amplify the body's immune response against cancer cells. Future research will likely delve into optimizing combinations of ICIs with other forms of immunotherapy, such as cancer vaccines, adoptive cell therapy, and cytokine therapy. Additionally, expanding the scope of DDI to cover a broader range of cancer types, especially those resistant to single-agent therapies, is a critical area of exploration. The development of next-generation ICIs that target new pathways beyond PD-1, PD-L1, and CTLA-4 will be another significant focus, aiming to overcome resistance mechanisms and provide more durable treatment responses.

## Identifying a suitable potential third agent to enhance the efficacy of x combination therapy

Combining different types of immunotherapies, known as dual immunotherapy, mentioned above, has shown promising results in treating various cancers. To enhance the efficacy and overcome resistance, adding a third non-immunotherapy drug could provide broader therapeutic benefits [202]. This approach leverages different mechanisms of action, creating a more comprehensive attack on cancer cells [202]. Some commonly used drugs that could be potential candidates, such as: Tyrosine Kinase Inhibitors (TKIs), Anti-Angiogenic Agents, and Chemotherapy Agents.

TKIs can block specific enzymes involved in signaling pathways that promote cancer cell growth and survival [203]. Examples include Sorafenib, which targets multiple kinases involved in tumor growth and angiogenesis [203]; Lenvatinib, which inhibits multiple receptors like VEGFR and FGFR to hinder tumor angiogenesis and proliferation [204]; and Erlotinib, which specifically targets the epidermal growth factor receptor (EGFR) pathway [205]. Combining TKIs with dual immunotherapy can target both tumor cells directly and the supporting TME [206, 207]. However, challenges include managing increased adverse effects and understanding the complex interactions between TKIs and immunotherapies to optimize treatment protocols [208]. Anti-Angiogenic Agents, on the other hand, can inhibit the formation of new blood vessels that tumors need to grow [209]. Candidates such as Bevacizumab, a monoclonal antibody that targets VEGF to prevent angiogenesis; Aflibercept, which acts as a decoy receptor for VEGF [210]; and Pazopanib, a multitargeted TKI with anti-angiogenic properties, are being explored [211, 212]. Research is focused on improving immune cell infiltration into tumors and assessing the impact on tumor vasculature normalization to enhance immunotherapy efficacy. Challenges include determining the appropriate patient population and balancing the inhibition of angiogenesis with normal tissue repair and homeostasis [212]. Moreover, chemotherapy agents, which kill rapidly dividing cancer cells through various mechanisms, also hold potential [213]. Examples include Cisplatin, which causes DNA cross-linking and apoptosis; Paclitaxel, which stabilizes microtubules and prevents cell division; and Doxorubicin, which intercalates DNA and inhibits topoisomerase II, leading to apoptosis [214–216]. Research is investigating the combination of chemotherapy with dual immunotherapy to enhance immunogenic cell death and the release of tumor antigens [217]. Key challenges involve mitigating the immunosuppressive effects of chemotherapy on immune cells and optimizing combinations to reduce overlapping toxicities and improve patient tolerance [218].

Recent advances have also proposed novel strategies to improve dual immunotherapy outcomes. For example, combination strategies with PD-1/PD-L1 blockade continue to evolve [219]. Promising approaches include disrupting Notch signaling via HES1 in myeloid cells to reinvigorate antitumor T cell responses [220] and using an oral STING agonist MSA-2 together with an anti-TGF-β/PD-L1 bispecific antibody YM101 as an immune cocktail therapy for non-inflamed tumors [221]. These strategies complement conventional drugs by remodeling the tumor microenvironment and enhancing immune responsiveness.

**Fig. 9:**
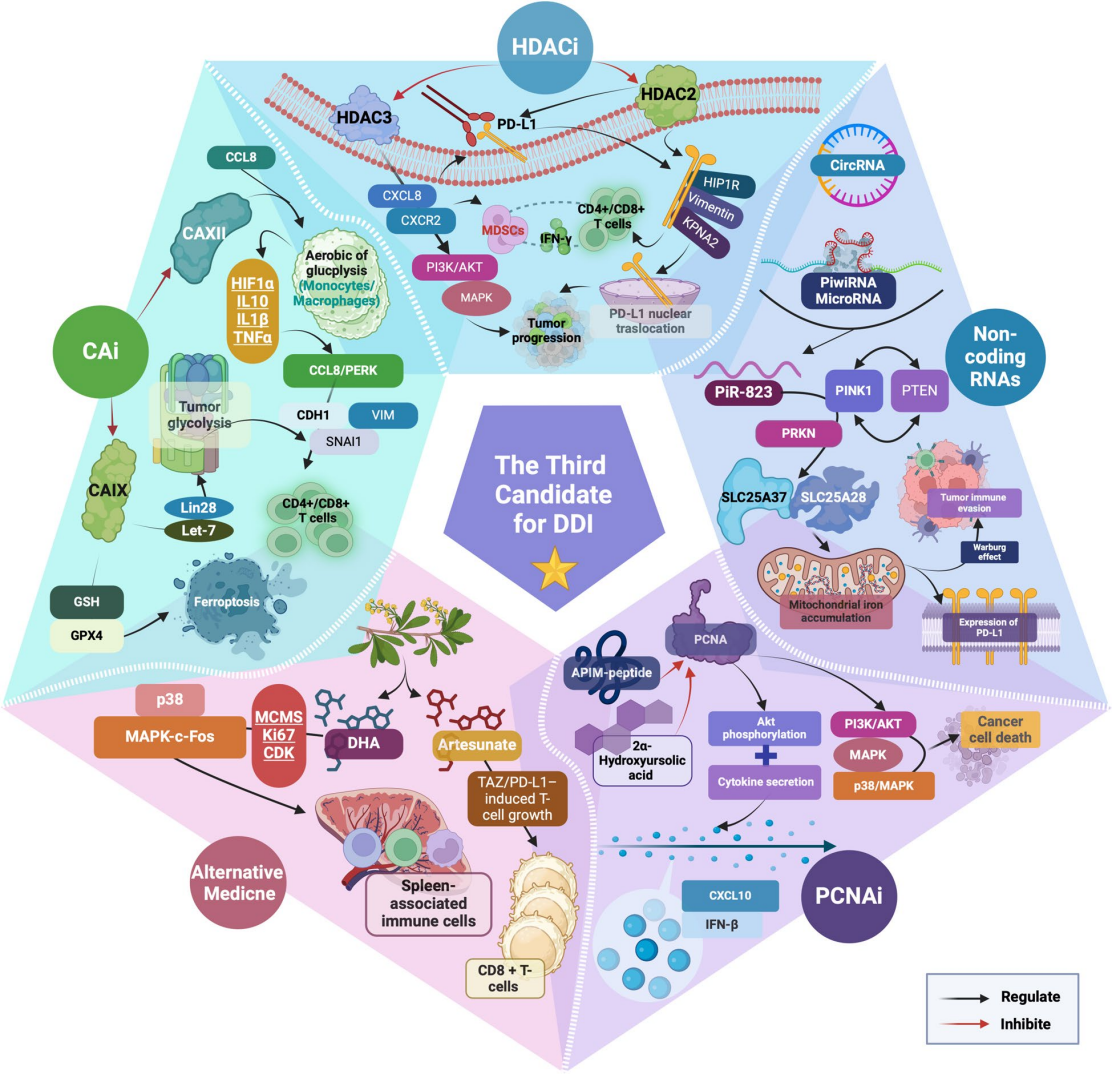
Mechanisms of a potential third agent in combination with DDI. PCNAis combined with DI could hold promise in cancer therapy by disrupting immune evasion and enhancing immune responses. Targeting PCNA with APIM-peptide affects key signaling pathways like PI3K/Akt and MAPK, which are involved in metastasis. In monocytes, APIM-peptide reduces Akt phosphorylation and cytokine secretion (e.g., CXCL10, IFN-β) after TLR stimulation. Additionally, 2α-hydroxyursolic acid inhibits PCNA expression in breast cancer cells, regulating the p38/MAPK pathway to reduce proliferation and promote apoptosis. HDACis enhance tumor antigen expression and immune-related genes, making tumor cells more visible to the immune system. When combined with DDI, such as PD-1/CTLA-4 inhibitors, HDACis like entinostat (ENT) and azacytidine (AZA) improve immune cell activity, reduce metastasis, and decrease immunosuppressive cells (e.g., FoxP3 + Tregs and G-MDSCs). HDACis also modulate tumor-infiltrating cell phenotypes and gene expression pathways, further boosting immune responses. Additionally, inhibiting HDAC3 suppresses the CXCL8/CXCR2 axis, enhancing the efficacy of immune checkpoint inhibitors (ICIs). CAi like SLC-0111 has shown promise in preclinical models of breast and brain cancer by inhibiting tumor growth and metastasis. In HCC, combining CAIi with anti–PD-1 antibodies enhanced therapeutic efficacy by reducing metastasis and improving survival. CAis like Tiliroside also restore immune cell function and reduce tumor progression. In triple-negative breast cancer, SLC-0111 increased pH shifts in the tumor microenvironment, sensitizing tumors to immune checkpoint blockade (ICB). Additionally, combining CAi with glutamine metabolism disruption promotes ferroptosis. CAIX-targeted CAR-T cells and combination therapies further enhance immune responses in solid tumors. ncRNAs can regulate the immune microenvironment and immune checkpoints to exert anti-tumor effects. For example, piR-823 promotes the degradation of PINK1, triggering a Warburg effect and inflammasome activation, which sensitizes tumors to DDI. Additionally, miR-149-3p downregulates PD-1, TIM-3, and BTLA, preventing tumor immune escape and enhancing T-cell function. MTL-CEBPA, a small activating RNA (saRNA), reduces the immunosuppressive effects of myeloid cells and boosts ICI efficacy by upregulating C/EBP-α

Incorporating a third non-immunotherapy agent with dual immunotherapy is for providing a multi-faceted approach to cancer treatment. Each category of agents presents unique mechanisms and challenges that must be addressed to maximize therapeutic benefits and minimize adverse effects. Here, we summarize several novel potential sensitizers for Dual Immunotherapy. Compared to commonly used traditional drugs, these sensitizers offer superior improvements in anti-tumor immunity and may exhibit better synergistic effects in cancer immunotherapy, holding significant research potential [222, 223] (Fig. 9).

### Potential candidates

#### Proliferating cell nuclear antigen inhibitors (PCNAis)

PCNA (Proliferating Cell Nuclear Antigen) inhibitor has also been considered as a potential sensitizer for DDI, which is essential for critical processes in cellular function including DNA replication and repair [224]. It can play a pivotal role in DNA replication and repair, and recent studies highlight its significant involvement in tumor immune regulation [225]. In tumors, PCNA facilitates cell cycle regulation and progression by ensuring efficient DNA replication and repair, which correlates with cancer development and progression [226]. Additionally, PCNAi contributes to immune evasion by enhancing the tumor cells' ability to avoid immune surveillance, often through interactions with immune inhibitory molecules like PD-1/PD-L1, thereby dampening the immune response [227]. PCNAi can also directly impact immune cells, such as DCs and macrophages, reducing their antigen-presenting capabilities and subsequently weakening T cell activation and anti-tumor functions [228–230]. PCNAi are capable of restoring the recognition and killing abilities of immune cells against tumor cells, potentially enhancing the efficacy of ICIs like PD-1 inhibitors, thereby amplifying the anti-tumor immune response [231]. Current research has developed small molecule inhibitors targeting PCNA, such as TX-101, PCNA-I1S, and T2AA, demonstrating significant anti-tumor activity in various cancer models [232–235]. Combining PCNA inhibitors with some conventional anti-cancer therapies, such as chemotherapy, radiotherapy, and immunotherapy, shows synergistic effects and promises improved overall therapeutic outcomes [224, 233, 236].

##### Current clinical trials of combing PCNAi with DDI or monotherapy

Evidence suggests that PCNA, when expressed on the surface of cancer cells, can interact with NKp44 to inhibit the antitumor function of NK cells. In this context, PCNAi has emerged as a potential immune checkpoint blockade strategy, capable of enhancing NK-cell-mediated antitumor responses [237–239]. A monoclonal antibody (mAb) targeting human PCNA, 14-25-9, has been shown to effectively recognize cell surface PCNA on both solid and leukemic cancer cell lines, as well as tumor cells derived from patient-derived xenografts. This antibody blocks the NKp44-PCNA immune checkpoint and overcomes the inhibitory effects of PCNA, thereby enhancing NK-cell antitumor responses both in vitro and in vivo [240]. Moreover, the enhancement of NK-cell function following the blockade of target-membrane-associated PCNA by mAb 14-25-9 does not rely on MHC-I molecules expressed by the target cells. At the intracellular level of NK signaling, both killer immunoglobulin-like receptor (KIR)- and NKp44-isoform-1-mediated signals are ITIM (immunoreceptor tyrosine-based inhibitory motif)-dependent, and they likely act as additive signals, similar to the concept of NK cell education [241]. This has been demonstrated by the increased secretion of IFN-γ, a cytokine, and higher expression of CD107a, a marker for NK cell activity, when NK cells were exposed to multiple myeloma cells treated with mAb 14-25-9. Furthermore, NK cells treated with this antibody exhibited a much stronger anti-malignant response against multiple myeloma cells. In addition, recent studies indicate that co-culturing DCs with autologous cytokine-induced killer (CIK) cells can suppress PCNA expression and promote the expression of caspase-3, a key protein in the apoptosis pathway, which subsequently induces apoptosis in liver cancer stem cells [231].

Hence, combining PCNAi with DDI holds significant research value, offering extensive opportunities for further study and potential clinical benefits (Table 9).

**Table 9:** Clinical trial and preclinical study of dual immunotherapy combination therapy with the 3rd agent

Trial Number	Launch	Phase	Study status	3rd agent	Immunotherapy Agents	Potential Mechanisms In combination therapy	Level of evidence	Tumor type	Rfs
–	–	–	–	mAb 14–25-9	mAb 14–25-9	Enhance the functional activity of NK cells overexpressing NKp44-Iso;	Murine Head and neck squamous cell carcinoma models (PDX)	Head and neck squamous cell carcinoma	[240]
–	–	–	–	DC- Cytokine-induced killer cells (CIK) cell	DC- Cytokine-induced killer cells (CIK) cell	Suppress PCNA expression and promote the expression of caspase-3 protein, subsequently inducing apoptosis in liver cancer stem cells	Liver cancer stem cells (LCSC) generated from human and HCC cells model; Murine LCSC carcinoma models;	HCC	[231]
NCT02638090	2016	I/IB	Active, not recruiting	Vorinostat	Pembrolizumab	Elevated presence of CD8 + T cells in the stroma	Clinical	NSCLC	[454]
NCT04357873	2010	II	Active, not recruiting	Vorinostat	Pembrolizumab	–	Clinical	recurrent and/or metastatic squamous cell carcinoma	[455]
NCT03565406	2018	I	Termin-ted	Mocetinostat	Nivolumab + ipilimumab	Promoted accumulation of central memory CD8 and CD4 T cells from melanoma patients, and decreased percentages and suppressive activity of T regulatory cells and myeloid-derived suppressor cells	Clinical	Melanoma	[456]
NCT04133948	2021	I	Active, not recruiting	Panobinostat	Nivolumab; ipilimumab	–	Clinical	Resectable Muscle-invasive urothelial cancer	[457]
–	–	–	–	Panobinostat	oncolytic herpes viruses ( oHSV T3855) encode IL-12 and anti-PD-1 antibody	Enhanced virus replication mediated by the downregulation of IFN-β- and IFN-stimulated antiviral genes, as well as the cGAS/STING pathway	Murine glioma CT-2A and squamous cell carcinoma SCC7 models	Glioma and squamous cell carcinoma	[458]
NCT04133948	2020	I/II	Active, not recruiting	Domatinostat	Nivolumab + ipilimumab	Increased intratumoral T cell infiltration and the IFN-γ sign expression in melanoma	Clinical	Melanoma	[459]
–	–	–	–	Domatinostat	Anti-PD-1; Anti-CTLA4	Increase in tumor-reactive T cells and the frequency of CD206 + macrophages	Murine melanoma tumors model	Melanoma	[460]
NCT02453620	2016	I	Active, not recruiting	Entinostat	Nivolumab + ipilimumab	Alters CD8/FoxP3 ratios in certain patients and increased immune infiltration	Mouse models of breast cancer (NeuN and 4T1) and mirror the treatment scheme used in the clinical trial	Advanced solid tumors	[461]
–	–	–	–	Entinostat	Anti-PD-1;Anti-CTLA4	Increased tumor infiltration by G-MDSCs and changed the polarity of their immunosuppressive ability to a nonfunctional phenotype	Spontaneous mammary (NT2.5) tumors and metastatic pancreatic (Panc02) cancer model	Breast cancer and pancreatic ductal adenocarcinomas	[264]
–	–	–	–	Entinostat	Azacytidine; Anti-PD-1; Anti-CTLA4	Decrease in tumor-infiltrating FoxP3 + Tregs and increase in circulating granulocytic MDSCs	Murine colorectal (CT26) and breast (4T1) tumors model	Colorectal cancer and breast cancer	[263]
NCT04553393	2020	I/II	Unknown status	Chidamide	Decitabine-primed Tandem CAR19/20 engineered T cells	–	Clinical	Aggressive relapsed and/or refractory non-hodgkin's lymphoma patients with Huge tumor burden	[462]
–	–	–	–	Chidamide	Regorafenib; anti-PD-1; Anti-CTLA-4	Upregulated IFN pathway, macrophage gene, neutrophil gene, and T-cell gene signatures	Murine colorectal (CT26) tumors model	Colorectal cancer	[463]
–	–	–	–	CG-745	anti-PD-1	Induces and prolongs the T cell activation, while increasing the population of CD3 + /CD8 + T cells, CD3 + /CD56 + , and CD3-/CD56 + cells and decreasing the populations of CD4 + /CD25 + /Foxp3 + , and MDSC in vivo and in vitro	HCC cell model; Mouse HCC model and CRC model	HCC; CRC	[464]
–	–	–	–	trichostatin A	anti-CTLA4	Increase the number of activated CD4 + T lymphocytes;	Melanoma cell and melanoma mice model	Melanoma	[465]
NCT05470283	2022	I	Active, not recruiting	Acetazolamide	OBX-115 (an IL2-sparing engineered TIL cell therapy)	–	–	Metastatic melanoma	[466]
NCT04969354	2021	I	Recruiting	CAIX-targeted CAR-T Cells	CAIX-targeted CAR-T Cells	–	–	Advanced renal cell carcinoma	[467]
–	–	–	–	SLC-0111	Anti-PD-1; Anti-CTLA-4	Reduced the presence of Tregs and Th17 cells and increased the frequency of Th1 cells; Increases granzyme B production; Increases the frequency of CD4 + ICOS + T cells;	Murine melanoma (B16F10) and breast (4T1) tumors model	melanoma and breast cancer	[303]
–	–	–	–	CAIX-CAR-T	OAV-Strapin	Enhancing T cell persistence	Renal cancer cells 786O, ACHN, and OSRC-2; Murine renal cancer ( OSRC-2/Renca) model;	Renal cell carcinoma	[299]
–	–	–	–	CAi	Anti-PD-1;	Increased CD8 + T-cell ratios	Murine HCC (Hepa1-6) model	HCC	[290]
–	–	–	–	KRAS G12V targeted mRNA	Pembrolizumab	–	Case report	Pancreatic head cancer,NSCLC	[468]
NCT05392699	2022	I	Active	ABO2011	–	Regulation of TME	Clinical	Solid tumors	[469]
NCT05727839	2023	I	Active	JCXH-211	–	Enhanced translatability in immunosuppressed TME	Clinical	Solid tumors	[315]
NCT05097911	2021	I	Active	MTL-CEBPA	Atezolizumab	–	Clinical	Advanced solid tumors	[470]
NCT04105335	2019	I	Active	MTL-CEBPA	Pembrolizumab	–	Clinical	Advanced HCC	[471]

##### Potential mechanisms of applying PCNAi as the 3rd agent for DDI

The combination of PCNAis and ICIs for cancer treatment represents a novel and emerging field of research. The epidermal growth factor receptor (EGFR) and the stress-induced intracellular tyrosine kinase c-ABL have been identified as key regulators of tyrosine 211 phosphorylation of PCNA (pY211-PCNA). This modification partially stabilizes chromatin-associated PCNA, thereby promoting cancer cell proliferation, particularly in breast cancer [225, 242]. Inhibition of pY211-PCNA disrupts replication fork processivity, resulting in the generation of single-stranded DNA (ssDNA) through an MRE11-dependent mechanism. This accumulated cytosolic ssDNA activates an inflammatory response through the cGAS/STING pathway, which subsequently triggers anti-tumor immunity via NK cell activation. Furthermore, targeting PCNA with the APIM-peptide can modulate key signaling pathways, including the PI3K/Akt and MAPK pathways—two critical pathways that are involved in tumor metastasis due to their extensive crosstalk [225]. Targeting PCNA with the APIM-peptide in monocytes reduces Akt phosphorylation and decreases cytokine secretion, specifically CXCL10 and IFN-β, following TLR stimulation. The APIM-peptide also modulates key signaling pathways, including the PI3K/Akt and MAPK pathways, thereby influencing the cellular response to external stimuli [243]. 2α-Hydroxyursolic acid has been shown to inhibit the expression of PCNA in breast cancer cells, exerting anticancer activity by regulating the p38/MAPK signaling pathway. This regulation leads to the inhibition of breast cancer cell proliferation and the induction of apoptosis [244].

ATX-101, a novel cell-penetrating peptide containing APIM, is a PCNAi that disrupts PCNA-protein interactions and has shown significant anti-cancer activity as a single agent in multiple cancer cell lines and models [232, 245]. It has been investigated in several clinical trials and has demonstrated the ability to potentiate the effects of various anti-cancer treatments [232]. For example, in glioblastoma models, ATX-101 exhibited radiosensitizing properties, indicating its potential use alongside radiotherapy. Studies have shown that the combination of APIM-peptide and cisplatin alters the expression of proteins involved in cancer cell growth and cisplatin resistance, reduces the expression of genes related to the DNA damage response, and likely inhibits key pathways dependent on PCNA interactions [246]. Additionally, combination treatments targeting EGFR, ERBB2, and downstream PI3K/Akt and Ras pathways have shown downregulation of these cancer targets, along with reduced expression of JAK, STAT, and FAK1. Notably, ATX-101 has been shown to enhance the anti-cancer effects of the EGFR/HER2/VEGFR inhibitor AEE788, with proteome analysis indicating upregulation of Akt and MAPK signaling pathways, which are associated with tumor progression and cell survival [247, 248].l Preclinical data further support the potential of ATX-101 in combination with docetaxel, as it downregulates therapeutic targets and upregulates genes protective against prostate cancer. Although specific details on ATX-101 combined with immunotherapy are limited, its ability to disrupt PCNA interactions suggests it could potentially enhance the effectiveness of immunotherapeutic agents, providing a rationale for combining ATX-101 with therapies that modulate the immune system to target cancer cells [249].

AOH1160 and AOH1996 are also small-molecule compounds that target PCNA and have demonstrated promising anti-cancer activity [235, 250]. AOH1160 is an orally available compound that suppresses tumor growth in mice without causing significant side effects [235]. Mechanistically, it disrupts the binding of 3,3′,5-Triiodothyronine (T3), a known ligand of PCNA, to the PCNA protein. This interference impedes DNA replication and obstructs homologous recombination (HR)-mediated DNA repair. This leads to cell-cycle arrest, accumulation of unrepaired DNA damage, and increased sensitivity to cisplatin treatment [251].

AOH1996, another small-molecule compound, specifically binds to a region in the PIP-box binding pocket of PCNA, interfering with the interaction between PCNA and RNA polymerase II (RPB1). This interference prevents RNA polymerase II from properly binding to DNA, resulting in DNA double-strand breaks under transcription-dependent conditions [250]. Since cancer cells rely more heavily on transcription-coupled repair (TCR), AOH1996 selectively impacts cancer cells over normal cells. Currently, a phase I trial (ClinicalTrials.gov Identifier: NCT05227326) is investigating the side effects and optimal dosage of AOH1996 in patients with refractory solid tumors.

Recent investigations have centered on emerging small-molecule inhibitors, notably PCNA-I1S and T2AA, which specifically target PCNA through direct binding interactions. Experimental studies evaluating PCNA-I1S in combination with DNA-damaging agents demonstrated enhanced antiproliferative effects in prostate and lung cancer cell lines. This synergistic interaction suggests the potential utility of PCNA-I1S as a chemotherapy and radiotherapy sensitizer [233, 252]. In a parallel line of research, T2AA was found to impair the repair of interstrand DNA crosslinks and potentiate double-strand break formation, thereby increasing tumor cell sensitivity to platinum-based chemotherapeutics such as cisplatin [234, 253, 254]. Although research specifically on the combination of PCNAis and ICIs in cancer treatment is currently limited, the expanding field of combining ICIs with targeted therapies highlights the need for further exploration. While still in the early clinical stages, PCNAis show substantial potential in tumor immune regulation, offering a promising addition to anti-tumor immunotherapy strategies as ongoing research continues to clarify their mechanisms and clinical applications.

##### Potential for applying PCNAi as the third agent in DDI

Integrating PCNA inhibitors into dual immunotherapy regimens holds significant potential to enhance antitumor efficacy by disrupting immune evasion mechanisms and boosting immune responses. These inhibitors not only sensitize cancer cells to treatments like chemotherapy and radiotherapy but also target key pathways in cancer cells, positioning them as a promising addition to future immunotherapy strategies. By synergizing with HDAC inhibitors and ICIs, PCNAis could create a more robust and sustained immune response, offering a promising avenue for advancing cancer treatment.

##### Potential challenges for conducting related study

The success of dual immunotherapy often relies on the synergistic effects between agents. Optimizing the ideal candidate, dosage, and timing of PCNA-targeted agents in combination with other immunotherapies is a complex challenge that demands extensive preclinical and clinical testing. To overcome these challenges, further research and development are crucial to refine the use of PCNAis in dual immunotherapy and ensure their safe and effective integration into cancer treatment protocols.

#### Histone deacetylases inhibitors (HDACis)

HDACis plays a crucial role in tumor development and progression by altering gene expression through histone deacetylation, leading to the silencing of tumor suppressor genes and the promotion of oncogenic pathway [255]. HDACis are not only used as standalone treatments for cancer but have also been increasingly found to improve anti-tumor immunity and synergize with immunotherapies, such as ICIs, in treating cancer [256]. These drugs also have the potential to enhance the effectiveness of dual immunotherapy in combating cancer.

##### Current clinical trials of combing HDACis with DDI or monotherapy

A novel three-drug combination, including an HDACi, has shown promising results in patients with advanced HER2-negative breast cancer. This regimen, which combines an HDACi with two types of checkpoint inhibitors, was evaluated in a multicenter Phase Ib study (NCT02453620). The therapy, consisting of entinostat, nivolumab, and ipilimumab, achieved a 25% OR rate and a 40% clinical benefit rate among patients. Notably, in patients with triple-negative breast cancer—a group with limited treatment options—the OR rate was 40% with this combination [257]. These findings indicate that pretreatment with the HDACi entinostat, followed by dual immune checkpoint inhibition, is a safe and promising strategy for treating metastatic breast cancer, meriting further clinical evaluation in a Phase II study.

A Phase 1b clinical trial (ClinicalTrials.gov Identifier: NCT03565406) investigated the therapeutic potential of combining the selective HDAC inhibitor mocetinostat with dual checkpoint blockade (ipilimumab/nivolumab) in patients with unresectable stage III/IV melanoma [258]. The treatment regimen consisted of two phases: a 12-week induction period combining oral mocetinostat (administered thrice weekly) with standard doses of ipilimumab/nivolumab (administered triweekly), followed by a 12-week maintenance phase of biweekly nivolumab plus continued mocetinostat. The study's primary objectives included safety profiling, determination of recommended Phase 2 dosing, efficacy assessment, and biomarker analysis. Of the ten enrolled participants, nine received mocetinostat at 70 mg while one received 50 mg. The 70 mg cohort demonstrated an 89% objective response rate, whereas disease progression was observed in the single patient receiving 50 mg. All participants experienced Grade 2 or higher adverse events, with Grade 3–4 toxicities occurring in 60% of cases. Immunological analyses of peripheral blood mononuclear cells (PBMC) and serum samples revealed significant reductions in myeloid-derived suppressor cell populations and a downward trend in anti-inflammatory monocyte phenotypes. Serological assessment demonstrated marked elevation in granzyme A and TNF levels, accompanied by upward trends in granzyme B and IFN-γ expression.

In another study, a feasibility trial (ClinicalTrials.gov Identifier: NCT04871594) explored the addition of domatinostat to pre-operative ICIs in patients with muscle-invasive urothelial cancer. (Study Details | Pre-operative Immunotherapy in Stage II-III Urothelial Cancer | ClinicalTrials.gov). Depending on the results, this study could be adapted or expanded into a Phase 2 study.

The DONIMI trial, a Phase 1b study (ClinicalTrials.gov Identifier: NCT04133948), tested the combination of domatinostat and nivolumab or nivolumab monotherapy in patients with a high IFN-gamma signature, and domatinostat combined with nivolumab or domatinostat combined with nivolumab and ipilimumab in patients with a low IFN-gamma signature[259]. This trial involved patients with de-novo or recurrent macroscopic stage III cutaneous melanoma or melanoma of unknown primary origin. Other HDACi ongoing trials have been displayed in Table 9.

##### Potential mechanisms of applying HDACi as the 3rd agent for DDI

HDACis are a class of compounds that interfere with the function of histone deacetylases [260]. These inhibitors can increase the expression of tumor antigens and other immune-related genes, making tumor cells more recognizable to the immune system. When combined with ICIs, HDACis can modify the tumor microenvironment, enhancing the infiltration and activity of immune cells activated by ICIs. This combination can result in a more robust anti-tumor immune response [256, 261]. Synergy between HDACis and various immunotherapies, including ICIs, has been reported in preclinical studies [262].

Research demonstrated significant effects when combining HDACis (entinostat and azacytidine) with PD-1/CTLA-4 blockade. All five mice treated with anti-PD-1/CTLA-4 antibodies plus HDACis showed complete primary tumor elimination without metastasis [263]. In contrast, mice receiving anti-PD-1/CTLA-4 alone retained large primary tumors with an average of 11 lung metastases [263]. Moreover, adding entinostat and azacytidine significantly reduced tumor-infiltrating FoxP3^+^ Tregs compared to untreated or anti-PD-1/CTLA-4 antibody-only groups. This combination therapy also decreased circulating granulocytic myeloid-derived suppressor cells to levels comparable to tumor-free mice [263].

Another study demonstrated that combining entinostat with anti-PD-1, anti-CTLA-4, or both significantly improved tumor-free survival in HER2/neu transgenic breast cancer and Panc02 metastatic pancreatic cancer mouse models. Entinostat enhanced granulocytic myeloid-derived suppressor cells infiltration while converting their immunosuppressive capacity to a nonfunctional phenotype. Flow cytometry and gene expression profiling revealed alterations in functional molecule expression and genetic pathways related to proliferation and motility in treated tumors [264].

Additionally, existing evidence indicates that elevated baseline CXCL8 (IL-8) levels in plasma are associated with poor clinical outcomes in patients undergoing immunotherapy, such as nivolumab or ipilimumab, suggesting that CXCL8 could serve as a biomarker for predicting the efficacy of ICI treatment [265]. Similarly, CXCR2 (C-X-C chemokine receptor type 2), which is often overexpressed in hepatocellular carcinoma (HCC) patients, has also been recognized as an indicator of poor prognosis in various cancers, including liver cancer [266]. The interaction between CXCL8 and CXCR2 activates downstream signaling pathways that suppress antitumor immunity, promote tumor cell invasiveness, and facilitate immune evasion [267, 268]. This makes the CXCL8/CXCR2 axis not only a potential prognostic biomarker for HCC patients but also a promising therapeutic target [267, 269]. More importantly, recent studies have shown that inhibiting HDAC3 can suppress the CXCL8/CXCR2 axis, leading to reduced infiltration of granulocytic myeloid-derived suppressor cells (G-MDSCs) and enhanced activation and infiltration of T cells [181]. This improvement in the tumor immune microenvironment can stimulate the conversion of "cold tumors" to "hot tumors" and enhance the efficacy of PD-1/PD-L1 checkpoint inhibitors [270, 271].

Consequently, the combination of HDACi with DDI is assumed to be an extremely novel but promising therapeutic strategy with significant potential for clinical application and research. This approach offers new opportunities to further improve the overall prognosis of cancer patients.

##### Potential for applying HDACi as the third agent in DDI

Based on the definition of DDI as the strategic combination of two distinct types of antitumor immunotherapies to synergistically enhance the immune system's ability to combat cancer, HDACis have shown potential to significantly amplify DDI’s effectiveness. HDACis, by increasing the expression of tumor antigens and modifying the tumor microenvironment, enhance the infiltration and activity of immune cells activated by ICIs, resulting in a more robust antitumor response. Clinical studies have demonstrated promising results with combinations of HDACis, such as entinostat, with ICIs like nivolumab and ipilimumab, particularly in challenging cancer types like advanced HER2-negative breast cancer and melanoma. These combinations not only improved response rates but also indicated enhanced survival outcomes. Preclinical studies further support the synergistic effects of HDACis with ICIs, showing eradication of primary tumors, reduction of metastases, and significant modulation of immune-suppressive cells. Given these findings, integrating HDACis into DDI regimens could offer a powerful strategy for enhancing antitumor immunity and improving clinical outcomes in cancer therapy.

##### Potential challenges for conducting related study

These studies demonstrate that pretreatment with HDACis combined with dual ICI therapy is a safe and promising strategy for advanced cancer, warranting further clinical evaluation. However, whether the addition of HDACis will increase the incidence of irAEs in DDI therapy remains unexplored. Additionally, significant research efforts will undoubtedly be required to determine factors such as patient selection for HDACis, dosage, and timing of administration, both in laboratory and clinical studies.

#### Carbonic anhydrase inhibitors (CAis)

CAis are a class of drugs that inhibit the enzyme carbonic anhydrase, which is responsible for converting carbon dioxide and water into bicarbonate and protons [272]. This enzyme plays a crucial role in pH balance, fluid regulation, and ion transport. Traditionally, CAis have been used to treat conditions like glaucoma, epilepsy, and altitude sickness, but their role in cancer therapy has gained attention, particularly for their ability to modulate the tumor microenvironment[273]. In tumors, certain carbonic anhydrase isoforms, such as CAIX and CAXII, are overexpressed, especially under hypoxic conditions [274]. These isoforms help cancer cells survive and proliferate by maintaining an acidic environment that promotes tumor growth and impairs immune cell function [275]. By inhibiting CAIX and CAXII, CAis can neutralize the acidic tumor microenvironment, improving immune cell infiltration and enhancing the efficacy of immunotherapies such as ICIs [276]. CAIX is associated with poor prognosis, and its inhibition can disrupt the tumor's pH regulation, making cancer cells more vulnerable to immune attack [277]. Similarly, CAXII inhibitors complement other treatments by normalizing the tumor environment [278]. CAIs, particularly those targeting CAIX and CAXII, offer a novel approach in cancer therapy, enhancing immune modulation and improving outcomes for patients with resistant tumors [273, 279, 280].

##### Potential for applying CAi as the third agent in DDI

The initiation of Carbonic anhydrase XII inhibitors (CAXIIis) in cancer treatment has shown encouraging early results. A phase I clinical trial (ClinicalTrials.gov Identifier: NCT02215850) of SLC-0111, a selective inhibitor of both CAIX and CAXII, demonstrated safety in 17 patients with advanced solid tumors across 10 different cancer types. The trial established a recommended Phase II dose of 1000 mg/day [281]. Following these results, a Phase Ib study combining SLC-0111 with gemcitabine for metastatic pancreatic ductal cancer patients has been underway since 2018 [282].

Acetazolamide, a broad-spectrum carbonic anhydrase inhibitor, is being evaluated in combination with radiochemotherapy for lung cancer (ClinicalTrials.gov Identifier: NCT03467360) and with temozolomide for brain cancer (ClinicalTrials.gov Identifier: NCT03011671) [273].

OBX-115, an engineered TIL therapy, is currently under evaluation in a Phase I trial (ClinicalTrials.gov Identifier: NCT05470283) for patients with ICI-resistant metastatic melanoma. Notably, the regimen includes lymphodepletion followed by OBX-115 administration in combination with acetazolamide, a CAXII inhibitor. Preliminary results indicate that this combination is well-tolerated, producing deep and durable responses, with the potential to achieve complete responses (CRs) in patients with resistant melanoma [283].

Immunotherapy targeting CAIX with CAR-T cells is also gaining attention. A clinical trial with 12 patients with CAIX-expressing metastatic renal cell carcinoma (RCC) treated with CAR T cells against CAIX showed that low-dose CAR T cells induced antigen-specific liver toxicity due to CAIX expression on bile duct epithelium. However, pretreatment with a CAIX monoclonal antibody (mAb) reduced this liver toxicity and enhanced CAR T cell persistence. Although no clinical responses were recorded, the study demonstrated the antigen-specific effects of CAIX-targeted CAR T cells and showed that “on-target” toxicity could be mitigated with antigen blockade [284].

Further exploration in this area includes a Phase I trial (ClinicalTrials.gov Identifier: NCT04969354) enrolling 20 patients with metastatic RCC refractory to prior therapies to assess CAIX-targeted CAR T cells. Prior to CAR T cell infusion, a CAIX mAb is injected into the hepatic artery to minimize liver toxicity [285].

Additionally, CAIX-targeted immune therapies, such as autologous DCs transduced with a granulocyte–macrophage colony-stimulating factor/CAIX fusion construct (DC-AdGMCAIX), have shown a CAIX-specific immune response in Phase I trials for renal cancer (ClinicalTrials.gov Identifier: NCT01826877), underscoring the potential of CAIX-targeted approaches [286]. Other relevant clinical results are presented in Table 9.

##### Potential mechanisms of applying CAXIIi as the 3rd agent for DDI

Therapeutically targeting Carbonic Anhydrase IX (CAIX) in hypoxic solid tumors with the small-molecule inhibitor SLC-0111 has shown significant inhibition of tumor growth and metastasis in preclinical models of breast and brain cancer [287–289].

A recent study showed that combining CAIX inhibitors (CAXIIis) with anti–PD-1 antibodies enhanced therapeutic efficacy in HCC models. This combination therapy significantly attenuated tumor growth, reduced metastasis, and improved OS in vivo. CAXIIis facilitated macrophage adaptation to the acidic tumor environment and promoted the production of CCL8 by macrophages, which in turn enhanced cancer cell epithelial-mesenchymal transition (EMT) and metastasis. Additionally, CAXIIis increased macrophage apoptosis and reduced their proportion in CD45^+^ cells, a response not seen with anti-PD-1 treatment alone [290]. Tiliroside, a CAXIIis, has been shown to inhibit HCC development through the E2Fs/Caspase-3 axis [280]. Additionally, Tiliroside reduces Tim-3 expression on depleted CD4 + and CD8 + T cells, restoring their function. This dual action—targeting both tumor growth and immune cell dysfunction—may enhance the efficacy of immune therapies [291]. As a novel class of CAXII inhibitors, glycosylcoumarins further demonstrate the therapeutic potential of CAXII inhibition across various cancers. They exert antitumor effects on T-cell acute lymphoblastic leukemia and lymphoma cells by inhibiting the mTOR/Akt pathway and downregulating c-myc [292].

In triple-negative breast cancer (TNBC), studies have shown that chemotherapeutic agents targeting CAIX, such as SLC-0111, significantly increased extracellular pH while decreasing intracellular pH and extracellular lactate. This shift created a hostile tumor microenvironment that inhibited TNBC progression and sensitized tumors to ICB, enhancing immune cell-mediated killing. These effects were associated with a stronger Th1 response and reduced metastasis, particularly in metastatic melanoma and basal-like breast cancer [279, 293]. The combination of SLC-0111 with anti–PD-1 and anti–CTLA-4 treatment preserved the SLC-0111-induced Th1 cell population, while maintaining low frequencies of Tregs and eliminating Th2 cells. Additionally, this triple therapy increased the frequency of T-bet + CD8 + tumor-infiltrating lymphocytes (TILs) within the tumor microenvironment (TME), enhanced the frequency of CD4^+^ ICOS^+^ T cells, promoted granzyme B production, and reduced PD-1 expression. Furthermore, higher CAIX expression was associated with lower levels of key T cell markers—CD3E, CD8A, and CD4—within the TME. Reduced expression of these markers in the TME was linked to poorer patient outcomes, suggesting that CAIX could serve as both a prognostic marker and a therapeutic target in cancer treatment [294]. Moreover, SLC-0111 combined with APX3330 has shown efficacy in inhibiting pancreatic cancer by targeting the Ref-1/APE1 pathway. [295]. Additionally, combined inhibition of CAIX activity and glutamine metabolism can lead to increased lipid peroxidation through the GSH/GPX4 axis, promoting ferroptosis (iron-dependent cell death) in tumor cells [296]. Furthermore, inhibition of CAIX disrupts the let-7/LIN28 axis and reduces glycolysis by targeting PDK1, which effectively inhibits breast cancer cell proliferation [297]. In lymphoma, a study using a syngeneic A20/BalbC mouse model demonstrated that the combination of CHOP chemotherapy with acetazolamide, a carbonic anhydrase inhibitor, significantly increased anti-tumor effects. This combination led to a 3–fourfold increase in CD3 + and CD8 + T-cell infiltration compared to CHOP treatment alone, suggesting that CA inhibitors like acetazolamide can enhance immune responses and support tumor-associated immunotherapies [294]. To enhance the therapeutic efficacy of anti-CAIX CAR-T cells for solid tumor treatment, researchers developed two CAR constructs: a second-generation CAR with an anti-CAIX G36 scFv, a 4-1BB costimulatory domain, and a CD3ζ activation signal (BBζ), and a third-generation CAR (28BBζ) incorporating both CD28 and 4-1BB modules. BBζ CAR-T cells showed over an eightfold increase in cytokine production (IL-2, GM-CSF, IFN-γ, TNF, and MIP-1α) compared to untransduced T cells (UNT), with smaller increases for other cytokines. Peripheral BBζ CAR-T cells also exhibited significantly lower PD-1 expression compared to 28ζ and 28BBζ CAR-T cells, potentially contributing to their superior antitumor activity. Gene expression analysis revealed that BBζ CAR-T cells had increased expression of MHC II (HLA-DRB1) and memory T cell-related genes (KLF6, IL-7R), along with decreased activation (GZMB) and exhaustion (NR4A2) markers. In an orthotopic mouse model of clear-cell renal cell carcinoma (ccRCC), BBζ CAR-T cells demonstrated robust therapeutic effects and persistence, highlighting their potential for effective solid tumor therapy [298].

A study explored the use of an OVs armed with decorin (OAV-Decorin) in combination with CAIX-targeting CAR-T cells for renal cancer treatment. This combination therapy effectively inhibited renal cancer cell growth and demonstrated enhanced antitumor efficacy compared to either treatment alone in vivo. The improved outcomes were attributed to the therapy’s ability to reduce collagen distribution in the extracellular matrix (ECM), inhibit TGF-β signaling, and increase INF-γ levels, collectively promoting a more favorable tumor microenvironment for CAR-T cell activity [299]. These studies highlight the therapeutic promise of targeting CAIX in combination with ICIs or CAR-T therapies to enhance immune responses, modify the TME, and improve patient outcomes in hypoxic solid malignancies.

##### Potential for applying CAi as the third agent in DDI

The application of CAIs as a third agent in DDI holds significant potential due to their unique ability to modify the tumor microenvironment, particularly under hypoxic conditions, which is common in many solid tumors. By targeting enzymes like CAIX and CAXII, CAIs help disrupt the acidic environment that often shields tumors from immune surveillance and limits the efficacy of immune therapies [300, 301]. This disruption allows for better immune cell infiltration, enhancing the performance of immunotherapeutic agents such as ICIs [300]. The feasibility of integrating CAIs into DDI is further supported by the early success seen in preclinical and clinical trials, where combinations with CAIs have shown increased tumor vulnerability, improved immune responses, and durable therapeutic effects, especially in resistant cancers like melanoma [302, 303].

The positive outlook for applying CAIs in DDI also lies in their capacity to offer a multi-faceted approach. They not only directly alter the tumor environment but also synergize with other therapies, potentially overcoming resistance mechanisms that tumors develop against ICIs [304]. Moreover, combining CAIs with DDI provides a more tailored approach to cancer treatment, particularly when paired with patient-specific biomarkers that can predict which tumors are most likely to benefit from CAXII or CAIX inhibition [273]. While challenges such as tumor heterogeneity and managing toxicity exist, the ability of CAIs to significantly boost immune responses, normalize the tumor microenvironment, and improve the effectiveness of existing immunotherapies underscores their promising role in the future of cancer treatment.

##### Potential challenges for conducting related study

Incorporating carbonic CAIs as a third agent in dual immunotherapy presents several significant challenges that must be overcome for successful integration into cancer treatment. One major issue is the substantial variability in carbonic anhydrase expression across different tumor types, making it difficult to predict which patients will benefit from CAI-based therapies [305]. For instance, while certain cancers like renal cell carcinoma and breast cancer show elevated levels of CAXII, other tumors may not express this enzyme to a meaningful degree [306]. This variability complicates patient selection, necessitating the development of more effective biomarkers and diagnostic tools to accurately identify patients likely to benefit from CAXII inhibition. Without precise methods for patient identification, there is a risk of administering ineffective treatment to those whose tumors do not depend on CAXII-driven mechanisms. Futhermorer, combining DDI with CAi adds complexity to treatment protocols. The pharmacokinetics, dosing, and toxicity profiles of these agents must be scrupulously managed [307]. The potential for increased side effects or overlapping toxicities is a real concern, particularly when combining multiple therapies that can provoke irAEs or metabolic disturbances [308]. Rigorous optimization of combination strategies and personalized approaches based on the tumor’s molecular profile are necessary to balance efficacy with tolerability [45].

In summary, while CAis offer a novel approach to enhancing immunotherapy, significant research and clinical investigation are required to address tumor heterogeneity, resistance mechanisms, and the potential for toxicity in combination therapies. These efforts will shape the future of personalized, more effective cancer treatment.

While novel third-agent sensitizers such as proliferating cell nuclear antigen inhibitors (PCNAis) and carbonic anhydrase IX inhibitors (CAis) demonstrate immunomodulatory potential, their efficacy as monotherapies remains limited, underscoring their optimal use as combination enhancers rather than standalone therapeutics. For PCNAis, preclinical studies—such as those using the novel mAb 14–25-9 in head and neck squamous cell carcinoma PDX models—have shown modest tumor growth inhibition (< 30%) as monotherapy, compared to > 70% when combined with NK cell-based therapies. Mechanistically, PCNAis function by disrupting the NKp44-PCNA immune checkpoint and interfering with DNA repair, thereby enhancing the cytotoxic activity of T and NK cells. Similarly, CAis like SLC-0111 have shown minimal survival benefit (< 20% median extension) as monotherapy in murine melanoma and breast cancer models, whereas their combination with ICIs has led to over 60% improvement in survival. The immunologic benefit of CAis stems from reconditioning the hypoxic, acidic tumor microenvironment, facilitating T cell infiltration and reducing MDSC-mediated suppression. Clinical trials have reported low response rates for PCNAis (10–25%) and CAis (< 15%) as monotherapies, often with transient benefits and resistance. These findings support their use in DDI + 1 strategies, where they can complement immune checkpoint blockade, TIL, or CAR-T therapies. The combination approach enables lower dosing, reduced toxicity, and mechanistic synergy while aligning with precision medicine goals to overcome tumor heterogeneity and resistance. Therefore, current evidence positions PCNAis and CAis not as primary therapeutics, but as rational sensitizers to enhance the efficacy of dual immunotherapy regimens Tables 10, 11and 12

**Table 10:** Prioritization of immunotherapeutic combinations by cancer type

Cancer type	Rank	Combination(s)	Supporting trials / evidence	LoE
Melanoma	1	ICI + OVs	Phase III MASTERKEY-265 (NCT02263508), Multiple Phase I/II trials (NCT01740297, NCT02414269)	1b
	2	ICI + TIL	Phase I/II trials (NCT03215810, NCT02621021), Multiple ongoing studies	2b
	3	ICI + CAR-T	Phase I studies (NCT01822652), Limited clinical data	3
NSCLC	1	ICI + RNA Vaccines	Phase II KEYNOTE-942 trial	2b
	2	ICI + OVs	Phase I study (NCT02043665, NCT02824965), 9% response rate	2b
	3	ICI + CAR-T	Phase I trials (NCT03726515, NCT03525782)	3
HCC	1	ICI + CAi	Preclinical HCC models, ongoing clinical interest	3
	2	ICI + OVs	Phase Ib recruiting (NCT05675462), Active trials	3
Breast Cancer	3	ICI + HDACi	Preclinical CXCL8/CXCR2 studies	4
	1	ICI + HDACi + ICI	Phase Ib study (NCT02453620), 25% objective response rate	2b
	2	ICI + OVs	Phase I study (NCT04185311), ongoing trials	2b
	3	ICI + CAR-T	Phase I ongoing (NCT03740256, NCT04995003)	3
Head & Neck Squamous Cell Carcinoma	1	ICI + RNA Vaccines	Phase II KEYNOTE-942 trial, 50% response rate	2b
	2	ICI + OVs	Phase Ib/III study (NCT02626000), 13.9% response rate	2b
	3	ICI + HDACi	Phase II trial (NCT04357873)	3
Urothelial Cancer	1	CAVATEK (CVA21) + pembrolizumab	20% ORR (3 CRs, 4 PRs), 17% sustained benefit	3
	2	Domatinostat (HDACi) + immunotherapy	Trials ongoing with promising safety data	2b
Renal Cell Carcinoma	1	ICI + ICI	CheckMate-214, FDA approved	1a
	2	CI + CAR-T (CAIX-targeted)	Phase I trials (NCT04969354), Anti-CAIX studies	3
	3	ICI + OVs	Phase I study (NCT03294083)	3
Glioblastoma	1	ICI + CAR-T	Phase I studies (NCT03726515, NCT04003649), 11.8 months median OS	3
	2	ICI + OVs	Phase I completed (NCT02798406)	3
	3	ICI + OVs	Phase I/II recruiting (NCT05084430)	3
Pancreatic Cancer	1	ICI + OVs	Phase Ib recruiting (NCT05303090)	3
	2	ICI + CAi	Phase Ib ongoing since 2018	3
	3	ICI + OVs	Phase II terminated (NCT03723915)	3
Colorectal Cancer	1	ICI + RNA Vaccines	Phase I/II terminated (NCT05456165)	3
	2	ICI + OVs	Phase I/II recruiting (NCT03206073)	2b
	3	ICI + CAR-T	Restored CAR-T function in liver metastases	4
Ovarian Cancer	1	ICI + CAR-T	Phase I study (NCT02414269), 23.9 months median OS	3
	2	ICI + OVs	Phase I recruiting (NCT05271318)	3
	3	ICI + TIL	Phase I/II completed (NCT03287674)	3

**Table 11:** Summary of combination immunotherapy strategies: mechanisms, preclinical findings, and clinical outcomes

Combination strategy	Key mechanisms	Key preclinical findings	Representative clinical result
ICI + OVs	OVs convert "immune desert" tumors into TIL-rich "hot" tumors OVs enhance immune cell infiltration into TME; OVs increase CD8^+^ T cell activation and reduce regulatory T cells; OVs encoding scFvs can target inhibitory immune checkpoints (YST-OVH)	OVs enhance ICIs efficacy in cold tumors; NDV shows synergistic effects with CTLA-4 inhibitor in colon cancer/melanoma; YST-OVH reduces exhausted PD-1hi CTLA-4^+^ TIM-3^+^ CD8^+^ T cells; Effective in poorly immunogenic tumors	T-VEC + pembrolizumab (NCT02263508): 62% ORR, 33% CR in melanoma; T-VEC + pembrolizumab (NCT02626000): 13.9% ORR in HNSCC; Enadenotucirev + nivolumab (NCT02636036): 2% ORR, 6% clinical benefit rate; CVA21 + pembrolizumab (NCT02043665): 9% ORR in NSCLC, 20% ORR in urothelial cancer
ICI + CAR-T	Targeting multiple tumor antigens simultaneously; Modifying immunosuppressive TME. Enhancing CAR-T cell trafficking and persistence; Improving CAR-T cell function and persistence; Reducing PD-1 on CAR-T cell surface	PD-1 knockdown in CAR-T cells enhances cytotoxicity; CAR-T cells with PD-L1 scFv antibody show enhanced activity; PD-L1 inhibition reduces CAR-T cell exhaustion; PD-L1 inhibitor counteracts liver MDSCs that compromise CAR-T function; Combination increases IFN-γ and TNF-α production	NCT01822652: Favorable safety with cyclophosphamide, fludarabine, and pembrolizumab; one CR; NCT02414269: Mesothelin-targeted CAR-T + pembrolizumab showed 23.9 months median OS (83% 1-year survival) in pleural mesothelioma; NCT03726515: EGFRvIII-targeted CAR-T + pembrolizumab showed 5.2 months PFS, 11.8 months OS in glioblastoma
ICI + TIL	Enhanced tumor recognition; Improved T cell function and persistence; Overcoming immunosuppressive TME; Reducing exhausted/suppressed T cells	CTLA-4 blockade improves TIL proliferation and CD8 + proportion; PD-1/CTLA-4 blockade increases TIL proliferation in murine colon/ovarian cancer; Triple blockade (PD-1/TIM-3/BTLA-4) enhances CD8^+^ PBMC expansion in melanoma ICIs counteract immunosuppressive TME in cholangiocarcinoma	NCT03215810: TIL + nivolumab in previous nivolumab-refractory patients: 3/13 achieved responses, 11/13 showed reduced tumor burden; NCT01174121: Phase II study showed complete regression in one patient with mutation-specific T cells persisting at 66 months; NCT03419559: Ongoing trial evaluating LN-145 alone or with durvalumab in anti-PD-1/PD-L1-naïve NSCLC
ICI + RNA-based Cancer Vaccines	Overcoming suppressive TME; Enhanced priming of T cell responses Converting "cold" tumors to "hot" tumors; Expanding cytotoxic T cell repertoire	Bi-adjuvant neoantigen nanovaccine + PD-1 blockade improves survival in colon cancer; Neoantigen vaccination + anti-PD-1 therapy achieves tumor regression via CD8 T cells; Combined therapy generates more robust and sustained T cell responses; More diverse T cell receptor repertoire	NCT02897765: NEO-PV-01 + PD-1 blockade showed 23.5 months median PFS in melanoma, 8.5 months in NSCLC, 5.8 months in bladder cance; NCT03289962: RO7198457 + atezolizumab achieved 8% ORR including one CR; NCT03313778: mRNA-4157 + pembrolizumab yielded 50% response rate in HNSCC (9.8 months median PFS)
CAR-T + OVs	Increased CAR-T cell trafficking/persistence; Modification of TME to support CAR-T function Enhanced tumor antigen release; Overcoming antigen escape; OVs expressing cytokines to attract T cells	OVs expressing RANTES and IL-15 enhance GD2-CAR-T cell infiltration; Oncolytic vaccinia virus with CXCL11 improves CAR-T cell efficacy; OVs with TNF-α and IL-2 synergize with CAR-T cells to prevent metastasis; OVs armed with bispecific T-cell engager (OAd-BiTE) enhance CAR-T activation	NCT03740256: Phase I trial evaluating binary OVs with HER2-targeting CAR-T cells in HER2 + solid tumors (ongoing); NCT05057715: Phase I evaluating huCART-meso with VCN-01 oncolytic virus expressing hyaluronidase
CAR-T + RNA-based Cancer Vaccines	RNA vaccines promote CAR-T cell proliferation Enhanced CAR-T cell killing efficacy; Prolonged maintenance of CAR-T effector functions; Promotion of memory formation; Overcoming suppressive TME	CLDN6-LPX vaccine promotes expansion and function of CLDN6 CAR-T cells; Lymph node-targeted vaccination increases CAR-T cell populations (up to 65% of total T cells); CAR-T cells with vaccine effectively eradicated glioblastomas, mammary tumors, and melanomas; Vaccine induces immune memory against new tumor challenges	NCT04503278: CLDN6 CAR-T cells alone or with CLDN6 RNA-LPX vaccine in advanced CLDN6 + tumors showed 43% ORR, 86% disease control rate 6/14 patients achieved partial remission 5/14 showed stable disease with target lesion shrinkage
OVs + TIL	OVs enhance activation and persistence of TILs; Converting tumor cells into antigen-presenting cells; Improving TME for TIL function Reducing immunosuppression; TNF-α and IL-2 armed OVs enhance TIL efficacy	OV-OX40L/IL12 transforms tumor cells into aAPCs, improving T cell activation; TILT-123 (OVs with TNF-α and IL-2) + TIL: 100% CR in hamster studies; IL-2-armed OVs promote tumor-specific TIL accumulation; Reduces proportion of exhausted PD-1^hi^ Tim-3^+^ CD8^+^ cells and Tregs	NCT04217473: TILT-123 + TIL showed 38% disease control rate; Complete response in one patient with ICI-resistant melanoma; Partial response in another (cutaneous) patient; Stable disease lasting > 10 months in two patients (uveal and cutaneous); Enhanced CD4^+^ and CD8^+^ T cell infiltration
OVs + RNA-based Cancer Vaccines	Overcoming immune escape resistance Releasing tumor antigens via oncolysis Enhancing dendritic cell activation Promoting robust tumor-specific adaptive immunity	VSV-IFNβ + vaccine targeting mutated CDSE1 gene reduced tumor size and prolonged survival; OVM significantly enhances DC vaccine antitumor effects; OVM-infected tumor oncolysates activate DC vaccines; Promotes CD8^+^ effector T cell infiltration in TME	Limited clinical trial data available for this specific combination; Research primarily in preclinical stage
PCNAi	Inhibition of DNA replication and repair; Targeting NKp44-PCNA immune checkpoint Enhanced NK cell antitumor responses; Activation of cGAS/STING pathway; Modulation of PI3K/Akt and MAPK pathways	mAb 14–25-9 blocks NKp44-PCNA checkpoint, enhancing NK cell function ATX-101 disrupts PCNA interactions and potentiates anti-cancer treatments AOH1160/AOH1996 suppress tumor growth PCNA-I1S enhances antiproliferative effects in prostate/lung cancer T2AA increases tumor sensitivity to platinum-based chemotherapeutic	NCT05227326: Phase I trial of AOH1996 in refractory solid tumors; Limited clinical data specific to DDI combinations
HDACi	Increased tumor antigen expression Enhanced immune cell infiltration into TME; Reduction of FoxP3 + Tregs Conversion of MDSCs to non-immunosuppressive phenotype; Suppression of CXCL8/CXCR2 axis	Entinostat + azacytidine with PD-1/CTLA-4 blockade eliminates tumors without metastasis; Entinostat + anti-PD-1/CTLA-4 improves tumor-free survival in breast and pancreatic models; HDAC3 inhibition suppresses CXCL8/CXCR2 axis; Reduces G-MDSC infiltration and enhances T cell activation	NCT02453620: Entinostat + nivolumab + ipilimumab achieved 25% ORR, 40% clinical benefit rate in advanced breast cancer; NCT03565406: Mocetinostat + ipilimumab/nivolumab showed 89% ORR at 70 mg dose; NCT04871594: Domatinostat + ICIs in muscle-invasive urothelial cancer; NCT04133948: Domatinostat + nivolumab or nivolumab + ipilimumab in melanoma
CAi	Neutralization of acidic TME; Improved immune cell infiltration Enhanced T cell function; Disruption of tumor pH regulation; Promotion of ferroptosis through GSH/GPX4 axis	SLC-0111 + anti-PD-1/CTLA-4 attenuates tumor growth and reduces metastasis; SLC-0111 creates hostile TME for TNBC, sensitizing tumors to ICB; Tiliroside (CAXIIi) reduces Tim-3 expression on depleted T cells; Acetazolamide + CHOP increases CD3 + /CD8 + T-cell infiltration in lymphoma; CAR-T cells targeting CAIX show robust therapeutic effects in ccRCC	NCT02215850: Phase I trial of SLC-0111 established safety and 1000 mg/day RP2D Phase Ib combining SLC-0111 with gemcitabine for pancreatic cancer; NCT05470283: OBX-115 (engineered TIL) + acetazolamide demonstrated durable responses in ICI-resistant melanoma; NCT04969354: CAIX-targeted CAR-T cells for refractory RCC
Other Candidates	Non-coding RNAs: Immune checkpoint regulation; piR-823-induced HMGB1 release miR-149-3p targeting PD-1, TIM-3, and BTLA; MTL-CEBPA reducing myeloid cell immunosuppression CAM: Modulation of immune system via natural compounds Enhancement of NK and DC activity Reduction of inflammation Dietary influence on microbiome and SCFA production	Non-coding RNAs: piR-823 promotes mitochondrial iron accumulation and AIM2-dependent inflammasome activation miR-149-3p reduces T-cell apoptosis and exhaustion markers; MTL-CEBPA upregulates C/EBP-α, enhancing anti-tumor activity CAM: Curcumin inhibits Treg activity and reduces IL-10/TGF-β expression Valeric acid acts as HDACi to improve anti-cancer immunity Flavonoids activate DCs and stimulate immune responses; Artemisinin derivatives reduce PD-L1 expression and increase CD8^+^ T-cell infiltration	Non-coding RNAs: NCT05727839: JCXH-211 (self-replicating RNA encoding IL-12) showed tumor lesion reductions (13–43%); NCT04105335/NCT05097911: MTL-CEBPA + pembrolizumab/atezolizumab for advanced solid tumors CAM: NCT03643289/NCT04193956: Mediterranean Diet adherence associated with 77% chance of ORR and 74% probability of 12-month PFS with ICB in melanoma; Case report: Chinese herbal medicine led to complete response in double-hit diffuse large B-cell lymphoma

**Table 12:** Advantages and disadvantage of cancer immunotherapies

	Immunotherapy Type	Advantages	Disadvantages
Individual Immunotherapy Modalities	ICIs	Durable long-term responses and immune memory formation; Effective across multiple cancer types; Less toxic than conventional chemotherapy; Can overcome tumor immune evasion mechanisms; FDA-approved for numerous cancer indications	Limited response rates (20–40% in most cancers); Primary and acquired resistance mechanisms irAEs; High treatment costs Ineffective in "cold" tumors with low immune infiltration; Pathway exhaustion after dual ICI failure
	OVs	Selective tumor cell targeting and lysis; Converts "cold" to "hot" tumors via immune cell recruitment; Releases tumor antigens to enhance immune recognition; Can be genetically modified to express therapeutic molecules; Local immunomodulation with systemic effects	Neutralizing antibodies limit repeated dosing; Variable viral replication efficiency; Manufacturing and delivery challenges; Limited clinical efficacy as monotherapy; Safety concerns with viral infection; Patient selection difficulties
	CAR-T Cell	Highly specific antigen targeting; Remarkable efficacy in hematological malignancies; Potential for long-lasting remissions Can be engineered for enhanced functionality; Personalized treatment approach	Limited efficacy in solid tumors; Severe toxicities (CRS, neurotoxicity); Manufacturing complexity and cost; Antigen escape and tumor heterogeneity Long production time (weeks); Limited to specialized centers
	TIL	Patient's own immune cells with natural tumor specificity; Proven efficacy in melanoma and certain solid tumors; Polyclonal response targeting multiple antigens Potential for memory formation; No genetic modification required	Complex and time-consuming manufacturing; High cost and limited availability; Requires sufficient tumor tissue for isolation; Variable TIL quality and expansion success; Limited to specialized centers; Lymphodepletion requirements;
	RNA-based Cancer Vaccines	Rapid development and manufacturing flexibility; Personalized neoantigen targeting; Enhanced immune responses against specific mutations; Safe profile with minimal toxicity; Can target multiple antigens simultaneously	Complex neoantigen identification process; Variable immunogenicity across patients; High development costs Limited clinical efficacy as monotherapy; Delivery and stability challenges; Requirement for advanced bioinformatics
DDI Combinations	ICI + OVs	Synergistic immune activation through multiple pathways; Converts “cold” to “hot” tumors; Enhanced T-cell infiltration and activation; Overcomes single-agent resistance mechanisms; Local and systemic immune responses	Optimal timing and sequencing challenges; Increased complexity in treatment protocols; Potential for enhanced toxicity; Patient selection difficulties; Manufacturing and coordination challenges
	ICI + CAR-T Cell	Targets multiple antigens and pathways Overcomes tumor microenvironment suppression; Enhanced CAR-T cell persistence and function; Potential for broader cancer applicability; Improved response rates	Severe toxicity risk (CRS, neurotoxicity, irAEs); Manufacturing complexity and cost Limited clinical experience; Unknown long-term effects; Requires specialized medical centers
	ICI + TIL	Enhanced tumor recognition and memory formation; Polyclonal response with ICI support; Overcomes immune checkpoint suppression Potential for durable responses; Natural tumor specificity	Complex manufacturing requirements; High costs and limited availability Variable TIL quality; Timing optimization challenges; Lymphodepletion requirements
	ICI + RNA Vaccines	Primes immune responses and removes checkpoints; Enhanced neoantigen-specific T-cell responses; Converts “cold” to “hot” tumors; Personalized treatment approach; Manageable safety profile	Complex development process; High costs for personalized vaccines; Variable patient responses; Neoantigen identification challenges; Limited clinical validation
	CAR-T + OVs	Enhanced CAR-T cell trafficking and persistence; Antigen release and immune activation; Overcomes immunosuppressive microenvironment; Addresses antigen escape mechanisms; Improved solid tumor efficacy potential	Viral immune responses affecting CAR-T cells; Complex manufacturing and delivery; Safety concerns with dual therapies Limited clinical experience; Patient selection challenges
	CAR-T + RNA Vaccines	Enhanced CAR-T cell proliferation and function; Memory formation and sustained responses; Antigen spreading effects; Overcome suppressive microenvironment; Reduced CAR-T cell dose requirements	Manufacturing complexity; High costs; Variable patient responses; Limited clinical validation; Timing optimization required
	OVs + TIL	Transforms “cold” to “hot” tumors; Enhanced TIL activation and persistence; Overcomes immunosuppression; Antigen release and presentation; Dual administration strategies	Complex manufacturing and timing; Variable viral replication; TIL quality dependencie; Limited clinical experience; Patient selection challenges
	OVs + RNA Vaccines	Overcomes immune escape resistance; Enhanced antigen presentation; Synergistic immune activation; Addresses predictable resistance mutations; Complementary mechanisms	Complex development process; Manufacturing challenges; Limited clinical validation; Optimal combination strategies unclear; Patient selection difficulties
“DDI + 1” Approaches	PCNAi	Disrupts DNA replication and repair; Activates cGAS/STING inflammatory pathways Enhances NK cell responses; Synergizes with multiple DDI combinations; Novel mechanism of action	Limited clinical experience; Potential for increased toxicity; Complex drug interactions; Patient selection challenges; Manufacturing and dosing optimization
	HDACi	Increases tumor antigen expression; Modulates tumor microenvironment; Reduces immunosuppressive cells; Proven safety in combination trials; Multiple available agent;	Potential for increased irAEs; Complex dosing and timing requirements; Variable patient responses; Drug interaction considerations; Limited biomarkers for selection
	CAi	Normalizes tumor pH environment; Enhances immune cell infiltration; Targets hypoxic tumor regions; Improves ICB efficacy; Well-established safety profile	Tumor heterogeneity in CA expression; Limited biomarkers for patient selection Potential metabolic side effects; Complex combination protocols; Variable efficacy across cancer types
Overall DDI Strategy Assessment	Clinical Potential	Addresses major limitations of monotherapies; Broader applicability across cancer types; Enhanced response rates and durability; Potential for “cure” in select patients; Paradigm shift in cancer treatment	No FDA-approved DDI regimens yet; Limited long-term safety data; High complexity and costs; Requires specialized infrastructure; Patient selection challenges
	Scientific Rationale	Complementary mechanisms of action; Addresses multiple resistance pathways; Strong preclinical evidence; Growing clinical trial pipeline; Potential for personalized medicine	Optimal combinations unclear; Limited understanding of interactions; Biomarker development needed; Manufacturing complexity; Regulatory pathway uncertainties

This table summarizes the key advantages and disadvantages based on current literature and clinical experience. Individual patient outcomes may vary, and ongoing research continues to refine our understanding of these therapeutic approaches

### Other potential candidates: miroRNA, complementary and alternative medicine

#### Non-coding RNAs

Non-coding RNAs (ncRNAs) are RNA molecules that are transcribed from the genome and do not code for proteins. In addition to their roles at the transcriptional and post-transcriptional levels, they play important roles in the epigenetic regulation of gene expression [309, 310]. NcRNAs include microRNAs(miRNAs), piwi-interacting RNAs (piRNAs), circular RNAs (circRNAs), small interfering RNAs(siRNAs) and so forth[311]. Recent study showed that siG12D-LODER, a siRNA preparation combined with gemcitabine, can inhibit the progression of locally advanced pancreatic cancer. Of the 15 patients treated, 12 had no tumor progression, the majority (10/12) showed stable disease, and 2 showed partial remission. The median OS was 15.12 months and the 18-month survival rate was 38.5%[312]. Further Phase II clinical trial (NCT01676259) are ongoing, demonstrating the potential of ncRNA in combination with chemotherapy. In addition to synergistic with chemotherapy, current studies also suggest that ncRNAs can serve as potential sensitizers for ICIs with strong immune-regulatory ability[313, 314], heralding the potential of ncRNAs as the third agent in DDI.

##### Current clinical trials of ncRNAs plus immunotherapy

Although there are no clinical trials on the combination of ncRNAs and dual immunotherapy, research on the use of RNAs in conjunction with immunotherapy has shown significant promise. In a phase I clinical trial (ClinicalTrials.gov Identifier: NCT05727839), 10 patients with advanced solid tumors were enrolled, including 3 with melanoma, 3 with breast cancer, 2 with head and neck cancer, 1 with nasopharyngeal carcinoma, and 1 with sarcoma. Treatment with JCXH-211, a self-replicating RNA encoding human IL-12, led to reductions in tumor lesions: 13% in a breast cancer patient, 33.3% in a head and neck cancer patient, and 43% in a melanoma patient[315]. These findings highlight the safety and anti-tumor potential of JCXH-211.Additionally, clinical trials investigating MTL-CEBPA, a small activating RNA (saRNA), in combination with pembrolizumab (ClinicalTrials.gov Identifier: NCT04105335) and atezolizumab (ClinicalTrials.gov Identifier: NCT05097911) for advanced solid tumors are ongoing. The results above to date demonstrate the potential of RNA-based therapies combined with ICIs as an effective approach to cancer therapy.

##### Potential mechanisms of applying ncRNAs as the 3rd agent for DDI

Previous studies have shown that RNAs can play an anti-tumor role through immune microenvironment and immune checkpoint regulation [313, 314]. For example, one study demonstrates that piR-823 promotes the accelerated degradation of PINK1, which is induced by the phosphatase and tensin homolog deleted on chromosome 10 (PTEN). This PINK1-PRKN pathway-mediated degradation of SLC25A37 and SLC25A28 leads to increased mitochondrial iron accumulation, resulting in a HIF1A-dependent Warburg effect and AIM2-dependent inflammasome activation in tumor cells. Additionally, AIM2-mediated release of HMGB1 induces the expression of CD274/PD-L1. As a result, piR-823 can function both as a tumor suppressor and as a sensitizer for ICIs [316, 317]. I Furthermore, certain miRNAs are thought to regulate multiple immune checkpoints, thereby exerting anti-tumor effects [314]. For instance, miR-149-3p has been shown to bind to the 3' untranslated regions (UTRs) of mRNAs encoding PD-1, TIM-3, and BTLA, downregulating their expression and preventing tumor immune escape [318]. Overexpression of miR-149-3p has also been found to reduce T-cell apoptosis and decrease markers of T-cell exhaustion, thereby enhancing CD8 + T cells' ability to target and destroy 4T1 breast cancer cells [318]. Moreover, MTL-CEBPA, a small activating RNA (saRNA), has been shown to reduce the immunosuppressive effects of myeloid cells and enhance the anti-tumor activity of ICIs by upregulating the transcription factor C/EBP-α (ClinicalTrials.gov Identifier: NCT04105335 and NCT05097911).These findings highlight the importance of immune regulation and immune checkpoint modulation as key mechanisms through which non-coding RNAs (ncRNAs) can be used in combination with immunotherapy. Pairing ncRNAs with immunotherapies offers significant potential to enhance their therapeutic effects.

#### Complementary and alternative medicine (CAM)

CAM encompasses a variety of medical systems and practices that exist alongside, and complement, conventional clinical medicine [319, 320]. It can be divided into two main categories. One is the systems with well-established theoretical and practical frameworks, such as Traditional Chinese Medicine, Indian Ayurveda, and European homeopathy [321]. Another one encompasses various medicinal and non-medicinal therapies, including herbal, fungal, animal, and mineral treatments, as well as acupuncture, qigong, thermotherapy, yoga, prayer, art appreciation, music therapy, and aerobic exercise [322, 323].

##### Management of irAEs

A significant challenge in DDI implementation is the increased incidence and severity of irAEs when multiple immunotherapeutic agents are combined. Specific CAM approaches have demonstrated efficacy in managing these toxicities without compromising anti-tumor immunity. For instance, acupuncture has shown remarkable effectiveness in treating ICI-induced Guillain-Barré syndrome resistant to conventional treatments like intravenous gamma globulin [324]. The mechanism likely involves vagus nerve-mediated immunomodulation through the brain-gut axis, providing neuroprotection while preserving anti-tumor immunity. Similarly, ginger extracts have demonstrated dual effects of reducing gastrointestinal inflammation while preserving cytotoxic T-cell function, potentially addressing colitis from ICIs without compromising therapeutic efficacy [325].

##### Overcoming resistance mechanisms

A major limitation of current DDI approaches is the development of primary and acquired resistance. Certain bioactive compounds from CAM show promise in targeting resistance pathways. Curcumin specifically targets STAT3 and PD-L1 pathways [326], directly counteracting key mechanisms through which tumors evade T-cell recognition. Artemisinin derivatives like dihydroartemisinin (DHA) upregulate IFN-γ expression within the tumor microenvironment while enhancing CD8^+^ T-cell expansion [327]., potentially resensitizing tumors that have developed resistance to PD-1/PD-L1 blockade [328]. These compounds could particularly benefit patients with "cold tumors" or those who have progressed after initial response to DDI.

##### Modulation of tumor microenvironment

The immunosuppressive tumor microenvironment remains a significant barrier to effective DDI therapy. Specific CAM approaches demonstrate unique abilities to reprogram this environment. Plant-derived polyphenols like epigallocatechin gallate (EGCG) from green tea have been shown to inhibit tumor-associated macrophage polarization toward the immunosuppressive M2 phenotype while promoting dendritic cell maturation and antigen presentation [329]. This dual action directly addresses the immunosuppressive milieu that limits DDI efficacy in solid tumors. Valeric acid, functioning as a natural HDAC inhibitor, synergistically enhances immunotherapy responses by epigenetically remodeling the tumor microenvironment to favor T-cell infiltration [270, 330].

##### Enhancement of therapeutic efficacy through dietary interventions

Nutritional approaches offer significant potential to enhance DDI outcomes. The Mediterranean Diet has demonstrated remarkable clinical benefits in patients receiving immunotherapy, with studies showing a 77% higher likelihood of objective response and 74% probability of 12-month progression-free survival in melanoma patients adhering to this dietary pattern [331, 332]. The mechanism involves modulation of the gut microbiome to increase short-chain fatty acid production, which serves as a biomarker for favorable immunotherapy response [333, 334]. These findings suggest that dietary interventions could be systematically integrated into DDI protocols to enhance efficacy without additional toxicity.

##### Improvement of patient quality of life and treatment adherence

The complicated administration schedules and cumulative toxicities of DDI regimens often lead to treatment discontinuation. Mind–body interventions including meditation, yoga, and tai chi have been shown to reduce cancer-related fatigue, improve sleep quality, and decrease anxiety in cancer patients undergoing immunotherapy [19, 335]. These benefits directly translate to improved treatment adherence and ability to tolerate full therapeutic courses, potentially improving overall survival outcomes from DDI approaches.

The integration of these CAM approaches into DDI protocols presents a novel opportunity to address key limitations while potentially enhancing therapeutic outcomes. Future clinical trials specifically evaluating CAM components as adjuncts to DDI strategies are warranted to establish standardized protocols that maximize patient benefit.

#### Neutralizing tumor acidity

Acidic TMEs, primarily resulting from increased glycolysis (the Warburg effect), lactate accumulation, and carbonic anhydrase overexpression, pose a substantial barrier to effective antitumor immunity by suppressing T cell activation, promoting Treg expansion, impairing DC function, and inducing M2 polarization in tumor-associated macrophages [336]. Tumor acidity also compromises NK cell cytotoxicity and antigen presentation by APCs, collectively fostering an immunosuppressive niche. Agents that neutralize tumor acidity—including CAIs, proton pump inhibitors (PPIs), lactate transport blockers, and systemic buffering agents like oral bicarbonate—have shown promise in reprogramming the TME to restore immune cell activity and sensitize tumors to immunotherapy. For instance, inhibition of CAIX or CAXII elevates peritumoral pH, enhances CD8⁺ T cell infiltration, and synergizes with PD-1 blockade in preclinical models of HCC and triple-negative breast cancer [337]. The CAIX inhibitor SLC-0111, currently under clinical evaluation, has shown improved immune cell infiltration and enhanced efficacy of checkpoint inhibitors in solid tumors. Similarly, the combination of TIL therapy with acetazolamide in the OBX-115 trial has demonstrated encouraging clinical responses in ICI-resistant melanoma. PPIs such as omeprazole and lansoprazole have been repurposed in cancer models to alkalinize the TME by inhibiting V-ATPase activity, which reduces proton extrusion from cancer cells. This effect improves cytotoxic T lymphocyte activity and sensitizes tumors to anti-CTLA-4 and anti-PD-1 therapies, as shown in murine models of melanoma and gastric cancer. Oral bicarbonate supplementation, a low-cost and widely accessible intervention, has demonstrated the ability to raise intratumoral pH and potentiate the effects of anti–PD-1 therapy in mouse models of melanoma, pancreatic ductal adenocarcinoma, and prostate cancer [338]. Notably, in a B16 melanoma model, bicarbonate pre-treatment significantly improved the efficacy of adoptive T cell transfer. Lactate transporter inhibitors such as AZD3965 (targeting MCT1) reduce lactate-driven acidification and restore CD8⁺ T cell effector function, while simultaneously depriving cancer cells of a key metabolic substrate [339]. These agents not only facilitate immune infiltration and cytotoxicity but also improve tumor visibility to effector lymphocytes through enhanced antigen processing and presentation. Emerging strategies—including pH-sensitive nanoparticles, engineered bacteria that release bicarbonate, and tumor-selective delivery of buffering agents—further highlight the translational potential of TME alkalinization [340]. Collectively, acid-neutralizing agents represent a mechanistically robust and clinically promising class of immunotherapy sensitizers, particularly for “cold” or immune-excluded tumors that lack sufficient T cell infiltration. Their integration into DDI or DDI + 1 regimens could help overcome primary and acquired resistance to immune checkpoint inhibitors, warranting further clinical investigation and rational combination design.

## Triple immunotherapy strategies in cancer treatment

The emerging field of triple immunotherapy strategies represents a natural evolution and expansion of the DDI concept, offering even more comprehensive approaches to overcome resistance mechanisms and enhance therapeutic efficacy. Recent groundbreaking studies have demonstrated that strategic combinations of three distinct immunotherapeutic modalities can achieve synergistic effects that surpass dual combinations, particularly in traditionally resistant cancers. For instance, a study showed that combining CD40 agonists with PD-1 and CTLA-4 blockade reprograms non-immunogenic tumor microenvironments, enhancing T-cell priming and effector functions [341]. This strategy increases lymphocyte infiltration, polarizes macrophages toward M1 phenotypes, and reduces immunosuppressive regulatory T cells, yielding notable efficacy in pancreatic cancer. Similarly, another study reported that co-targeting 4-1BB and LAG-3 on T cells, along with CXCR1/2 inhibition in myeloid cells, elicits robust antitumor immunity and remodels the immune landscape in pancreatic ductal adenocarcinoma [342]. In glioblastoma, neoadjuvant administration of nivolumab, relatlimab, and ipilimumab demonstrated sustained tumor control and immune activation in a patient with newly diagnosed disease, marking a major therapeutic breakthrough in this notoriously resistant malignancy [343].

Beyond checkpoint blockade, triplet strategies that integrate immunotherapy with targeted therapies and chemotherapy are under active investigation. Trials such as pembrolizumab + trastuzumab + chemotherapy in HER2-positive breast cancer and atezolizumab + bevacizumab + chemotherapy in solid tumors harness the immunomodulatory effects of cytotoxic agents alongside immune checkpoint inhibition [344]. In triple-negative breast cancer, combinations like pembrolizumab + olaparib + chemotherapy exploit DNA damage-induced neoantigen formation to amplify immune responses [345]. Furthermore, novel regimens combining oncolytic viruses, CAR-T cells, and checkpoint inhibitors leverage complementary mechanisms to enhance tumor antigen release, immune cell trafficking, and sustained T-cell activity, with promising outcomes in preclinical studies. Furthermore, novel regimens combining oncolytic viruses, CAR-T cells, and checkpoint inhibitors leverage complementary mechanisms to enhance tumor antigen release, immune cell trafficking, and sustained T-cell activity, with promising outcomes in preclinical studies. Recent advances include blockade of CCR5⁺ T cell accumulation in the tumor microenvironment to optimize anti-TGF-β/PD-L1 bispecific antibody therapy [346]. Mechanistically, triplet approaches offer superior capacity to target multiple resistance pathways, foster immune memory formation, and remodel suppressive tumor microenvironments by modulating Tregs, MDSCs, cytokines, and metabolic barriers.

As triple immunotherapy transitions from preclinical promise to clinical application, key considerations include optimal patient selection, safety management, and regulatory coordination. Biomarker-driven stratification—based on immune profiling and tumor characteristics—will be essential for identifying responders and minimizing toxicity. While enhanced efficacy is encouraging, triple regimens pose increased risks of irAEs, necessitating refined dosing schedules, sequential administration strategies, and vigilant monitoring. Integration into the existing DDI framework is exemplified by the concept of triple distinct immunotherapy, which expands upon successful dual combinations by introducing synergistic third agents. With continued innovation and careful clinical translation, triple immunotherapy holds transformative potential for improving outcomes in resistant and aggressive cancers.

## Conclusion

**Fig. 10:**
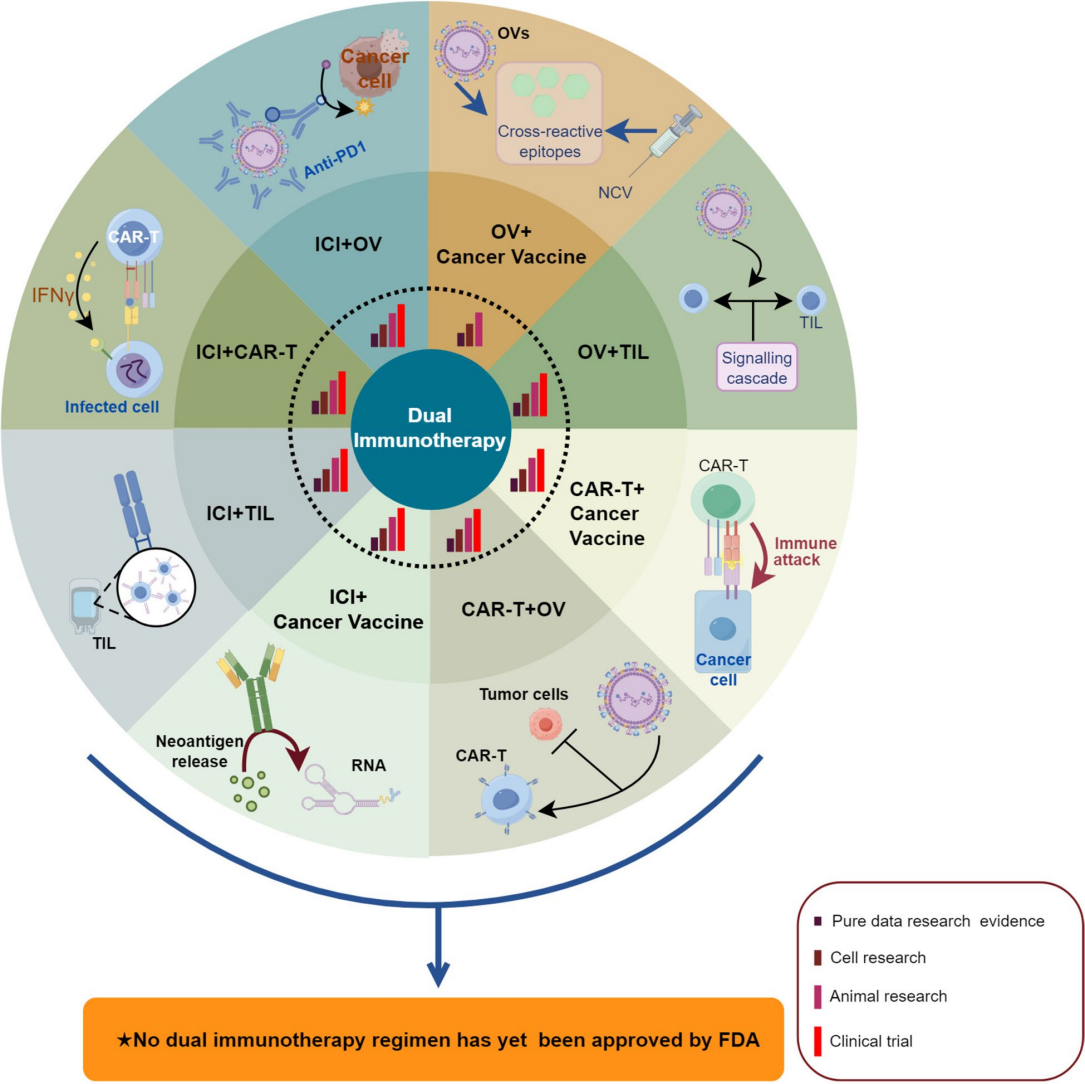
Level of Evidence in DDI Development: This bar chart illustrates the research progress of different DDI strategies. Among them, the combination of OVs and RNA-based cancer vaccines is progressing relatively slowly, remaining at the animal experiment stage. Additionally, it is worth noting that no Dual Immunotherapy has been approved by the US FDA to date

Here, this review is the first to bring about the concept of DDI and underscores the unparalleled potential of DDI in redefining systemic cancer treatment. DDI exhibits advantages over existing therapies by synergistically enhancing immune responses through diverse mechanisms, potentially addressing critical challenges such as therapeutic resistance and limited efficacy of monotherapies. Among its most transformative features is its superior ability to convert "cold tumors"—characterized by low immune cell infiltration and poor responsiveness to immunotherapy—into "hot tumors," fostering an immunologically active microenvironment. This capability broadens the scope of immunotherapy to include a wider range of cancer types and patient populations, thereby overcoming major barriers in current oncology. The value of DDI lies in its integration of complementary immunotherapeutic modalities, creating a robust and adaptive immune system response. By engaging multiple pathways, DDI not only enhances tumor recognition and elimination but also contributes to long-lasting immune memory, reducing recurrence rates. Clinically, DDI offers a groundbreaking approach to personalizing cancer therapy, with promising applications in combination with cutting-edge strategies such as OVs, CAR-T cells, tumor-infiltrating lymphocytes, and neoantigen cancer vaccines. Moreover, the transformative potential of DDI extends beyond improving survival outcomes; it represents a paradigm shift in cancer treatment. By leveraging its ability to reshape the tumor microenvironment and surmount resistance mechanisms, DDI holds the promise of achieving durable responses in even the most challenging cases. Future research and clinical applications of DDI are poised to revolutionize oncology, offering hope for improved quality of life and survival for cancer patients worldwide (Fig. 10).

Additionally, as an emerging field, DDI offers vast potential for exploration. Particularly in the selection of the third agent. Incorporating a third agent into DDI presents a significant opportunity to enhance its therapeutic effectiveness by tackling resistance mechanisms, expanding tumor responsiveness, and achieving synergistic effects through multi-faceted approaches. Resistance, often driven by immune checkpoint exhaustion or adaptations within the tumor microenvironment, can potentially be mitigated by introducing complementary agents such as TKIs, anti-angiogenic drugs, or vaccines that target alternative pathways. This strategy may also broaden the scope of DDI, making it effective against less immunogenic “cold tumors” by enhancing immune cell recruitment and improving antigen presentation. Clinically, the inclusion of a third agent has demonstrated the potential to further improve key outcomes, including ORR, PFS, and OS, while reducing relapse rates and achieving more durable treatment responses. Additionally, this approach enables personalized treatment plans tailored to specific tumor biomarkers and individual patient characteristics. Future research should prioritize optimizing the sequencing, timing, and dosing of third agents in DDI regimens, exploring novel immune or metabolic pathways, and extending these strategies to resistant cancers such as pancreatic, ovarian, and urothelial cancers. Efforts to enhance affordability and streamline production processes, particularly for cell- and vaccine-based therapies, will also be essential for broad clinical adoption. Integrating a third agent into DDI represents a promising advancement in cancer treatment, offering a pathway to overcome current limitations, address resistance, and improve long-term patient outcomes.

Notably, while none of the DDI strategies or “DDI + 1” approaches summarized and analyzed in this work have yet received FDA approval, the field has already amassed a substantial body of preclinical studies and ongoing clinical trials. Therefore, this area undoubtedly warrants greater effort and attention moving forward. In other word, building upon the significant progress outlined, it is clear that DDI regimens hold immense transformative potential in cancer treatment. By effectively combining distinct immunotherapeutic strategies, DDI has demonstrated the ability to broaden the range of treatable cancers, address resistance mechanisms, and improve treatment outcomes in both preclinical and clinical studies. While the field is still in its early stages, the substantial body of preclinical research and ongoing clinical trials underscores the importance of continued exploration in this area. The integration of a third agent into DDI not only enhances therapeutic efficacy but also represents a strategic approach to overcoming resistance and expanding the application of immunotherapy to less responsive cancers. Future research should focus on optimizing combination strategies, exploring novel therapeutic targets, and addressing challenges such as patient selection, treatment sequencing, and potential side effects. The clinical and scientific significance of this work lies in its potential to provide long-term solutions for challenging cancers, improving survival rates and quality of life for patients worldwide. As a rapidly evolving field, DDI demands greater attention and investment to realize its full potential. By encouraging collaboration across disciplines and advancing innovative research, the future of oncology may see a paradigm shift, where DDI becomes a cornerstone of cancer treatment. This work contributes to laying a strong foundation for future advancements in DDI, inspiring further breakthroughs in the fight against cancer.

## Data Availability

No datasets were generated or analysed during the current study.

## Abbreviations

ACS: American Cancer Society
AEs: Adverse events
BBζ: Second-generation CAR with anti-CAIX G36 scFv, 4-1BB costimulatory domain, and CD3ζ activation signal
CAIX: Carbonic anhydrase IX
CAIs: Carbonic anhydrase inhibitors
CAM: Complementary and Alternative Medicine
CAR-T: Chimeric antigen receptor T-cell therapy
CAXII: Carbonic anhydrase XII
CAi: Carbonic anhydrase inhibitor
CCA: Cholangiocarcinoma
CIK: Cytokine-induced killer
CR: Complete response
CRS: Cytokine release syndrome
DC: Dendritic cell
DDI: Dual Distinct Immunotherapy
DHA: Dihydroartemisinin
ECM: Extracellular matrix
EGCG: Epigallocatechin gallate
EGFR: Epidermal growth factor receptor
EMT: Epithelial-mesenchymal transition
FDA: Food and Drug Administration
G-MDSCs: Granulocytic myeloid-derived suppressor cells
HBV: Hepatitis B virus
HCC: Hepatocellular carcinoma
HDACi: Histone deacetylase inhibitor
HNSCC: Head and neck squamous cell carcinoma
HR: Homologous recombination
HSV-1: Herpes simplex virus 1
IARC: International Agency for Research on Cancer
ICANS: Immune effector cell-associated neurotoxicity syndrome
ICB: Immune checkpoint blockade
ICI: Immune checkpoint inhibitor
IFN-γ: Interferon gamma
IFNs: Interferons
IL: Interleukin
ITIM: Immunoreceptor tyrosine-based inhibitory motif
KIR: Killer immunoglobulin-like receptor
L-MDSCs: Liver myeloid-derived suppressor cells
MedD: Mediterranean Diet
NDV: Newcastle disease virus
NK: Natural killer
NSCLC: Non-small cell lung cancer
OR: Overall response
ORR: Objective response rate
OS: Overall survival
OV: Oncolytic virus
OVs: Oncolytic viruses
PBMC: Peripheral blood mononuclear cell
PBMCs: Peripheral blood mononuclear cells
PCNA: Proliferating Cell Nuclear Antigen
PCNAis: Proliferating Cell Nuclear Antigen inhibitors
PFS: Progression-free survival
PR: Partial response
R/M HNSCC: Recurrent or metastatic squamous cell carcinoma of the head and neck
RCC: Renal cell carcinoma
RFS: Recurrence-free survival
RNA-LPX: RNA-lipoplex vaccine
RP2D: Recommended Phase 2 Dose
RPB1: RNA polymerase II
SCFAs: Short-chain fatty acids
SD: Stable disease
SVV: Seneca Valley Virus
T-VEC: Talimogene Laherparepvec
T3: 3,3′,5-Triiodothyronine
TAAs: Tumor-associated antigens
TCR: Transcription-coupled repair
TIL: Tumor-infiltrating lymphocyte
TKIs: Tyrosine Kinase Inhibitors
TME: Tumor microenvironment
TNBC: Triple-negative breast cancer
TNF: Tumor necrosis factor
TRAEs: Treatment-Related Adverse Events
Tregs: Regulatory T cells
UTRs: Untranslated regions
VEGFR: Vascular endothelial growth factor receptor
VSV-IFNβ: Interferon beta-expressing lysosomal vesicular stomatitis virus
aAPCs: Artificial antigen-presenting cells
ccRCC: Clear-cell renal cell carcinoma
circRNAs: : Circular RNAs
irAE: : Immune-related adverse event
mAb: Monoclonal antibody
miRNAs: MicroRNAs
ncRNA: Non-coding RNA
ncRNAs: Non-coding RNAs
pY211-PCNA: Tyrosine 211 phosphorylation of PCNA
piRNAs: Piwi-interacting RNAs
saRNA: Small activating RNA
scFv: Single-chain variable fragment
siRNAs: Small interfering RNAs
ssDNA: Single-stranded DNA
